# Genome-wide identification and functional characterization of natural antisense transcripts in *Salvia miltiorrhiza*

**DOI:** 10.1038/s41598-021-83520-6

**Published:** 2021-02-26

**Authors:** Mei Jiang, Haimei Chen, Jingting Liu, Qing Du, Shanfa Lu, Chang Liu

**Affiliations:** 1 Key Laboratory of Bioactive Substances and Resource Utilization of Chinese Herbal Medicine From Ministry of Education, Engineering Research Center of Chinese Medicine Resources From Ministry of Education, Institute of Medicinal Plant Development, Chinese Academy of Medical Sciences, Peking Union Medical College, Beijing, 100193 People’s Republic of China; 2 College of Pharmacy, Key Laboratory of Plant Resources of Qinghai-Tibet Plateau in Chemical Research, Qinghai Nationalities University, Xining, 810007 Qinghai People’s Republic of China

**Keywords:** Genetics, Plant sciences

## Abstract

*Salvia miltiorrhiza* is one of the most widely used traditional medicines. Natural antisense transcripts (NATs) are a class of long noncoding RNAs that can regulate gene expression. Here, we identified 812 NATs, including 168 *cis*-NATs and 644 *trans*-NATs from twelve root, flower, and leaf samples of *S. miltiorrhiza* using RNA-seq. The expression profiles for 41 of 50 NATs and their sense transcripts (STs) obtained from RNA-Seq were validated using qRT-PCR. The expression profiles of 17 NATs positively correlated with their STs. GO and KEGG pathway analyses mapped the STs for *cis*-NATs to pathways for biosynthesis of secondary metabolites. We characterized four NATs in detail, including NAT0001, NAT0002, NAT0004, and NAT00023. Their STs are kaurene synthase-like 1 and the homologs of UDP-glucose flavonoid 3-O-glucosyltransferase 6, UDP-glycosyltransferase 90A1, and beta-glucosidase 40, respectively. The first gene is involved in the biosynthesis of bioactive tanshinones, the next two are involved in anthocyanin biosynthesis, whereas the last is involved in phenylpropanoid biosynthesis. Besides, we found seven STs that are potential targets of miRNAs. And we found two miRNAs including miR156a and miR7208, might originate from NATs, NAT0112 and NAT0086. The results suggest that *S. miltiorrhiza* NATs might interact with STs, produce miRNAs, and be regulated by miRNAs. They potentially play significant regulatory roles in the biosynthesis of bioactive compounds.

## Introduction

Natural antisense transcripts (NATs) are a class of long noncoding RNAs, which transcribe in the opposite direction of protein-coding genes^1^. Relatively, the transcripts of protein-coding genes were called sense transcripts (STs). Base on the position of NATs and their STs, NATs can be divided into two categories, namely, *cis*-NATs and *trans*-NATs^2,3^. *Cis*-NATs originated from the same genomic loci with STs. In contrast, *trans*-NATs originated from different genomic loci with STs. NATs were initially detected in bacteria^4^, then they were found in prokaryotes^5^ and eukaryotes^6^. With the recent development of high-throughput DNA sequencing technologies and bioinformatics analysis methods, genome-wide NATs have been reported in various plants, such as *Arabidopsis thaliana*^7^, *Oryza sativa*^8^, *Zea mays*^9^, *Saccharum officinarum*^10^, and *Petunia hybrida*^11^.

It is found that NATs play an essential role in regulating gene expression through different mechanisms^12^, such as mechanisms related to transcriptional interference^13^, RNA–DNA interactions^14^, RNA–RNA interactions in the nucleus^15^, and RNA–RNA interactions in the cytoplasm^16^. In *Arabidopsis thaliana*, a *cis*-NAT producing from the opposite strand of *MADS AFFECTING FLOWERING4* (*MAF4*) gene can positively regulate the expression of its ST *MAF4*, thereby affecting flowering time. It activates *MAF4* by recruiting WDR5a to *MAF4* to enhance histone 3 lysine 4 trimethylation (H3K4me3)^17^. In *Oryza sativa*, a *cis*-NAT was produced from an *R2R3-MYB* gene locus and suppressed the *R2R3-MYB* by mediating chromatin modifications^18^. In mutant plants with downregulation of the NAT by RNA interference (RNAi), the expression level of *the R2R3-MYB* transcript significantly increases, resulting in twisted leaf blades. Besides, a NAT originates from the *CYCLING DOF FACTOR 5* (*CDF5*) gene locus in *Arabidopsis thaliana*. In mutant plants with a T-DNA insertion in non-overlapping regions of *CDF5*, the expression level of *CDF5* transcript was strongly reduced compared with wild plants, while its NAT increased 2–4 folds^19^.

*Salvia miltiorrhiza* Bunge is one of the most important traditional medicines from the Lamiaceae family. Tanshinones is one of the major active compounds in *S. miltiorrhiza* and are used to treat cardiovascular diseases through anti-atherosclerosis^20^, anti-oxidation^21^, etc. Besides, this plant also produces many flavonoids^22^, such as rutin, isoquercitrin, and astragalin. Various genes in the biosynthetic pathway of tanshinones have been identified in *S. miltiorrhiza*. These include the *SmCPS* gene encoding copalyl diphosphate synthase, *SmKSL* gene encoding *ent*-kaurene synthase^23^, *SmCYP76AH1* gene encoding cytochrome P450 enzymes^24^, etc. The whole-genome^25,26^, transcriptome^27–29^, and microRNAs^30–32^ of *S. miltiorrhiza* have been reported, respectively. However, a systematic analysis of NATs is not yet available for *S. miltiorrhiza*. Here, we conducted a genome-wide identification of NATs in *S. miltiorrhiza*. The results suggest that NATs might interact with different types of molecules such as the STs and miRNAs, and consequently play an important role in a wide range of biological processes such as the biosynthesis of active compounds and glycosylation.

## Results

### Global identification of NATs in *Salvia miltiorrhiza*

To identify the NATs in *S. miltiorrhiza*, we carried out strand-specific RNA-seq (ssRNA-seq) analysis of 12 RNA samples extracted from three tissues, including roots (r), flowers (f), and leaves (l). We obtained 25.3, 53.8, and 26.7 million paired-end reads from pooled flower, leaf, and root samples. And we obtained 44.5 (p01_f), 43.4 (p02_f), 42.9 (p03_f), 42.5(p01_l), 44.2 (p02_l), 41.6 (p03_l), 44.2 (p01_r), 41.9 (p02_r) and 40.9 (p03_r) million paired-end reads (Table S1).

**Figure 1:**
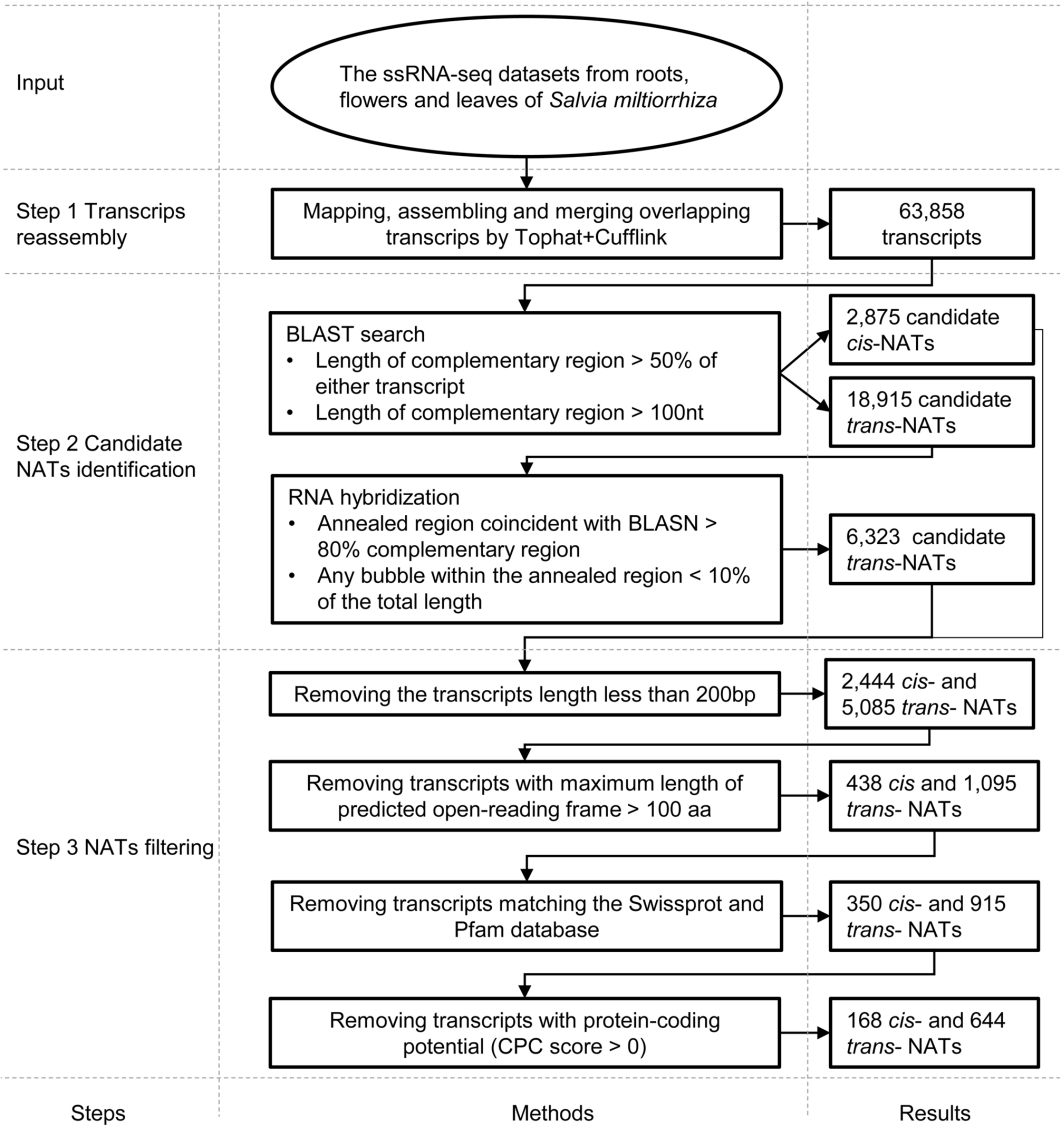
The bioinformatics pipeline used to identify NATs in *S. miltiorrhiza*. The figure has three columns. The main steps of the pipeline are shown in the left column. The detailed analytical processes are shown in the middle column. The results produced by a particular process are shown in the right column.

We identified the NATs using the bioinformatic pipeline shown in Fig. 1. In step 1, 83.5%, 79.2%, and 84.3% reads from flowers, leaves, and roots, respectively, were mapped to the *S. miltiorrhiza* reference genome sequence^25^. We assembled a total of 63,858 transcripts. Among them, 30,478 were previously annotated protein-coding transcripts in the reference genome. Using an FPKM cutoff value of 1, we found a total of 44,596 transcripts, including 22,099 previously annotated protein-coding transcripts expressed in at least one of the three tissues. In step 2, we identified 21,790 candidate NATs. It includes 2,875 candidate *cis*-NATs and 18,915 candidate *trans*-NATs. Then, we identified 6,323 candidate *trans*-NATs based on the annealing potential of complementary regions. In step 3, we obtained 2,444 candidate *cis*-NATs and 5,085 candidate *trans*-NATs with a sequence length of more than 200 bp. These candidate NATs were subject to a series of filtering steps based on noncoding RNA identification. After removing transcripts with the maximum length of predicted open-reading frame ≥ 100 aa, transcripts matching the protein database, and transcripts with protein-coding potential (CPC score > 0), we finally annotated 812 NATs, which include 168 *cis*-NATs and 644 *trans*-NATs (Table S2, Sfile 1). The number of *cis*-NATs and *trans*-NATs correspond to 0.55% (168/30,478) and 2.11% (644/30,478) of predicted protein-coding genes, respectively.

### Characterization of NATs in *S. miltiorrhiza*

**Figure 2:**
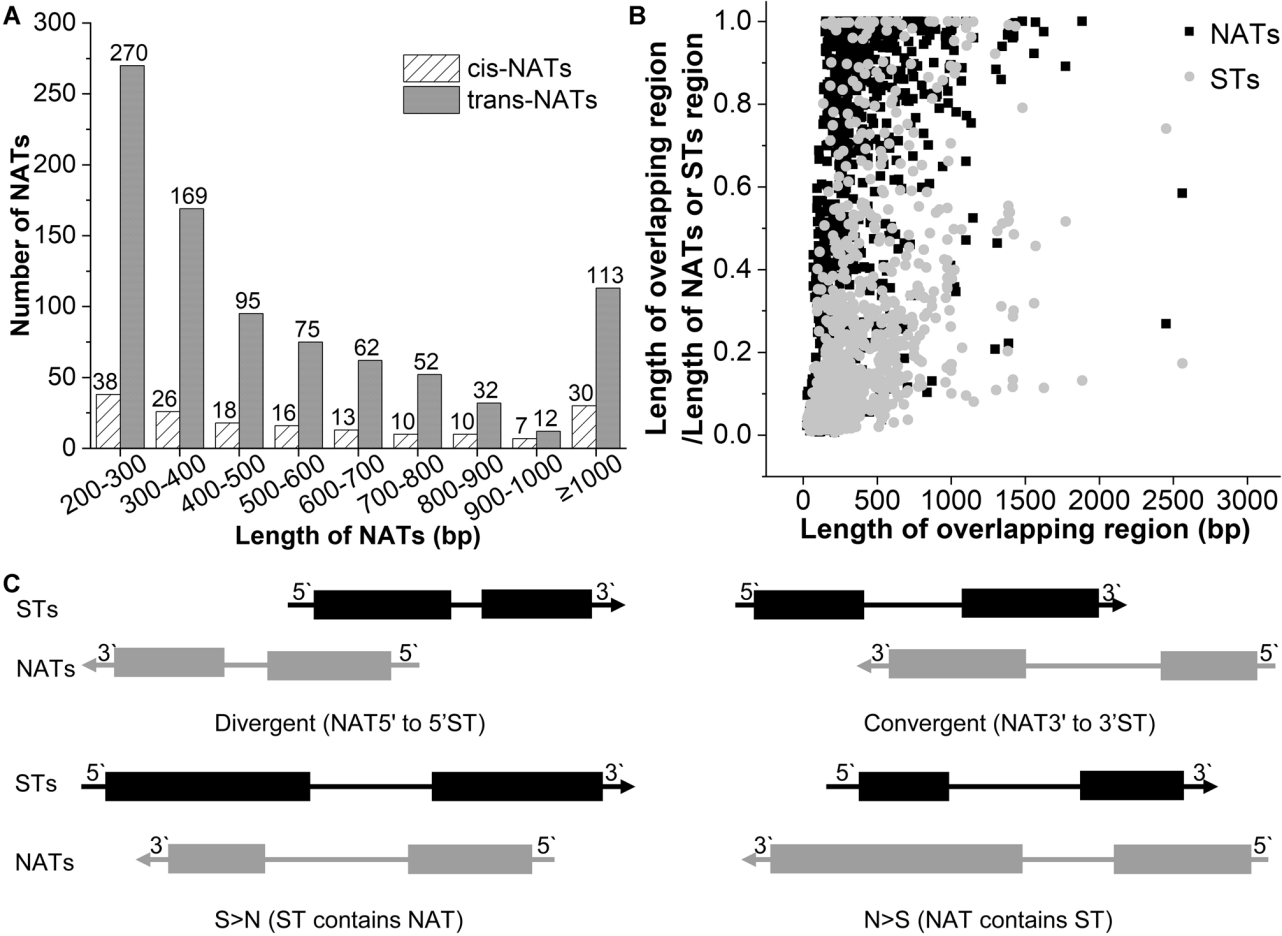
Characterization of *S. miltiorrhiza* NATs. (**A**) Length distribution of NATs. (**B**) Comparison of the proportion of the length of overlapping regions to that of STs and NATs with the length of the overlapping region. (**C**) Schematic representation of different types of *cis*-NATs based on the location relative to the STs. STs and NATs were depicted as gray and black bars, respectively. Arrows indicated the directions of transcription.

We analyzed the features of the identified NATs. It includes average size, exon number, and the length of overlapping regions. The length of *cis*-NATs ranged from 200 to 2641 bp, with an average size of 647.16 bp. The length of *trans*-NATs ranged from 200 to 4341 bp with an average size of 545.62 bp. The majority length distribution ranged from 200 to 300 bp in both *cis*-NATs and *trans*-NATs (Fig. 2A). There were also some NATs with lengths longer than 1000 bp. In general, the length distribution of *cis*-NATs and *trans*-NATs was similar. However, the number of exons in *cis*-NATs (the average number was 2.07) was more than that in *trans*-NATs (the average number was 1.10).

The mapping relationships between NATs and STs were found to be multiplex^33^. Some NATs are mapped to multiple STs. On the other hand, some STs were mapped to multiple NATs. This phenomenon was also observed in the SAT pairs (ST and NAT pairs) of *S. miltiorrhiza*. We classified the mapping relationships of SAT pairs into four types, including “1 versus 1”, “1 versus n”, “n versus 1”, and “n versus n” (Table S2). “1 versus 1” represents one NAT mapped to one ST, “1 versus n” represents one NAT mapped to multiple STs, “n versus 1” represents multiple NATs mapped to one ST, and “n versus n” represents multiple NATs mapped to multiple STs. We found a total of 1045 SAT pairs corresponding to 168 *cis*-NATs and 644 *trans*-NATs. For *cis*-NATs, 134 and 34 formed 1 versus 1 and n versus 1 relationship, respectively. However, we found no 1 versus n and n versus n relationships for *cis*-NATs. For *trans*-NATs, 411, 159, 155, and 152 formed 1 versus 1, 1 versus n, n versus 1, and n versus n relationships. The multiplex relationships are most likely due to duplicate genes in the genome. Then, we examined the proportion of the length of overlapping regions between STs and NATs (Fig. 2B). In general, the average proportion of the overlapping region to NATs (73.08%) is larger than that of STs (53.37%).

We classified the *cis*-NATs into four types based on the location relative to the STs. It includes convergent (with 3′-ends overlapping), divergent (with 5′-ends overlapping), STs containing NATs (S > N), and NATs containing STs (N > S) (Fig. 2C). Among the 168 *cis*-NATs, 40 were divergent, 60 were convergent, 60 were STs containing NATs, and 8 were NATs having STs. The primary type was convergent and STs containing NATs.

### Conservation of NATs identified in *S. miltiorrhiza*

To determine the conservation of NATs identified in *S. miltiorrhiza*, we searched the PlncRNADB (http://bis.zju.edu.cn/PlncRNADB/index.php) and GreeNC^34^ databases for homologs. A total of 4 NATs, NAT0030, NAT0047, NAT0131, and NAT0346, were found to be conserved among the species of *Medicago truncatula* and *Mimulus guttatus* (Table 1).

**Table 1:** List of conserved NATs identified in *S. miltiorrhiza*.

NAT ID	ID in database	Identity level	Alignment length	Start of alignment in query	End of alignment in query	Start of alignment in subject	End of alignment in subject
NAT0030	Mguttatus_Migut.M00719.1	78.472	144	4	143	91	233
NAT0047	Mguttatus_Migut.N03196.1	83.333	270	192	461	263	532
NAT0131	Mguttatus_Migut.J00829.1	88.341	223	77	299	78	300
NAT0346	Mtruncatula_Medtr2g041250.1	77.953	127	55	176	72	195

### Functional enrichment analyses of the STs having cis-NATs

To explore NATs’ putative function, we analyzed Gene Ontology (GO) enrichment and Kyoto Encyclopedia of Genes and Genomes (KEGG) pathway^35^ analyses on all the STs having *cis*-NATs in three tissues analyzed. Almost all STs were mapped to GO terms. The STs were mapped to the subgroups of the biological processes, the cellular components subgroup, and the molecular functions subgroup counted for 68.1%, 94.1%, and 67.6%, respectively. Among them, five GO terms were significantly enriched. The terms include defense response by callose deposition, regulation of transport, cellular response to the extracellular stimulus, vesicle organization, and ADP binding (Table S3). Moreover, 14 STs were mapped to 19 KEGG pathways, including anthocyanin biosynthesis, beta-alanine metabolism, biosynthesis of antibiotics, biosynthesis of secondary metabolites, carbon metabolism, cutin, suberine and wax biosynthesis, cyanoamino acid metabolism, lipoic acid metabolism, metabolic pathways, phenylpropanoid biosynthesis, plant-pathogen interaction, propanoate metabolism, protein processing in the endoplasmic reticulum, purine metabolism, pyrimidine metabolism, riboflavin metabolism, RNA polymerase I, starch and sucrose metabolism and valine, leucine, and isoleucine degradation (Table S4).

### Validation of ssRNA-seq experiments using quantitative real-time PCR (qPCR)

**Figure 3:**
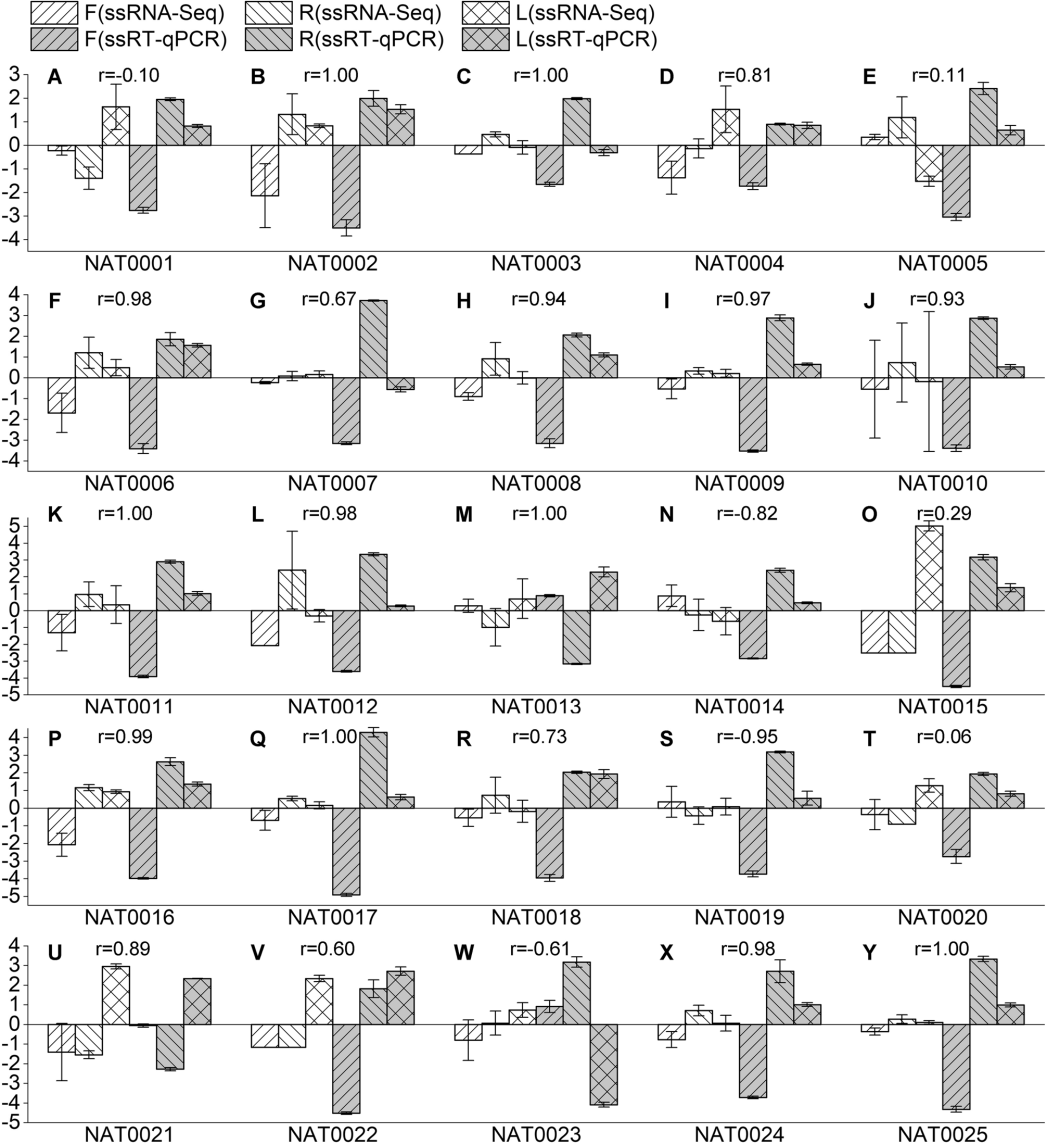
Validation of the expression level of 25 NATs in flower (F), root (R), and leaf (L) tissues of *S. miltiorrhiza*. The X-axis shows the expression levels in the three tissues using the two methods: ssRNA-seq and ssRT-qPCR. Y-axis shows the relative expression levels normalized to the mean expression levels across the three tissues. Error bars represent the standard errors. r: the correlation coefficient between the expression levels determined by the two experiments.

To validate the expression levels of *cis*-NATs and STs obtained from ssRNA-Seq in *S. miltiorrhiza*, we performed strand-specific reverse transcription-quantitative real-time PCR (ssRT-qPCR) on 25 SAT pairs with three replicates for each tissue. We selected 25 SAT pairs for validation if they met one of the following two conditions: (1) the expression profiles of NATs and parental genes were positively correlated with r values ≥ 0.9; and (2) the parental genes of these NATs were mapped to the GO terms or the KEGG pathways of interest. The SAT pairs satisfied condition 1 included SAT0005, SAT0010, SAT0011, SAT0013, SAT0015, SAT0018, SAT0021, SAT0024, SAT0025. The SAT pairs satisfied condition 2 included SAT0001, SAT0002, SAT0003, SAT0004, SAT0005, SAT0006, SAT0007, SAT0008, SAT0009, SAT0010, SAT0011, SAT0012, SAT0013, SAT0014, SAT0015, SAT0016, SAT0017, SAT0018, SAT0019, SAT0020, SAT0021, SAT0022, SAT0023. Detailed information for these 25 SAT pairs were shown in Table S5. The sequences of NATs and STs were showed in Sfile 2. For each transcript, we calculated the average expressed level of three replicates. Then we calculated the Pearson correlation coefficients (r) of the expressed level between ssRNA-Seq and ssRT-qPCR. Forty-one of 50 transcripts showed a positive correlation, twenty-three of which had an r valve more than 0.9 (Fig. 3, Figure S1). These results indicate that the data from RNA-Seq and qPCR are highly consistent. Moreover, the qPCR data also confirmed the differential expression of *cis*-NATs in the three tissues analyzed.

### Differential expression of cis-NATs in flowers, leaves, and roots

The expression of *cis*-NATs in flowers, leaves, and roots was analyzed using nine RNA-Seq data from the three tissues with three biological replications from each tissue. We found six *cis*-NATs differentially expressed among three tissues in *S. miltiorrhiza*, namely, NAT0005, NAT0039, NAT0050, NAT0076, NAT0080, NAT0131 (Table S6). Among them, NAT0076 in leaves was expressed higher than that in flowers. NAT0039 and NAT0131 in roots were expressed lower than those in flowers. NAT0080 in roots were expressed higher than those in flowers. NAT0039, NAT0076, and NAT0131 in roots were expressed lower than those in leaves. NAT0005, NAT0050, and NAT0080 in roots were expressed higher than those in leaves.

### Co-expression of NATs and cognate STs

It is shown that NATs could regulate the expression of corresponding STs^12^. To preliminarily explore the function of NATs in *S. miltiorrhiza*, we examined the correlation of expression levels between *cis*-NATs and the cognate STs using nine RNA-Seq data from the three tissues with three biological replications from each tissue (Table S7). A total of 19 of 168 (11.31%) *cis*-SATs pairs had a significantly positive correlation coefficient with r valve ≥ 0.9. And the *trans*-SATs pairs had a relatively low proportion. Only 37 of 644 (5.75%) pairs had a significantly positive correlation coefficient.

**Figure 4:**
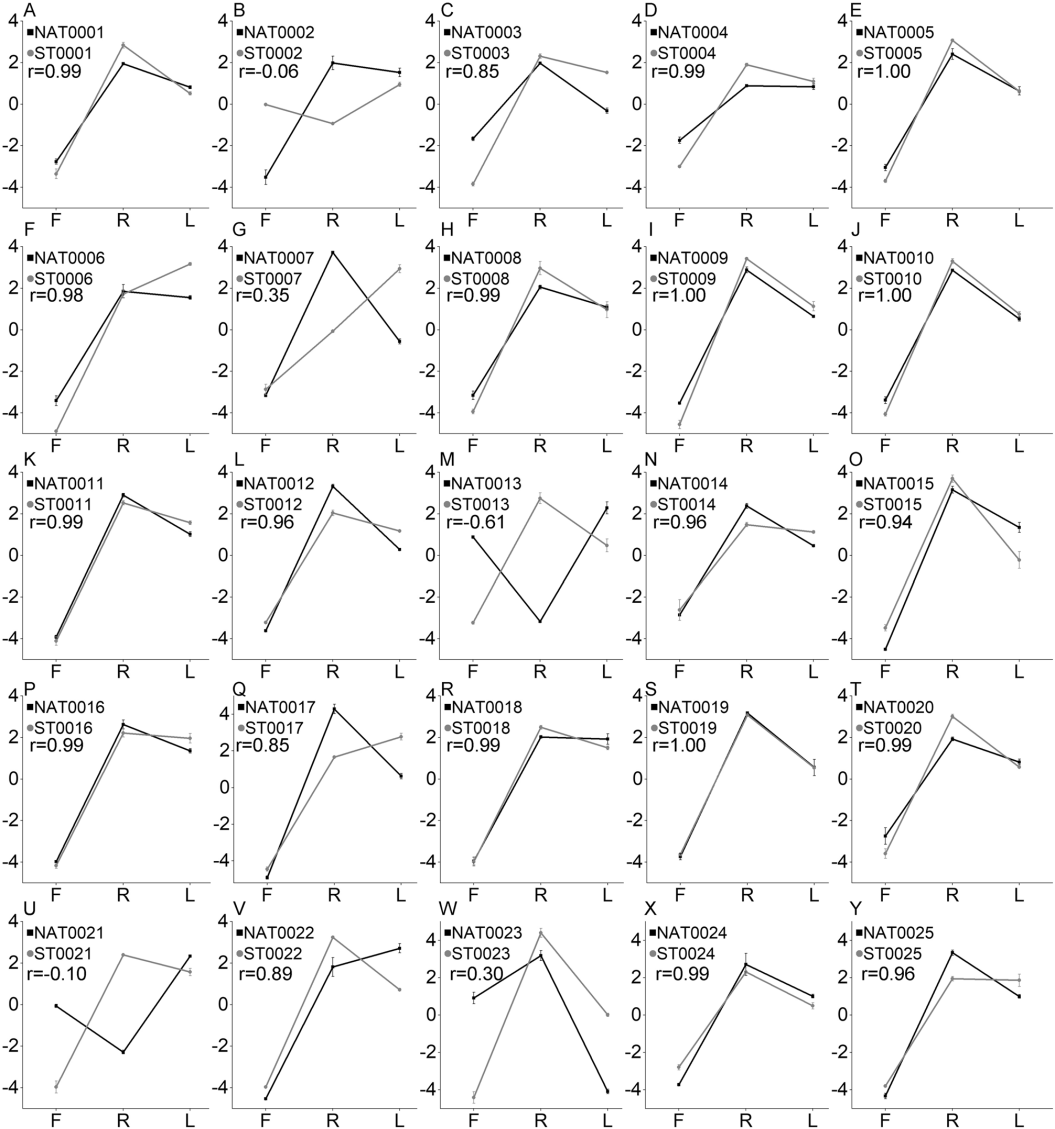
The correlation of expression levels of 25 NAT and ST pairs in flower (F), root (R), and leaf (L) tissues of *S. miltiorrhiza*. The X-axis shows the three tissues. Y-axis shows relative expression levels of NATs and STs analyzed using the ssRT-qPCR method. Error bars represent the standard error among three replicates. The values of r represent the correlation coefficient of expression levels between NATs and STs.

We also examined the correlation of expression levels between NATs and the cognate STs using the qPCR data; 22 of 25 pairs had a positive correlation coefficient, of which 17 pairs had r valve more than 0.9 (Fig. 4). The remaining three pairs had a negative correlation coefficient. The correlation coefficient values are  − 0.06,  − 0.61, and  − 0.10, respectively (Fig. 4B, M, U). The results suggest a high correlation between 68% NATs and their cognate STs.

### Differential expression and co-expression of NATs associated with tanshinone biosynthesis

**Figure 5:**
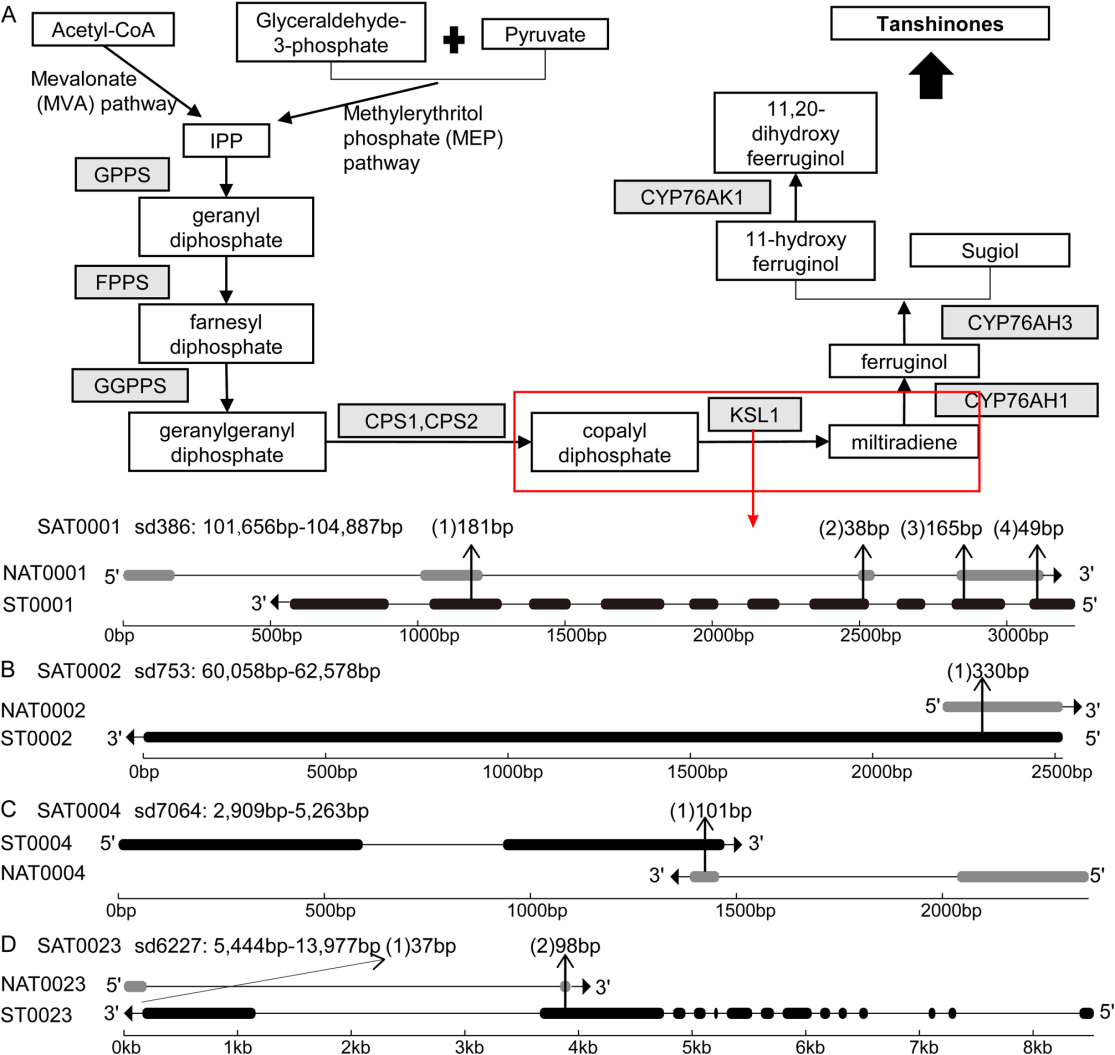
Gene structures of four SAT pairs. Panel (**A**) had two parts: the biosynthetic pathway of tanshinones in *S. miltiorrhiza* (above) and the gene structure of SAT0001 pairs (below). The enzymes were shaded in gray. The related ST and chemical compounds were highlighted with a white rectangle. Solid arrows indicated the reactions that have been experimentally validated, and dashed arrows indicated the hypothetical reactions. KSL1 that was encoded by ST0001 was marked by a red rectangle. In the area describing the gene structure, gray and black horizontal bars represent NAT and ST’s exons, respectively. The lines between bars represent introns. Arrows indicate the directions of transcription. The length of the overlapping region is marked above the structure. Similarly, panels (**B, C, D**) displayed the structures of SAT0002, SAT0004, and SAT0023 pairs in the same way as for SAT0001, respectively.

Tanshinones are a class of active compounds mainly produced in the roots of *S. miltiorrhiz*a. They are widely used for the treatment of cardiovascular diseases^20^. We found that the *SmKSL1* gene (Sfile 3) involved in tanshinone biosynthesis had a *cis*-NAT. SmKSL1 converses diterpenoid precursor, (E,E,E)-geranylgeranyl diphosphate (GGPP) to the skeleton of miltiradiene (Fig. 5A)^23^. This SAT pair was named SAT0001, which contains ST0001 and NAT0001. There were four overlapped regions with a length of 181 bp, 38 bp, 165 bp, and 49 bp, respectively (Fig. 5A). Both NAT0001 and ST0001 had the highest expression level in roots and the lowest in flowers (Fig. 4A). The correlation coefficient of this SAT pair was 0.99, indicating a co-expression.

### Differential expression and co-expression of NATs associated with glycosylation and de-glycosylation

Glycosylation is a key step in modifying the complexity and diversity of secondary metabolites in plants^36^. The uridine diphosphate-glucosyltransferase (UDP-glucosyltransferase, UGT) is one of the glycosyltransferases that use UDP-glucuronic acid as a glycosyl donor to catalyze glycosidic bond formation^37^. We found two transcripts (ST0002 and ST0004), which encode candidate UDP-glucosyltransferases, had *cis*-NATs (Sfiles 4, 5). Besides, ST0002 was mapped to the UDP-glucose flavonoid 3-O-glucosyltransferase 6 gene of *Fragaria x ananassa* that can catalyze quercetin to form the 3-O-glucosides^38^. SAT0002 belongs to the type ST containing NAT (Fig. 5B). Both NAT0002 and ST0002 had one exon and one overlapped region with a length of 330 bp. NAT0002 had the highest expression level in roots and the lowest in flowers. However, ST0002 had the highest expression level in leaves and the lowest in roots (Fig. 4B). The correlation coefficient of this SAT pair was  − 0.06. The type of SAT0004 was convergent (with 3′-ends overlapping) (Fig. 5C). Both NAT0004 and ST0004 had two exons and overlapped in the second exon with 101 bp length. Both NAT0004 and ST0004 had the highest expression levels in roots and the lowest in flowers (Fig. 4D). The correlation coefficient of this SAT pair was 0.99.

β-glucosidase catalyzes the hydrolysis of terminal, non-reducing β-D-glucosyl residues to release β-D-glucose and the corresponding free aglycone^39^. ST0023 was mapped to the β-glucosidase 40 gene of *A. thaliana* (Sfile 6). ST0023 had a *cis*-NAT, termed NAT0023. The type of SAT0023 was convergent (with 3`-ends overlapping) (Fig. 5D). There were two overlapped regions with a length of 37 bp and 98 bp, respectively. NAT0023 had the highest expression level in roots and the lowest in leaves, whereas ST0023 had the highest expression level in roots and the lowest in flowers. The correlation coefficient of this SAT pair was 0.30 (Fig. 4W).

### Identification of miRNA targeting STs for cleavage in *S. miltiorrhiza*

It was reported that NATs regulate the expression of their STs by increasing the stability of ST mRNA through RNA duplex formation. Precisely, NATs can mask specific sites leading to mRNA degradation, such as miRNA^40^. We found a high proportion of positive correlation between NATs and STs from ssRNA-seq and qPCR data. To explore putative mechanism of NATs in acting as a regulator in *S. miltiorrhiza*, we identified the STs having miRNA-binding sites in the region overlapped with NATs using psRNATarget^41^. A total of seven STs potentially to be cleaved were identified, namely, ST0003, ST0005, ST0009, ST0010, ST0018, ST0019, and ST0025 (Table 2). Besides, all the seven SAT pairs had a positive correlation with a correlation coefficient of more than 0.85 (Fig. 4C, E, I, J, R, S, Y).

**Table 2:** List of miRNAs targeting the overlapped regions of STs.

ST	MiRNA	Start	End	Aligned MiRNA Fragment	Alignment^a^	Aligned ST Fragment	Inhibition
ST0003	PC-3p-131493_9	211	230	AGGU-GAAGGAGCUCAAGAA		UGCUUGAGCUUCUUCCACCU	Cleavage
ST0005	mtr-miR2673a_L-1_1ss21AT	680	700	CUCUUCCUCUUCCUCUUCCUC		AAGGAAGAGGAAGCGGAAGCU	Cleavage
ST0009	bdi-miR156e-3p_1ss21GT	1204	1225	GCUCACUUCUCUCUCUGUCAUC		UAUGAGAGAGAGAGAGAUGAGA	Cleavage
ST0010	PC-5p-1795513_1	2018	2041	UCAAGGAGGGGAAAGACAAGGCCC		AGUUACUUUUUUUCUUCUUCUUGU	Cleavage
ST0018	pab-miR951	230	251	UGUUCUUGACGUCUGGACCACG		GUCGGUGGAGACGUCAAGAAAG	Cleavage
ST0019	stu-miR156d-3p_R-1_1ss10GT	1887	1907	GCUCUCUAUUCUUCUGUCAUC		GGUGAGAGGUGGAGAGAGAGC	Cleavage
ST0025	osa-miR171b	561	581	UGAUUGAGCCGUGCCAAUAUC		CAUUUUGGCAUGGCUAGAUUC	Cleavage

^a^“**:**”: match; “-”: mismatch.

### Identification of pri-miRNAs originating from NATs

**Figure 6:**
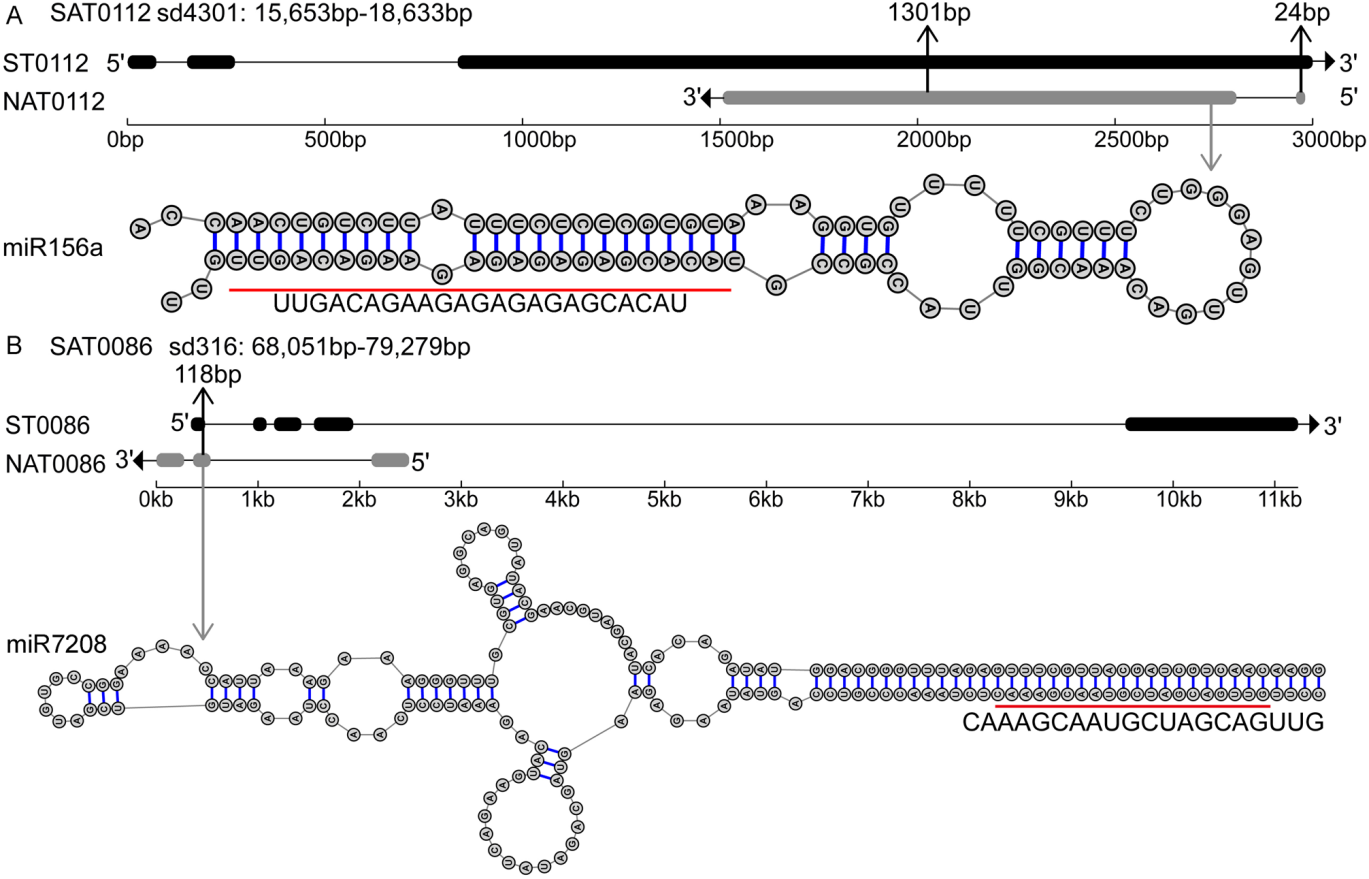
Gene structures of two SAT pairs and the predicted hairpin structures of pre-miRNA. (**A**) and (**B**) panels showed information about SAT0112 and SAT0086, respectively. Each panel had two parts, gene structures (above) and predicted hairpin structure (below). In gene structure, the gray and black bars represent exons of NAT and ST, respectively. The lines between bars represent introns. Arrows indicate the transcription direction. The length of the overlapping region was marked above the structure. In the part of the predicted hairpin structure, the miRNA was marked by a red line. The gene structures were drawn using GSDS v2.0^62^ and the hairpin structures of pre-miRNA were drawn using RNAstructure v6.0.1^63^.

MiRNAs can regulate gene expression by direct cleavaging complementary transcripts to regulate the metabolite biosynthesis in plants^42,43^. We searched the NATs against miRNA sequences found in *S. miltiorrhiza*^44,45^ to identify the pri-miRNAs originating from NATs. Two miRNAs, namely, miR156a and miR7208, were aligned to the NAT0112 and NAT0086, respectively (Fig. 6A, B). We then analyzed the secondary structure of the region aligned to miRNAs in NATs, showing the secondary structure meets the criteria of plant miRNA annotation^46^. The miR156a originated from the overlapped region of the SAT. In a previous study, miR156 could target SQUAMOSA promoter binding protein likes (SPLs) transcript in *S. miltiorrhiza*^47^. And the miR7208 did not originate from the SAT's overlapped region, and its function was unknown.

## Discussion

NATs have been identified in many plant species and are important regulators of various biological processes^12^. Understanding the regulatory mechanism of NATs is important for the quality improvement of *S. miltiorrhiza*. A total of 812 NATs, including 168 *cis*-NATs and 644 *trans*-NATs, were identified in *S. miltiorrhiza* in this study through genome-wide analysis.

The general features of NATs from *S. miltiorrhiza* are similar to those found in other plants. However, the number of identified NATs was lower. The following reasons may cause it. Firstly, we defined NATs as transcripts complementary to protein-coding genes, so we only retain the SATs whose STs are protein-coding genes. Secondly, some NATs are polyadenylated, and some are not. The ratio of these two types of NATs varies in different species^17,48^. In this study, we identified only the polyadenylated NATs. Thirdly, NATs usually have lower expression levels. For instance, NATs were more than ten folds lower than their STs on average in some organisms^49^. We might not identify the NATs with very low expression levels. As a result, it is possible that performing polyA (-) RNA-seq will reveal additional NATs in *S. miltiorrhiza*.

To predict the function of NATs identified in *S. miltiorrhiza*, we performed GO and KEGG pathway enrichment analyses on the STs with *cis*-NATs. These STs were mapped to a broad range of terms from biological processes, cellular components, and molecular functions. Some STs were mapped to multiple pathways. For example, ST0117 was mapped to four pathways: beta-alanine metabolism, carbon metabolism, propanoate metabolism, valine, leucine, and isoleucine degradation. ST0087 was mapped to three pathways: purine metabolism, pyrimidine metabolism, and RNA polymerase I.

In contrast, multiple STs were mapped to the same pathway. For example, six STs, namely, ST0137, ST0023, ST0135, ST0076, ST0141, and ST0002, were all mapped to the biosynthesis of secondary metabolites pathway. Two STs, namely, ST0023, ST0135, were mapped to the phenylpropanoid biosynthesis pathway. These results suggest that NATs may play a comprehensive role in the biological processes in *S. miltiorrhiza*.

Several NATs are found to associate with genes involved in important pathways. SmKSL1 is a key enzyme involved in the biosynthesis of tanshinones. It catalyzes diterpenoid precursor (E,E,E)-geranylgeranyl diphosphate (GGPP) to miltiradiene^23^. The *cis*-NAT (NAT0001) was identified to pair with *SmKSL1*. *SmKSL1* and NAT0001 showed a high level of co-expression with a correlation coefficient of 0.99. Both *SmKSL1* and NAT0001 were highly expressed in roots. The result was consistent with the accumulation of tanshinones in the roots of *S. miltiorrhiza*. As a result, NAT0001 might represent a novel mechanism in the regulation of tanshinone biosynthesis by NATs.

Besides, NAT0004 is a *cis*-NAT paired with ST0004, which encode a candidate UDP-glucosyltransferase catalyzing the transfer of sugars to various receptors to form the glycosidic bond^37^. Secondary metabolites, such as flavonoids, are important receptors of this reaction^36^. This enzymatic reaction leads to the glycosylation of metabolites and is a key step in the biosynthesis of various bioactive compounds^50^. Like *SmKSL1* and NAT0001, NAT0004 and ST0004 also showed a high co-expression pattern with a correlation coefficient of 0.99. As a result, NAT0004 might represent a novel mechanism in regulating flavonoids and glycosylation by NATs in *S. miltiorrhiza*.

NATs can regulate the expression of their STs at one or multiple steps of the gene expression process, such as pre-transcription, transcription, and post-transcription^51^. The regulation of gene expression by NATs can be either positive or negative^51^. We found 17 of the 25 SAT pairs had a significantly positive correlation. These NATs can positively regulate the expression of STs by the following two mechanisms. In the pre-transcription step, positive expression correlation may result from transcriptional activation. For example, NATs can enhance H3K4me3 deposition to their STs’ promoter, subsequently activate transcription initiation of STs^17^. In the post-transcription step, NATs can positively regulate the expression of their STs by limiting mRNA degradation. For example, NATs can mask specific sites that lead to mRNA degradation through binding with their STs, increasing the ST mRNA stability^16^.

In contrast, we found three pairs of ST and NAT had negative correlation coefficients. These NATs can negatively regulate the expression of their STs by the following mechanisms. In the pre-transcription step, NATs can regulate transcription initiation by affecting histone modifications^52^, DNA methylation^53^, and mediating chromatin modifications^54^. In the transcription step, NATs can negatively regulate their STs’ expression by co-transcription, resulting in transcriptional interference. For example, a DNA region is transcribed in both directions simultaneously. The two RNA polymerases on the opposite strand collide, causing transcription to pause^55^ simultaneously-transcription step, negative expression correlation may result from translational repression. For example, NAT can bind to the 5′ end of the NAT mRNA resulting in the binding site of the 30S ribosomal subunit, thereby inhibiting NAT translation^56^. The next step is to validate the function of NAT0001 and NAT0004 and explore the potential molecular mechanisms.

## Materials and methods

### Sample collection

Two-year-old *S. miltiorrhiza* (line 99-3) plants were grown under natural growth conditions in the garden of the Institute of Medicinal Plant Development, Beijing, China (Geospatial coordinates: N 40° 2′ 0" E 116° 16′ 5′).Flower (f), leaf (l) and root (r), samples were collected from three individual plants (named as p01, p02 and p03) and stored immediately in liquid nitrogen. We kept these samples in the  − 80° refrigerator until use.

### RNA extraction, library construction, and ssRNA sequencing

We divided these samples into two groups. In one group, the replicate samples were mixed by tissue type, named as p00_f, p00_l and p00_r respectively. In the other group, we extract RNAs from each of the samples (p01_f, p02_f, p03_f, p01_l, p02_l, p03_l, p01_r, p02_r. p03_r). We extracted RNAs from in total 12 RNA samples using the RNA extraction kit (TianGen, China). NanoDrop 2000 spectrophotometer (Thermo Scientific, USA) and Agilent 2100 Bioanalyzer (Agilent Technologies, USA) were used to detect the quantity and quality of total RNA, respectively. Then mRNA was isolated from total RNA using OligoTex mRNA midi kit (Qiagen) and cleaved into small fragments with 300 bp to 400 bp in length. The library construction was carried out using the ScriptSeq mRNA-Seq Library Preparation Kit (Illumina-compatible) according to the manufacturer’s recommendation. Sequencing was carried out using the Hiseq 2500 platform (Illumina, America). The raw sequence data have been submitted to GenBank under the Bioproject number: PRJNA683774.

### Prediction of NATs

To globally identify NATs in *S. miltiorrhiza*, we developed a stringent selection pipeline that contains three steps (Fig. 1).

In step 1, RNA-seq data of each tissue were mapped to the reference *S. miltiorrhiza* genome^25^ using TopHat in the Tuxedo packages (v2.1.1)^57^. Mapped reads were assembled into transcripts and then annotated based on the reference genome using Cufflinks in the Tuxedo packages (v2.2.1)^57^. We merged the reassembled transcripts from three tissues into a single unified transcriptome.

In step 2, we searched the reassembled transcripts against protein-coding transcripts based on the annotation of reference genome using the BLASTN (v2.7.1) with an E-value cutoff of 1e-5. The BLASTN results were further analyzed using the Perl script in NATpipe^58^. We consider the transcripts meeting one of the following two conditions as candidate NATs: (1) the complementary region is longer than 50% of either transcript of the NAT pair; (2) the consecutive complementary region of the NAT pair should be 100 bp or longer. We selected the transcripts that complement transcripts annotated as genes and located in the same loci with the gene as *cis*-NATs. By contrast, we considered the transcripts located in different loci with the gene as *trans*-NATs. Then, we predicted RNA hybridization for *trans*-NATs using the RNAplex (v2.4.14). The results from BLASTN and RNAplex analyses were evaluated using the Perl script in NATpipe^58^. The transcripts meet the following two conditions were considered *trans*-NATs: (1) annealed region coincident with the complementary region and is longer than 80% of the complementary region, (2) any bubble within the annealed region shorter than 10% of the total region.

In step 3, we removed the transcripts with a length shorter than 200 bp and those with the potential to encode proteins. We considered the transcripts meet one of the following three conditions to have the protein-encoding potential: (1) transcripts with the maximum length of coding protein more than 100 aa, (2) transcripts matching the Swissprot or Pfam database, and (3) transcripts with Coding Potential Calculator (CPC) score more than 0^59^. The transcripts with the length of amino acid greater than 100 that is the length of the open reading frame (ORF) greater than 300, were identified using the getorf program in EMBOSS package (v 6.5.7.0) with the following parameter: “-minsize 300 -find 1 -reverse N”. We searched these ORFs against the Swissprot database using the BLASTN (v2.7.1) with an e-value cutoff of 1e-5. Next, we searched the ORFs against the Pfam database using the PfamScan.pl (v1.6) with an e-value cutoff of 1e-5.

### Conservation of NATs identified in *S. miltiorrhiza*

To determine the conservation of NATs identified in *S. miltiorrhiza*, we searched the PlncRNADB (http://bis.zju.edu.cn/PlncRNADB/index.php) and GreeNC^34^ databases for homologs using BLASTN with the following parameters: a word size 7, an e value cutoff of 10^–5^, and 50% alignment coverage of both sequences.

### Functional enrichment analysis of STs

The sequences of STs having *cis*-NATs were extracted using the gffread module in Tuxedo packages (v2.1.1)^57^ and then annotated on the Metascape web server^60^. We further analyzed the results related to the GO and KEGG in detail^35^. We consider the terms with a *q* value < 0.05, a minimum count of 3, and an enrichment factor ≥ 1.5 as significantly enriched. The *q* value is the multi-test adjusted *p* value calculated using the Banjamini–Hochberg method. The enrichment factor is the ratio between the observed counts and the counts expected by chance.

### Differential expression analysis of NATs

We analyzed the expression of NATs using the Cuffdiff in the Tuxedo packages (v2.2.1)^57^. The NATs were considered differentially expressed under the two conditions: $\left| {{\text{log2}}\left( {\text{fold change}} \right)} \right| \, \ge {\text{ 1 and q }} \le \, 0.0{5}.$

### Strand-specific quantitative real-time PCR analysis

To validate the expression of NATs and their cognate STs in each tissue, we collected samples from root, stem, and leaf and extracted RNA using the RNA extraction kit (TianGen, China). We used the strand-specific quantitative real-time PCR (ssRT-qPCR) method^61^ to validate the expression level. The gene *SmActin* was used as internal control, as described previously^29^. First, the sequences of NATs or STs were extracted using the gffread module in Tuxedo packages (v2.1.1)^57^. Then, the primers were designed on the Integrated DNA Technologies (IDT) webserver. Nucleotide tags were added to the primers for reverse transcription to distinguish NATs and STs. The tag sequence for NATs was “GCTAGCTTCAGCTAGGCATC,” and that for STs was “CCAGATCGTTCGAGTCGT.” The primer sequences were listed in Table S8, Sfile 1. RNA was reversely transcribed to cDNA using strand-specific primers and the TransScript kit (TianGen, China). We carried qPCR with three technical replicates for each cDNA using the qPCR kit (Takara, Japan). We used approximately 1 µl cDNA, 1ul 10 µM each of the forward and reverse primer, 0.4 µl passive reference dye (50×) polymerase, 10 µl 2 × TransStart Top Green qPCR SuperMix and 6.6 µl ddH_2_O for qPCR with the following conditions: 94 °C for 30 s; 35 cycles of 94 °C for 5 s, and 60 °C for 34 s.

We used the average values from three technical replicates to calculate the Pearson correlation coefficients of expression level between ssRNA-Seq and ssRT-qPCR. The false discovery rate (*q* value) was calculated using R’s fdrtool package (v3.60).

### Correlation of the expression profiles of NATs and their cognate STs

For ssRNA-Seq data, we calculated the Pearson correlation coefficients of expression level between NATs and their cognate STs based on each transcript’s FPKM values. For ssRT-qPCR data, we calculated the Pearson correlation coefficients based on the values of Ct[X] − Ct[ACT], in which ‘ACT’ stands for Sm*Actin* gene and ‘X’ stands for transcripts tested in this study. The false discovery rate (*q* value) was calculated using R’s fdrtool package (v3.60).

## Supplementary Information

Supplementary Tables.

Supplementary Information.

Supplementary Information.

Supplementary Information.

Supplementary Information.

Supplementary Information.

Supplementary Information.
