# Supplementary material for: Genome-wide identification and functional characterization of natural antisense transcripts in Salvia miltiorrhiza
Source: Sci Rep. 2021 Feb 26;11:4769. doi: 10.1038/s41598-021-83520-6 (PMC7910453; doi:10.1038/s41598-021-83520-6)
Supplement: Supplementary file 2 — Supplementary Information. [file 41598_2021_83520_MOESM2_ESM.docx]

>NAT0001

GGTCCGGTTCACCACCGACGCCGCCGCTCCCACTCCTACAGCCGCTAATTTGATGTGAGCAACAGCCACCATTTAAACGATGACGTTCTATTGGCCTCGTTTTCTTATGCTTCGCCGTCATCCTCAACGACACCGGCGGTGTTGGTGCCCTGCGCTTTGCTTTGACCGCAGAAGTGTTGCACTTGTATTCTTGGAAGAATCTTACCTCAAAAGTTTGCACATCGTTGAGCAGCCGGTCAACCATGGAGACATGCCTACACAGATCAATGCACTCTTCACTCAGAAGCATTTCATCGGATAGTTTTATACCGATGAATTGCATCGACATGAGGATGCAGATTCTGCAGCCAATTGACACCCAGGAGGCAGACAAGTAATCATCCAAGGTCAACATGCTCTTGGTCAGCATACGGAGCCAGTTCCTCTAAACAACGACGAAAAAGAATAGTGTCGATTTCAGATCGGAAGTATCGGTCGACTCCCAACCTTTGGAGGGTGTCGATTATACAGAGATTAGAGGGCATATCCGCCGCAGGCTGAACAGCCGCAGCTGCCGGAAAGTTACTGTCTTCCCCCTTGAACTTCTCTGCTATCTTCCCCATCAAATCCTGCGATAAGGGCATTCATTCAAATCGAGTATATTTCATCCGGTGTAGCATCGATAACAAGATTTGGAGGTATCATACTTACTGTTGTAGTAAGGTTGCATTTCACGGCGAAAGATGACTTATTGGTGATC

>ST0001

AACTTGTATACAATATCGTCGCCTCTCGTAAAATACTATATTCACTACATGACAGAAAATAATCATATAATAACGCAAAAAATACATTATATCCGTTATTATATATGATCATGAATTTGAATGCATCATTGCAGTAGCATATTTCAGTCAACCTTTTGTTGTCATCTCTTACAAGAATCCCTCCTCATTTTCAGATACTCCATTCATACATATCCAGCAACATAAACAGAGCCTGGAATCACCGCCGCCGCCCCAGAGCTAAAGATGTCGCTCGCCTTCAACCCGGCAGCCACCGCTTTCTCCGGCAACGGGGCTCGGAGCAGGAGAGAAAATTTTCCGGTGAAGCATGTTACAGTCCGAGGATTTCCGATGATCACCAATAAGTCATCTTTCGCCGTGAAATGCAACCTTACTACAACAGATTTGATGGGGAAGATAGCAGAGAAGTTCAAGGGGGAAGACAGTAACTTTCCGGCAGCTGCGGCTGTTCAGCCTGCGGCGGATATGCCCTCTAATCTCTGTATAATCGACACCCTCCAAAGGTTGGGAGTCGACCGATACTTCCGATCTGAAATCGACACTATTCTAGAGGACACATACAGGTTATGGCAACGGAAAGAGAGAGCGATATTTTCGGATACTACTATTCATGCAATGGCATTTAGACTTTTGCGTGTCAAGGGATATGAAGTTTCATCAGAGGAACTGGCTCCGTATGCTGACCAAGAGCATGTTGACCTGCAAACGATTGAAGTGGCGACAGTTATCGAGCTTTACAGAGCAGCACAGGAGAGAACAGGGGAAGACGAGAGCAGTCTTAAGAAACTACATGCTTGGACCACCACCTTTCTTAAGCAGAAGTTGCTCACTAACTCCATTCCTGACAAGAAGTTGCACAAACTGGTGGAATACTACTTGAAGAACTATCACGGGATATTAGATAGAATGGGAGTTAGACAAAACCTCGACCTATATGATATAAGCTATTATCGAACTGCAAAAGCTGCAAATAGGTTCTCTAATCTATGCAGTGAAGATTTTCTAGCATTTGCGAGGCAAGATTTTAACATTTGCCAAGCCCAACACCAGAAAGAACTTCAGCAACTGCAAAGGTGGTATGCAGATTGTAAGTTGGACACCTTGAAGTATGGAAGAGATGTAGTGCGTGTTGCTAATTTTCTAACTTCAGCAATTATTGGTGATCCTGAATTGTCTGATGTTCGCATAGTCTTTGCCCAACATATTGTGCTTGTGACACGTATTGATGATTTTTTCGATCATCGTGGGTCTAGAGAAGAGTCCTACAAGATCCTTGAATTAATAAAAGAGGCTCATGTCGAACACGGACGTAGTGTTAAAGATTTTCTAATAAAGCTGTGGGTTCAAATACTATCAATTTTCAAGAGAGAATTGGATACATGGAGCGATGACACGGCACTAACCTTGGATGATTACTTGTCTGCCTCCTGGGTGTCAATTGGCTGCAGAATCTGCATCCTCATGTCGATGCAATTCATCGGTATAAAACTATCCGATGAAATGCTTCTGAGTGAAGAGTGCATTGATCTGTGTAGGCATGTCTCCATGGTTGACCGGCTGCTCAACGATGTGCAAACTTTTGAGAAGGAACGCAAAGAAAATACTGGAAACAGTGTGACCCTTCTGCTAGCAGCTAACAAAGATGACAGCTCCTTTACTGAAGAAGAAGCCATTAGAATAGCAAAAGAAATGGCTGAATGTAACAGGAGACAACTGATGCAGATTGTCTACAAAACAGGAACCATCTTCCCAAGACAATGCAAAGATATGTTTCTGAAGGTATGCAGGATTGGGTGTTATTTGTATGCAAGCGGCGATGAATTCACATCTCCACAACAAATGATGGAAGATATGAAATCCTTGGTTTATGAACCCCTAACAATTCATCCTCTTGTAGCTAATAATGTGAGAGGGGAATGAAATGAATTCTGTGAGCAACTATTTAGGGCAGATCATGTACTCTTGTAAAAGTATACTGTCTCAAAATCATTAATGCCACAATAAGATGATACATTGATTTCAGCAGTCTTCAAGCATTTTCACTTACTGGCATGAGTGGCATCCTATACTATGATTAGGAAACAAATCACTGATGAGAAAAGAAATGACATTCTTAGCTTAGAACCTCACCCTGTGTCTCAACTCAATGATAGCTTAGATATATACAAGTGATATAGATTAATCCACCACACATTTAGATCTTGTTTAATCATCTAATCCTTCAAACATTCAATGCAATGTATTATCAGTCGAATCTATTGTACGACCTAAAATGACTGCAAAATATACCTTCTGCGGTGA

> NAT0002

CGCTGTCTTCACCGCCGTGACCAGGTGGCTCACGACGGGGAAGGGGATGAACACTAGGCTTGCTTTTTCATCAACAGCCATTTTTATTTTATTTAATCACACTGTATCTTAAGTCTTGCAATGCGTTTATATATATAAGTAAAGAAAACATGTGATATGCAACTTTGTTGTCAATGAAACTTGTTGGTAGTAAAATTGTTGGTCACCAAAAATGTTTGGCTACCTTAAAGTGTATATATAACTATCAATAAAGTAATTAACACCAAAAATTGTGGTTAAATAAGATTCCCCTAATAATTAAGTATGAGATTTAGAGTGATATATAATAAA

> ST0002

ATGGCTGTTGATGAAAAAGCAAGCCTAGTGTTCATCCCCTTCCCCGTCGTGAGCCACCTGGTCACGGCGGTGAAGACAGCGGAGCTCCTCGCCTCCCACGACAGCCGCCTCTCCATCACAGTCCTCGTCATGAGCATGCCGACGGACACCAAGATCAGCTCCTACATCAAGAATCCGCGGATCAACTTCGTGCAGCTCGAACAAGACGTATCCAATGGCGCAGAAGCGATCATGAAGCCTCCCAAGAGCATGATGCATTTCGCCGGCCGCCACAGGGATTCCGCCCGGGCGGTGGTGTCTGAGATGAAGAGATCATGCAGAGTCGCTGGAATCTTCGTCGACATCATGTGTGTCGACATGATCGACGTGGCGAAGGAGCTCAAGATCTCGAGTTACATTTTCTTTGCTAGTGGCGCCGCGGTTCTAGGGCTCACGTTCGATTTGCAGAGTCTCCGAGACGATGGTGGCCGGAATTTGGCGGAGTTCGAGGGTTCGGATGAGGTGGTTTCGATCTCGTCGTATGTTAACCCGGTTCCGGCTAGGGTTTGGCCGGAATCGGTGTTCGACGGGGAGAGCGGCTTCCTCGAGCTATCGAGGAAGGCGCGAGAAGCGGACGGGATCGTCATCAACACTTTTCTTGAATTGGAATCATATGCGATAGGCTCAACCTATGCTAATGAGCGGATCCCGAGGTTTTACCCGATCGGGCCGATCATCGGTGAAGGGAAAGACGAGAATGATGAGAGCCGACAGAGGCGCGGGGAAATCATGCGGTGGCTAGACGGGCAGCCGGACTCGTCGGTCGTGTTCCTCTGCTTTGGGAGCATGGGAGCTTTCGGGGAGGAGCAGGTGGTGGATATCGCGGAGGCGCTGGAGCGGAGTGGGAAGCGGTTCTTGTGGTCGTTGAGAAAGCCGATTTTCGAAGGGGGATTTGCCTATCCGACGGAGTACGAGAATCCCGGGGAAGTATTGCCGGTAGGGTTCCTAGAGCGCACGGCAGGGGTGGGTAAGGTGATTGGGTGGGCCCCTCAGGTAGCGGTGTTGTCTCACCCTTCGGTAGGAGGGTTCGTGTCGCACTGCGGATGGAACTCGACTTTAGAGAGTGTCTGTTGTGGGGTCCCCATGGCCGCCTGGCCGTTGGGGGCCGAGCAGCAGACCAACGCATTCCAATTAGTGAAGGACATTGGAATTGCTGTCGAGATTAAGATGGATTATCGAAAGAACAGTGGTGAGATTGTGCCCAAAAGTATTATCGAGAAGGCAATCAAGCAGCTAATGGACCCCACGAATGAGATTCGAGTTAGGGTTAAGGAGTTGAAGGAGAAGAGCACGAGGGCCCTAATGGAAGGTGGGTCATCATATAATCACTTAGGTCTTCTAATTCACAATTTCTTCAAGAGTTGTTGA

> NAT0003

ATGTTTAATCCAGTATACAGTGAGCTTCTGATCCACGTTTCTCGACTTCTGCTCACAGGTGGAAGAAGCTCAAGCAGCATGGACTGCAGTCAAAAAACTGATGGTGCAAAGCATACCTTCCCATTACTGAGAAAACAAAAGTTTACAGATTGGTGAAGGACCAATTCTGGTTTTGCTTTCAAACCACATCACAGAGA

> ST0003

ATGGATGTGGTGAAACTGTTTAGCTTACTTCTCTTGTTCAGATTTGCATTAGCTAATGGAGATGCATCTAATAGAAAGCATTATGTAGTTTACATGGGGGAGCACTCATACAAGGACTCAGAATCAGTAATCACAGCTAACCATGAGATGCTTACCTCAGTAATGGGAAGGTATGCTTTGCACCATCAGTTTTTTGACTGCAGTCCATGCTGCTTGAGCTTCTTCCACCTTCATGAGGGGGCACAGGGTGCAGCGGTCCACCATTATACGAAAACCTTCAGAGGTTTCTCTGCAATGCTCACCTTGGATCAAGCAACAGAACTCCAAGAGAGTGATTCAGTAGTATCTGTGTTTGAGAGCAAGACAAACCACATCCACACAACACACTCGTGGGATTTTTTGGGTATAAGCAACTTGGAAAAAACCAATCAAGCATTGATGGATTCAACATCTGACACGATCATTGGAGTTATTGATTCGGGAGTATGGCCCGAATCTAAGAGCTTCAATGATTACGGGCTTGGTCCCGTGCCCGCCAAATTCAAGGGTGTATGCTCAACTGGTGAAAACTTCACTCTGTCAAACTGCAACAGAAAAATAGTAGGAGCCAGATTTTACTATAAAGGATTCATAGCAGAATCTGGGCCTCTTGAGTCATTCAACGAGACATTTTTCCTCTCAGCCAGAGATGCTGATGGTCACGGAACCCACACTGCATCCACTATTGCTGGCTCAGTGGTTCCCGACGTTAGCTTGTACGGAATAGGGAAAGGCACTGCAAGAGGAGGTGCACCAGGATCTCGACTGGCAATCTATAAGGCTTGTTGGTTTGACTTCTGTAGTGATGCAGACATCCTTTCTGCCATAGATGATGCAGTTAGTGATGGTGTGGATGTCATATCAATGTCCCTTGGACCAGATCCACCCCAGCCTATCTACTTCAGTGATGCAATCTCAATTGGAAGTTTTCACGCATTCCAGAAAGGAGTAGTCGTTTCTGCATCAGCAGGCAACAGCTTTCTCCCCGGAACTGTAGCGAACGTTGCTCCTTGGATTCTCACTGTTGCAGCAAGCACCATGGATCGTGAGATTCAAACTAATATATATCTAGGAAATTCACAAATTATAAAGGGTTTTAGTATAAACCCATTTCAGATGAACAACTTTTACAGTTTAGTGGCCGGAAGTGCAGCAGCAGCCCTCGGGGTTTCCTCTGCAAACGCAAGCTTTTGTAAGAGCAACACCCTAGATCCAGCTCTGGTTAAGGGAAAGATTGTTGTCTGCACACTTGAAATAATTCTTGATCACCGAAATGAAAAGGCTGCTGCTGTAAAACAAGCTGGCGGTGTGGGGATAATACTTGTTGATCCACTAGCCAAAGACATCGGGTTTCAGTTTGAAATACCGGGCGCATTAATCAGCATTCAGGAAGCAGAAAAGCTTCAAGCACACATGGTTTCACAACAAAACCCGGTGGCTAGAATATCCCGGACAATGGCGGTTCTGCCGACTAAACCAGCACCTGAAATGGCAATGTTCTCATCTGCGGGTCCTAATGTCATCAGCCCGGATTTAATTAAACCGGATATAACAGCACCAGGCGTGAACGTCCTGGCAGCATGGTCTCCATCGGCTACTGCTAACACTGCTGGAAGATCAATCGACTACAACATAATCTCGGGCACTTCAATGTCCTGCCCACATGTTTCTGCTATAGCTGCTATAGTCAAATCAGTCCATCCTTCATGGAGCCCTGCAGCGATCAAGTCTGCGATAATGACAACAGCAACAACTCAAGATAACGTTAGGAGCTTCATTAGAAGACATCCAAATGGAACTCAAACCTCACCTTTCGATTATGGATCCGGGCATGTCAATCCAGCTGCAGCAGTTGATCCCGGATTAGTTTATGATTTTGATAGCAGCGACATCATTGGTTTTCTGTGCAGCACTGGTGCAAGCCCTGCTCAGCTGAAAAACCTCACCGGTGAGACCACATATTGCAGAAACACCAAAACATCCTCATACGATTTCAATTATCCATCAATTGGAATCTCGAACCTGAAAGGAAATATATCAGTGCATAGGACAGTTACGTATTATGGGGAAGGGCCTGCTGTTTACAAGGCAGAAGTCGACTCTCCAGCTGGTGTATATGTCTCAGTCACTCCTAACAAACTCAGCTTCAAAAAGATCGGGGAAAAGATGTCTTTCATGATAATTTTCACACCCTATAAAACCAGTGAAGAGAACTTTGTGTTTGGAGCTTTAACATGGACTGATGGAATGCATGTCGTCAGGAGCCCGATTGGTCTTAACATACTCTCTCTAAGAAGTGTGTGA

> NAT0004

TGGTATTTTGAATAGAGGATCTCTCTTGGTAGTGGCCGATTGTTTGGTAAAAAGTGGTAGTATATTTTATGTTTCATCTTTCAAAAAGGCTATATCCCATTTTCACCCTAAAAAATAACATTGCTCGAAAACGCCGCGTTTAATGAGAACCATAATTAGATCGTTTTGGGGAGGATTGAGGAGAAGCGAAATATAAACAATCGAAGATCCGAAGTAATGATCGTGGGTTTCGAGGTTTCAAGCTCAATTGAGGTCATTTGCCATAACAGATGAATTGTGAATTCGTTAATTTGGTTATCTACTTCTATAATTTTATGCAGATATCAACGGTGGTGAGCTTGGAGCTGTTGATTTCCTTCTCCCTCTGTATCTCACTGATGAGGTTGCTTAAC

> ST0004

ATGGAAAACAGAAATATGGCTCATATTTCTTCTTCTTCCAGCTCTAAACCTAACATAGTTGTGTTTCCTTTCATGTCAAAGGGCCACACCATTCCTCTCCTCCACCTAGCACAGCTCCTCCTCGACCGCGGCCTTGCCACCGTAACCATCTTCACCACCCCTGCAAACCATCCATTCATATCTCAATCTCTCGCCGGAGCTCATGTTTCCATTGTCGACCTGCCATTCCCTCGCAACATCCAAGGTGTCCCACCGGGAGTCGAGAGCACAGACAAGCTCCCATCCATGGCTCTCTTTATACCATTCGTTCGAGGCCTGGAGCTCATGCAGCCTGCATTCGAGCAAGAGCTAGAGAAGATCCATTCACAAGTCACCTGCATCATATCAGATGGCTTCCTCCCATGGACCCTCGAATCAGCATCGAGGTTTGGCATACCGCGTCTTTCTTACTACGGCATGAGCTACTATTCCATGGCAGTGAGCCGCGATGCAGCCTTCAGCGGTCTCATGTCTTCACCTGAAACAGATGATGAGCCCTTCACGGTCAAAAGCTTTCCTTGGATTCAGATAACAAGGAATGATTTTGATGAGCCGGGCAGAGGTGAGCTTCTTATGGGTGGTAAAGAAGAGCGACTGGAACTACTAAGCGACGACTTTCAATCTACAGTAAGTGAAAGAGGTATCATAGTAACAGATTGGGTTGATCAAGAAGAGATTCTTGAGCATCCAATTGTACAGGGCTTCTTGAGTCACTGTGGCTGGAACTCGGTCTTGGACGGAATATGTGCAGGAGTTCCCATTCTGGCGTGGCCGATGATGGCCGAGCAAGGTCTCAACGCAAAGCTGGTAGTCGAAGAGATAAAAGTTGGTTTAAGGATTGGTACAGTCGATGGAAGATCAAAAGGATTCGTCACTGCAAACAACTTGAAGAGCGCAGTGAGAGAGCTAATGGGCAGTGGAAAGGGAAAACAGTTAAGGGAGAGGGTGAAGGAGATTGCTAAAGCCGCCATAGAAGCTACGAGCGAAGGTGGCTCTTCATGGAATGCGTTAAGCAACCTCATCAGTGAGATACAGAGGGAGAAGGAAATCAACAGCTCCAAGCTCACCACCGTTGATATCTGCACCAAATAA

> NAT0005

GAGACCTACGCGCTGATTCACGATTAGACAGCTTCCGCTTCCTCTTCCTTTCATCACAATCAGAGCCCGGGCTCGTTGGCAATTGTTTTGAAGCCATTCCTCAAATATTATCGCAGTAATGATCGAGCGATTGATTATTTACAATTAAATAATGTTTACAAAACCCTACAGCAGATTTGACTCGACCTGATCGGCTAATATTGGGGATCGAATGATTAAAGAAAGCAAGAAAAAAATGATACAGCAGAACAAAATTAGGGCTTAGCGTGCAAATTCATAGAACCAGTAAAGGAAAACTACCGAGAACGAGTGAAGAATTCTAATCTTATGCATCAGCATCGCCAATGTCAATAAAAATTAGAGATTAAAACCTCATAACCTTATCGAAAACCACTCAAATTCCGGAAACGCAAACAAAAAAGTCTACAGTATCTCGAGAAAAACAAATCAGACAGCTCCGATCAGAAACCAATCTGCAAAT

> ST0005

ATGGCTTCAAAACAATTGCCAACGAGCCCGGGCTCTGATTGTGATGAAAGGAAGAGGAAGCGGAAGCTGTCTAATCGTGAATCAGCGCGTAGGTCTCGGATGAAGAAGCAGCAACGCTTGGATGAGCTGATTGGAGAGGAGGGCCGGATAAAGGAGGAGAACAAGAAGCTGAGTCAGATGATTGATGCTACTGCTCAGCTGCACTTAAACGTTGCGTCCAACAATAATGTGCTGAGGGCTCAGATTGCTGAACTCACTGACCGCCTCAGGTCGCTCAATTCTGTACTTCAGATTGCCTCCGAGGTGAGCGGCCTTGTGATTGATATTCCAGACATCCCGGATGCCTTGCTTGAGCCGTGGCAGCTGCCTTGCCCGATTCAGTCTGCCCCTGCTTCTGTTGATATGTTCCAATGCTGA

> NAT0006

GTTAGTAATATTTGAGTAAAAAGGAGAACATTTTTTACAATGGAAGCATGATTTTACTTACTCAAGGACTCGTTTCCAGTAATAGTTGCTTGCTTCATCATCCATTAGAATATCTTCTAAGGCTTCAGAAGTAGTTGGAGGGTTGGAAAATTCAGCCAGGCTGATGAAATTGTCAGGTTCTTGATCTGTGCTTAAACACACCGGAGATGCTGAGATGAGATCAGACAGGTCGAAGTGCATCTTTGAAGCTCGGTTGGCATGTCAGAGCGTCAAACTCAGGTTTCTGAGTGAATCTTTCCAACTGCAATC

> ST0006

ATGGAAATGACGACAGAAGAAAATGGCTTAAAGAAAGGGCCGTGGACACCTGAAGAAGACCGGATGCTCGTTGACTGCATTCAGAAACACGGCCATGGCAGCTGGAAAGAGCTGCCAAAGCTTGCGGGGCTCAATAGGTGCGGGAAGAGCTGCCGCCTGCGTTGGACAAATTATCTCCGCCCCGATATCAAGAGAGGAGCCTTTACTGACGAGGAAGAAAGGGTCATTATCGATCTTCATGCAGAGCTAGGCAACAAGTGGTCGGTCATAGCTAGTTACCTGCCAGGGAGAACCGATAACGAGATCAAGAACTTCTGGAATACTCGTCTGAAGAAAAAGCTGCTTCGGAATGGGATAGACCCCGATACTCATATGCCAAGAACTCATCTTGATACCTTGTTACCTGTATTACCTCAGCTTCTCAGCTATAATAGTTCAAGAAATCTTACCTGGGAAGGTATTGTGAGATCTCAAGAGGATGCTGCTCGTCTCGCAAAAGTTCTACTGGTAGGTAACATTTTACAGTTGTTGAACACTGGTACTACTTCAGTTTTGCCTCCTGTCGCGATGGAAACTTTTAGTCCTAGCCTGGGATCAGCTCAAGTCACAAACAGACCTCCATGGGATTCCCATATGATGGGATTGCAGTTGGAAAGATTCACTCAGAAACCTGAGTTTGACGCTCTGACATGCCAACCGAGCTTCAAAGATGCACTTCGACCTGTCTTCCAGAACACAATGGAAACCAACAATGTGATGGATATAAATAGCAACAAATTTGGGGAATCATCTAACATGACACAGATTATGAACCTGGCTGAATTTTCCAACCCTCCAACTACTTCTGAAGCCTTAGAAGATATTCTAATGGATGATGAAGCAAGCAACTATTACTGGAAACGAGTCCTTGATCAAGCCAGACTGTCGCCATGATCAATCTCGTATCTGGACAGAGGCTGCTAACAGATGGTGTCAAGAGATAAATCACATCTCAAGGAATATACTTTAACATTTAGCACTGAGAAAATTTCCAGATTTCATGTACTACTAATTTCTTTGCGGTGGTATATAGACTCCCCAGCCTGTATGATCATCAGCAGTGTGATTCATGTTGTCCACAAGTTTTTCCCCCATTTTTTCCATGTATAAGTGCGAAATAAGTTCTACTTTCTTATCATGTAATAATCCATTTCCAAACTCACAGTAGGACACATGACTGCCACAGAGCCTATGTAAACAGAAATTATTCAGGCTGGTTTATGTTGTACGATAATCAGTCTCTCCCTGATTTTGAATTTTTCTCGCCTGGTTCATTCTGATTTTTGGCTCACCGTAACAGCTTCTATGAGAAGTAACACGAAGAAGTCCTACTATTAGGTAAAAGGAAATTGTTCATAAAAAAAGGGTAGAACCAAAATGTCAAATCACCTTATGTGGAGTGTCACAATCCAGGCATTCATGGGTCTTGGTCTGGGTAAAATCTACCTAGTTAAGAATAGTATTTTAGGATTGGATAAGAGTATTAGGTTATGACTTATGTGAGCTTTAATCTGCTAAAGAAGTCAATATGCAAGCAATATCTAAATAGTTAGGTAGGGTAGAAATCACATAGATACATAAAGAGAAACTTTTTAACAGATATACAATCTTGATATAAACTATTATTCTAATTTGGCTAGAAATAACATAACAGAGATAAGGATAGTCAACACAAGAATCCATAATTTCAAATGCCTGTTCCTTCCATAGATATCCGAAAATAACAATATGTGATATAAGCGTAGGTTCTTACCTGATACAAAAGCAATTGGAGTATAAGATTTCACAATGTCCCAAGTAAGCAGCTAACCCTGTGTAACAAGCGATGATCTCATAAACAAACTAAACCACCTACCTTTACCAATATTTTGATAAACTGCACCTATCTCATCAACCTTTAGAACATATAAGAAGAAGAGTATACATGATACTTGCACTCAGAACAAACTTTTCACTTAATCACCGAGAAAGTAAAAAAAAAAAAGGAAA

> NAT0007

AGTTCATGAGTCGTAGATATTTTCTTCTCTCTCTTCATCTTTTTGTGTCTCTCTGGCCACAACATTACACTTAATTCACTTCTGCCTAAAGCCTTCTTCACCTCGACACTTCTCCTTTATCGGGAAACTGCGATTACGCATCGAGATCTTCATATCTCATCGCTCTTCCTCTAAATTCACATGTAAAACATTGTTGCCTTTGTTCATTAGGTGCTGAAAGAATCTCCAAGACGCAGTTCTTCGTGCGATTCCTTGCTTGATACCAATCCTGTCATATTACCAAGGGATATATAATTATTGAAGCTATGGCGACTGTCTCCACTATACATAAGATTTTGGAGGTCTCTGATGTTCAATCCAGCAAGTTCTTCTCCCAAAAGCTTCCTGCAACCGCAACAGAAGAGGCAAGTCATTTCAGATAAAAGATGAGAGGTATCAGCATTTTTTAGGAGGCCCTGTGCCTATTGTAGTCCTACTAACTGAATTTCATTATACACATGCCTCTTCTACATTTCTTTTAAATCAAGATGCTTTTGATGTATGCACA

> ST0007

ATGAAACACAGGTTTCCTAAAATCCACCCTGTGGTGTTAAGATTTGAGATTAAAAAATGTACGAGGATGAGAAAGTATTCCCAATGTTCCACGTTCCAAAACCAGCTAGGTTGCAGTCCTCTTTCTTGGTGGGGTGACAAGGTGTGGCAAAGGGAAGCGGAAAACTTGAGGCAACAACTACAGTATTTGCAAGAAAGCCATAGGAAGCTTTTGGGAGAAGAACTTGCTGGATTGAACATCAGAGACCTCCAAAATCTGGAAAATCAACTAGAGATGAGCTTGAAAGGCGTTCGTGTGAAAAAGGCAAGTAACAATCATGAACAAACTCTGACTACTCAAATCAACGACTTAAATCAGAAGGGAAACGTCATCCATCAAGAGAATATGGAACTTCACAAGAAGATGAATCTTGTTGCCAAGGAAAATGAGGATTTGAAGAAAAAGGTTTATGGCCCGGGAAGCACAAGCGAAGAAAACAGGGTTCCCCAGAATATGCACAGCATCAACGATGGATATCCTTTTATACCTATTAATCTTGAGCTAAGCCAGCCACAAAAGCAAACAAATGGTGCACCAAAAGATGTGATGAATTTCGGGTTAGCTCTATGCATCCCTAATTAA

> NAT0008

TGGTGATTAACATTTACTTGGATAATTGAGCATCCTTTCACTCGCATACCAATACCAAAAACTGGATTAAACAAAACAATAATGTTACATGCACCAAAAGATATCCCCATTTTCTATATGAATTTGTGAATCAAATAGTATGTAAATCCGCAGTCTGTCCGCTACCATCAACAATGGTGTCTTAAGTCATTCAAGTCTTCAAGTTGTTGCACAATGGCAGTCATAGTTCCAGAGGTAAATATCCACTGTCTTTGGCATAATATAGTGCTACTGCTAGTAAAATGAATAACAAAATGATCCACAAG

> ST0008

ATCTTTTTTGAATAAATTTATTTTTTAAATTCTTTGTTATTTTTATTCATTTTTTAATTTAATTTTTTTTGTTTTAATATTAATTTTTATTATTATGAAATTCTTTTTTTTTAATATTCAACTTAAATACATTCAGTTAAAATTACTAATTTTGATTACTTTTATGAATATTATAATTAAATTGATATTAATTTTGTATAAATATATAAACATAAGGTGCAAAGCGGAGCAAGAAAGCATGGGCAGCGAGAATGACGAAGAATTGAACTGCAAGAAGTATGGAGTCCCGCTTTACGGCGCCGCCTGGGTTCCATCTGCTGCCTTTACCGCCAACGAGTTGGAAACTCCTGCCAAACGCCTCGTTGTCCTAGCCGGAGGCGGCGGAGAAGGCCACAGCGGGATTCCAAATGCCCTCCTCCTCTCGGCCTTTGATTCCGAATCCTGCTCCCTCTCTGATCAGCCTGTGGCTAAGCTGGAACTGGTAGCGATCTGCCTTACAGAATTGGAGTTCATCCTGGAGGAGAAGGCATTATGTGTTCATTTCCCCAAAACTGCAGGTATGTTCAGTCTCTTTTATGCTAATGAAGTAGCACCTATATGGTTTGAATGGGATTCAGCAACGAGCACGGACAACCAAACTTTAAGTTTGAAGTCATCAGAGAAAGTCCTTAAGCCTTTGGAAGATGTTGGACAACAATTAGCGTTGACCTTCAACACTGAGGGAAATTTACTTGCTGTTGGCGGTGAGGATGGTAAGCTAAGGGTTTTCAAGTGGCCAGCCATGGAAAGTATTCTCAGTGAGGCCAATGCTCATTCTTCTGTGAAAGATTTAGATTTCAGCCCTGATGGGAAGTTTCTTGTCTCTGTTGGAAGTGGCCCTGGAAGGATTTGGGATGTTACATCATCATCATCTGTAGCTTCTCTACCACAGGAAAATGTTTCTGCTTTTAATGTGTCGCCTGATGGGAAGCTCCTTGCTATAGGAACAACTGAAGGAGACATTCTGATTATAAGTTCAGCCAACATGCGGACACGAACTGTTATCAAGAAAGCACATCTAGGCATTGTAACCGCATTGGCATTCTCATCAGATTCAAGAGCTGTTGTGTCCGCATCCTATGACTCAAGTTCAAGGGTGACATTGATTAAGGATGAGAAGAAAGGTGGCTTGTGGATCATTTTGTTATTCATTTTACTAGCAGTAGCACTATATTATGCCAAAGACAGTGGATATTTACCTCTGGAACTATGACAACAACTTGAAGACTTGAATGACTTAAGACACCATTGTTGAAGATGGCGGACAGACTGCGGATTTACATTCTATTTGATTCACAAATTCATATAGAAAATGGGGATATCTTTTGGTGCATGTAACATTATTGTTTTGTTTAACCCAGTTTTTGGTATTGGTATGCGAGTGAAAAGGATGCTCAATAACAAGTAAATGTTAATAATATAATCACTTTTTATGTTGTAGATTGGGTACATA

> NAT0009

GCATGGAGACTTCGTTATACTGAGATTGTGCACATGGGACCCCTTAAGTCCATGAAATTTGGTGCCTTCATAAAAGCCATTCGTAGGTACACTATGGTGCGTCAGAACTACGGTACATGAACTGGCGTATCTCAAGTGATACTTTCTCCTGAGCCATTTACAAGAATAAGATACTTGTTACATCATATGCATTTGCAGCAAAGCTCTTAATTGTGTATGCTATGTTGGTATTGTGAGATATCTATAAATGCTACTCGATCAATTACAGTGTTATTGTTACCCAATTAAATCTCATCTCTCTCTCTCTCATACATTACTTTTCCAAATAGATGTTGGCATGTTGCAATCATTCTTGATCTAACTGCTTAGTAAGAAGCCTAATCTAACCGCAACTGCAAAGCAAATCAGCTGCATGAGTTATTCAATAAAGTGAATGAACAAAAAATTATATCTTGTATTACATTATCGAATGAGGGAAATGTCGAGGATGCTAAGTAATCCACGGCAAGAATGCCACAGATTCAGTCACATTTCTGATCGTTGTTTTACGATTCAATTGATGTAAAACCTGACATGATCTAAGTGTGTCCCCGCCAATTGTACCATTGCTGCACAAATACAAAAATGCAGAACATAGTCGCACTCGTTTAGCAGCCTCGAACGCTCAGAAGTTCAGTACTCTGTTTCCAAAACCCCATCAATAAGACCAAACTCCATGGCCTGCAAACGAGTAAGAGCGGTTAGGCTACTACAGTGTAAAATAAACTGATAATACAATCAAGTTTGTGAAATGGCTAAGAAATATAGTCTCATTTTGCATCAATATTCAATAAATAGAGCTCTTTTTTACTGCTACTTTNTTTTTTTGCTCGATTTCTCTATACTAATTATAATTGCTCATCCACAACAACCACCCACTCTGTATATTGGACAGAATCACCTTATGGTTGCTACGTCTCAACCCTAACTTTAACTAACATTCAAAAATTTCAAGAAAACAGAGTATTGATTTTCATATCATGATTCATACACAGAAGCCAGCAAAACATCACCTCAGACACAGATAAGAATCGGTCTTCTCTCTGTGTACTATTGCACTTTCTCAAGAGATTTGCCAGTAAACGCAGCATACATTTTGTCGATTTTCTACATCCAATAGTCATTAGGATAAAGTTAGACTGCTATAATCAATGCTAAATCAGAAGGTAGTGATGCACATTGTCGGTACATTCATATAAAATTCAGGTAAATATTTCAAAAACTCTTGAGATATAACTACTAATTTTAACTTTAAGACCAG

> ST0009

ATGCCAGCAGTGATTACTCCAGGAGGACCTCTGGATCTCAGTATTATTCAGAAGCCGCATAATCTTCATTGGGCAGCCAATCAATTCGCAGGTGGGAGTACCTACTCTGTCTTGGCAATATATGACTGCATGTCCTGGATAAAGCCCAAGGTTGGAACGGTATGTTTTGGAGTAGCTGCAAGCCAAGGTGCTCTTCTTCTTGCTGGTGGAGAAAAAGGAATGCGATATTCCATGCCAAATGCCCGTATTATGATACATCAACCCCAAAGTGGGTGCGGGGGTCATGTGGAAGATGTAAGACGCCAAGTAAATGAAGCTGTTCAATCTCGTCATGATGTATTTTCCCTGGCAAGCTTCAAAATAGAGCTTGGACGATAAAAATCGACAAAATGTATGCTGCGTTTACTGGCAAATCTCTTGAGAAAGTGCAATAGTACACAGAGAGAAGACCGATTCTTATCTGTGTCTGAGGCCATGGAGTTTGGTCTTATTGATGGGGTTTTGGAAACAGAGTACTGA

> NAT0010

CGAGCGATTGTTAATAATTATAATCTCGATGGGAAAAATGGTGGCTGGATTTAACACATAGACAAGAAGAAGAAAAAAAGTAACTCTGGGAATTAATAGAGATTATGTATGATATACATTCCCATATATCAGCGAGTCTTGAACTTAACGGCGGTGACATCTCCGCCAGGCATAAGATTAAACGTCTGTGGTTCGGCAGCTTGAGCTCCGTCAGCGCGAGTGACAG

> ST0010

GTGATTGTTAGAAAATGATGAGTGAAGGCCCACCATTCTCGGCTTTTTACCCCAATCTTCATATCATCACAACATACGTATAAAAGCAGCATATAGCAGAGAAGAAGAAATTAACACCGTCAAAAATGAAGACCACTTTCAATTCTTTTCTCTTCACATTTTTCACTCTAAGTATTTTCTTAGGTTTTCAAGTGTATGCGGTGCCGTATGATTACTCATACGTACTTGGGTGTGTGGCGAAGCCCGGGGCGCCGCAATACAAGGGCGGAATCGCCGTAAATCCGGAATTCGACGAAGAACTCAAGGGATGGGCACCAGCTGGAAATGCCAAAATAGCAACTGCCAAATCGCCCGACGGCAATGCATACGCCGTCGTTTCTAACATAGTCACGCCCCAGTCTGACGGCCTCTCTCTGGCCTTCAACGTGGAGAAGGGCAAACTCTACACTGTTTCGGCATGGTTCCAAATAAGCGAGGGAGAAGCTGCGGTGCAAGGCAAAGTAGTCACAAGCACCGGCAACAAAACTGCCAACTTTATTACGGCCAAGGCCGGTTGTTGGACCATGTTCAAAGGTGGTTTTCATGTCAACGTTACTGAACCGGCTGCGCTCCATTTTGATACCAACTCTACAGGATTTGATATGTGGGTGGATAGCGTCTCACTGCAGCCTTTTACGCAAGAAGAGTGGAATTCCCACCAAGCTGAGAGCCTTGAAAAGGTGCGCAAGGCGAAAGTGAAATTTGAAGTAGCGGATCAATTGGGAAAGGCGGTGGCGGACGCGAGCGTGTCCATCAAACAACGGGAGCCGCATTTCCCGTTCGGCTGCGCCATGAACTTTTTGATCGTGAAAAACGAAGCATACCAGAAATGGTTCTTAGAAAAAGGATTCAGATATGCAGTATTTGAAAACGAGCTGAAATGGCAGGCAACCGAGTTGAAGCAGGGGGAGGAGGACTACTCGGTCCCGGATGCGATGCTGGAGTTTGCAAAGAAGCACAACATCAGGTTGCGCGGCCACAACATCGTGTGGGACGACCCCGAGTACCTGCCGGAGTGGGTAAAAACGCTGCCTGATGACCAGAAGCGCGAGGCTGCCCTCAAGCGGGCCAACTCCGTCGTCACCAGGTACAAAGGCCAGTTCTTCCACTGGGACGTCATCAACGAGAACATGCACTACAACAACATCAGCAGCGTCACCAACCACGGCGCCGACGTCTTCAACCTCCTCCACCACCTCGACCCTCTCCCCATCCCCTTCCTCAACGAATACAACGTCATCGAGGACGTCAGCCTCCAATCCTTCGCCGCCACCTGGAAGTATTTGCAGAAGATCGACGAGATCAGGAAAGCAGGCTACAACGGCCCTCTAGGCATCGGCCTCGAGAGCCATTTCAACTACGTTGAGCCCAATTTCCCCTACATTAGGGCTTCCATTGATATGTTTTATGCCACCGGATTCCCCATTTGGGTCACTGAGTTTGATGTCGACAGCATATACAAAAAATGGACGCTTCAATATATGGAGCCGCTGCTGCACGAGCTTCACTCGCACCCCTACGTCAACGGCATTATAGTGTGGGGGGCCATGGGCGACAAAAAGGACGGGTGCTGGAAGATGTGCTTGACCGACCTTGAATACAAAAACGTGCCGCCCGGAGACATCCTCGACAAGTTCATGAGCGAGTTCATCAGAGTGCCCGATACTAACGGCAAAACGGACGCCACCGGCGCCTTTGAAACTTCGCTATTCCACGGCGAATACGAGGCCACTGTCACTCGCGCTGACGGAGCTCAAGCTGCCGAACCACAGACGTTTAATCTTATGCCTGGCGGAGATGTCACCGCCGTTAAGTTCAAGACTCGCTGA

> NAT0011

ATTGACGCCCTAATCCATGCAATTCCATCTCCGTATGAATTGACGGCTTCTTCCTCTCCCTCTCCGGACATTGCCAACAAAGTTGAAGGAGCAATCAAATTCAAGGAAAAGAGGAAGTGATGGAGTATATATTGTGGGTTGTGTAGTTAGGGTTATATAGAAATAGAATTAAGGAAAAAGCAAATAGAGAAACAAAT

> ST0011

ATGTCCGGAGAGGGAGAGGAAGAAGCCGTCAATTCATACGGAGATGGAATTGCATGGATTAGGGCGTCAATGATCGGAAGAGGGAGTTTTGGGCGTGTTTATCTCGCAACTCTCAGAAACCCCACATCCAAAAGCAGCTCTCTGCCGCCGCTGATGGCTGTAAAATCTGCCGAGGTTTCCTCCTCGGGCTCGCTTCAGAAGGAGAAGGAGGTTTACTGCAATTTGGAAAGGAGCCCTTACATAATCCAGTGTTACGGCGACGAGATTACAATCGGCCGCAGCGGCACCATGGCGTTCAATTTGATGCTGGAGTACGCCTCCGGCGGAACATTAGCCGATAGGATTGGGGAATCCGGAGGGAATGGATTGGCTGAATTGGAGGTGAAGCTTCACACGCGGTCTATTCTGAGAGGTTTGAGACACATTCACGAGCTTGGATATGTGCATTGTGATATGAAGCCTCACAACATTCTGCTCGTTCCGAACGCCGCGGGCGGAATTAGGGCGAAAATTGGGGATTTGGGGTTGGCGAGGAGGGTCACGAAGAAGAGGAAATTGGGGGTTTGTTGGGAGGGAACTCGGATGTACTTGTCGCCTGAAGCGGTGACGGATCACGTGCAGGAGGCGCCGTCGGATGTTTGGGCGGTTGGTTGCATTGTGCTTGAGATGCTCACCGGGAAATCTCCATTGGAGGGGAAGAAGGAGATAACAGGGCTCAAGATTCCGAATGGCGTGTCGAAGGAGGCTAGGGCGTTTCTCAAGGGTTGCTTTGTGAGGAATTCTAAGTTTAGGTTGACTTGTGAAATGCTGCTGCATCATCCGTTTCTTGAAGGTTTGGATGACGACGACGTTGAAGTTGATGAATCGGTGGCCTGCGCATTGGATCAGATTGAATCTCTTGCTCTGGTTTATGGGAGTGATGATGATGAGTTCAGTAGTGGCTCATTTGCGGATGAGCGGAGCCATGGATCTGAGAGTGATGTATCTGATTCAGCTGAATAA

> NAT0012

AGCTGGTTTTGTGAAGGTCTCCATAACCACAAGCACCTTCTGCAACAGATGAATTACACTGTAAACTGTAATGCAATGATAAAGCATTAATGGGAGTATGATACCTGTAAGCATGGAGTCATTGGAGTCTGTGGAGTAGGTTGCGGTAGCGGACTTCCATTCGGCAACTTGGGCA

> ST0012

GAAAAGAAAAGGGTTATGTTGCATTATGATTTATCTACACACACAAAGTGGTTTTACATACAACTTTAGTCTTTAGTATGAAAAAGATACAAAAAGTCACAGCAGCAGTGATTAGGGTAGAATGAATGAATTAATGGCTGCTCTCCTCCACGCCCTTATCTTCTGCTTGCTCTCAAACTTAACGCATGCCCAAGTTGCCGAATGGAAGTCCGCTACCGCAACCTACTCCACAGACTCCAATGACTCCATGCTTACAGAAGGTGCTTGTGGTTATGGAGACCTTCACAAAACCAGCTATGGCAAGTACAGCACGGGACTCAGTGGGATGTTGTTCGACAGAGGGAGTAGCTGCGGTGCGTGCTTTGAGCTGAGAAACGCGGACAACGTACACTCTCCATCCATCCTTGTCACAGCTACTGATTTCTGCCCCCCTAACTACGCCTTACCTGCCGATGATGGAGGCTGGTGCAACTTCCCCAGAGCCCACTTTCTGATGCCGGAGGCTGCTTTCTCTCTCATTGCCCACCTCAAACCCCAAATACTTCCAATCCACTACAGAAGGGCCGCATGCCAAAGACAAGGCGGAATTCGCTTCACTGTCAAGGGCAGTGCTTCTTTCATGCAAGTGTTGGTCAGCAATGTCGGGTTGGATGGAGAAGTTGTTGCCGTAAAAGTGAAGGGATCGAGGACTGGATGGATTCCCATGGGAAGGAATTGGGGCCAGAACTGGCAGAGCAACATCGACCTGGTCGGCCAACCCCTATCCTTCGAGGTCACTACAGCTGCTTGGCGAACACTCACATCTTACAGCGTTGCACCACCAAACTGGAAGTTCGGTCAGACATTCACAGGCAAACAGTTTCAGCACTCCTGA

> NAT0013

GCCATTATTGCCGCGGATATGATTGCTATGGTGAAAGAGCAAACAAGGAGGAGATCAGTCTGTGTTGAGGATTTCTTCTGAGCTGCTGCTGGTTTCTGGGCTGGTGAGATAACAAAAGGTTGAAAATAACGAGTGAGATGAGAAACATGACTGAAGAAATGACTGTGACAGCAGCCATCCATAGCAATCTTTCAGGCCCATGTACTGCATAAGTGGTGTTTGCAGTGCTAAAATTCGTTATTTCTGCTTTGGGAATGTCGTTGAACACTTCTCTTAGTGTCACTCCGATTTGGTTTTCCCT

> ST0013

ACAACAAGAAAAAAGATTAGTGGGTAAGTTTGGACCATAATTCCACTACAATGATGTTGGACCAATGATCTCAAAAAGCTAGCTGATTGAGAGGTATGCATATATATAGCAAACTGCATATAAAAATAAATAAAAAAGTATTATTATTTATATCATCATGCTCGCGTCACGGTGCCAAACGACACAGTGTGTGTTTTCAGTCGCCATGATCCATTATCTCCAGTCTTGAGGCAAAACACAGTCCAAATTAGCATAATGTTTGAGATGAAAAACTAAAGAAAATGATCACACATATCCCATCACATGATATCGGTGCGTTGCCAATTAGCAGCAATACAAATCCCTCTGCAAAATGGTGGGGATACTCTTCATTTGGAGTGATCTTCATTTCTTACACGAGCACTATATAAGGTCAAGCCGAAGCCTCGGATTCTTGAAAACTATAAGGATTGAAGCATGACTAGGTTGTCTCTTTTACTACTCAAGCTACTGCTGATCTTGGTATTTGCTGGTTGGGTGTCACTTTGGGTTCTTAAGCCGACTGAGTTCTGGACAAGGAAATGGAAGGGCGCAGAGGCAAAAGCATCAGCTACCGCGTTTGGCTATAATGGCCTAGATTTTGTGGTCTTCACGTTCCCTCTAATCGGTCTGTCTATAATCGGGTTCATCTACTTGGAACTGAAACATGACGAACCAAGAAACAGCCTAGGAAGGACTACTGCACTCACTTCTCTCTCTAGTCCACTGTTGGTGAATCCATACGTAGGTATTCTATCGGGGGCACAGATCCTCACCTTGTCTCTGTTCATCATGCTCCTTGTCTGGACATTCTATGTTCGTGTTTCTAATGACTTCAAGAAGATGACGCCGCTCAAATCATTCAAACTTAGCACATGGCAATATAAGCTGTTCAGGATGGCCACGCGCTGTGGCTTGTTATCAGAAGCCTGCCTAGCTCTGCTGCTTCTCCCCGTGCTACGAGGGATGTCCATATTCCGGTGCCTTGGCATCCAATTCGAGGCCTCGATCAAGTACCATGTCTGGCTCGGTTCTGCAATGATATTCTTCGCCACTTTACACGGTGGAGGCACCCTCTTCATCTGGGGAATCAAGCATAGAATCCAAGACGAGATGTGGGAGTGGCAGAAGAAAGGTCGTATATACCTCGCAGGAGAGATAGCTCTAGCAACAGCTTTTGTGATGGCGATCACAGCACTTCCACAGATTAGGAGGAAATGGTTTCAAATTTTTTACTACACACATCACCTTTACATATTCTTCATTCTCTTCTTCCTCTTCCACGGTGGAGACCGCCATTTCTACATGGTGTTTCCCGGAGTCTTCCTCTTCGCCTTGGATAAACTCCTCCGTGTGGTGCAGTCAAGGCCAGAGACGTGCATTCTCTCTGCTCGAGTCTTCCCCTGCAAAGCCATAGAACTCACCTTACCAAAGGATCCAAGGTTAAAATACGAGCCAACCAGCGTCATCTTCCTCAAGATACCGAGCATTTCCAAGCTCCAATGGCATCCATTCAGCGTAACCTCCAGCTCAAGCGCAGACGAGCACAGTATCTCAGTCATTGTGAAGAGTGAGGGGCAATGGACTAGCTCCCTGCACAGCAAGATCATTCATGCTGAAAAGCAAGATTCAGAATCAGATAAAAGGGAATGCATCCCAGTCGCAGTTGAAGGTCCATACGGGCCTGCATCTACGGAGTTTTTAAGGTACAATAACTTACTCATGGTTGCTGGAGGGATAGGGGTGACACCGTTCCTGAGCATCTTGCGCGAGATAACCTGCAACGGCAGAAATGCATATCCAGATAGGATACATCTCATATACAGTATAAAGAAGTCACAAGATGTATGCTTACTTGATCCAATACTCCCACAGCTGCTAGATATTGAACAATTTCACACGAAACTCAAAGTATTCGTCACTAGGGAAAACCAAATCGGAGTGACACTAAGAGAAGTGTTCAACGACATTCCCAAAGCAGAAATAACGAATTTTAGCACTGCAAACACCACTTATGCAAAACCAGCAGCAGCTCAGAAGAAATCCTCAACACAGACTGATCTCCTCCTTGTTTGCTCTTTCACCATAGCAATCATATCCGCGGCAATAATGGCTATTGCCATAAGGTGGAAGAGGGTAACGATACTAACAAACGAGCTTCAGTTCTTCTCCAATAAGCAAAGCAAAGCTGTGAAACAGAGCTCACTTGAAGCAAGTAGGGATCTTGATGAACATGAGATCCATTTTGGAGGAAGACCAATCTTTCAAGATATAATCTCAAATTTCGCTAGAGAAACTGGTGGATCGGATATAGGGGTTTTCGTCTGTGGCCCTCAAGGGATGAAAGACTCTGTTGCCTTAGCTTGCAGACGAGCTCGGGTTTCACAGAGGAATGCTAAGGGAAAGAGACCAAAACTCACCTTTCACTCACTGAACTTCACACTCTAGCTAGTTTGCAGACAAATTGCAATATACCTAATTTGTGGCCATGCGACACTGTAATAATTAAAAGTGTAATAAGATAGTCTGTAAGGAAATGTAGCCAAGCACTTTGTTCATAAATGGAAAATCCCAACTATATGATACAACAACAATGGCAAGAACCTCAAATAATCAATCAAGTCACACAACAAAACTGTATATATACATATCAAATATCAAAACACCTACAAGAACAAAATAGACCTTCACACCTCCTCTTGAGGAGGCAGGAACACCTTTTCTTGCTCCTTTTCACCTTCATCTCTATCAATGATGCTCTGTACCGGAGTCAATCCAGACAACATATGCCTCGGCTCCATCTCCACGTGCTCGCCCAACAACATTGCTGTGACCTCCTTCATAGTAGGCCTCCGGCTAGGATCAGGGTGGCAGCAAGACAACCCAAGCTTCAACACGGCCTCCATCTCCCCTACCTCGTACTCCCCCTCAATCCTCGCATCCGCCACCTCCAAGATCCTCCCCTGCACATACTTACCCCTCACCCAGTCTATCAAAATCTCCTCATCCTCGTCCGCCACCCCCGTCATAATAGGCTTCCGCCCACACGCCACCTCCAACACCACAACACCAAAGCTGTACACATCACTAGCTGCAGTTGGAGAAGCCAATGTCACCACCTCAGGCGCCAAGTAACCCAACGTCCCCACTACCCGAGTCGTGTTAGGCACCTGTCCATGAGTGTACAGCTTCGCCAAGCCAAAATCACCTAACCTACCACGCAGATCATTATCCAGCAACACATTGCTGGACTTAATGTCTCTGTGAACCACCACCTGATCCCAACCGTGGTGAAGGTAGCTCAAACCCTCTGCAACATCAGCAAGAACCCTCTTCCTCCCCTCCCAACTCAACAAGCGCTTGGGCTTGTCAAAGATCCAATTGTTGAGGCTTCCATTAGGCATGTAGTCATACACTAGCATCAGCTCATTTCCTTTCCTGCACCACCCTTTCAACTGCACTAGATTCTTGTGCTGCAGCCTACCGATACTCGAGATCTCAGCCATGAACTCCCTCAACCCCTGCTTGGAGTCATGGTTCACGCACTTCACCGCCATCTCCATGTTGTTGCCCAATGTAGCTTTATAAACCTTCCCAAAACCACCCGATCCTAAAAGCTCTTCACTCGAAAACGCCTTTGTGGCCACGCTGAGCTCCTCGTATGAGTATTTGTGAGGCCAATATTCCATCTCCCAATCCTCAATGTCATCATCATCGTCATCCAAATTTCCCCTTTTTCTCCACCAAAACCAATAGGTCAAGAGCAATGCAGCCGCCACCACCGCTGCCACACACCCCACCGCGATCCCCACAACCGCCCCATTCGAGAGGGAAGACGACCCCGGATTTGCCGGCCGGAAAATTGGTAAACCAGTAGTGTTGATATCCCTAGCAATTCCTTCATCGCTAAAACTCCAAGCTAGCAGCCTCTGCACCTCAACCCACTCCGTTTTTGAAGCCGAAAATCCGACAAACATCTCCGCCGCGATGTAGTCTGCAATCAACGGGCTTTTATACGACAGCAGAGTCCTCGCCGGGCGGGGCATTCCGGCCGGCGCGATCGTGACATTGATTTCATTTTCCGGGCCGTTGAATTCGATCCAAGCGTGGACATTCTGCCCACTTCTCATATTAACTTCCACAAATGTAGAATTGGAATCGTAATACCCAGCCGTTTCGGTTACTTCCGACAACACGCTGTTCAAATCGACGCCGATGTGGTTGGAATTCACCTCGTTGAACTCGGTGTTCCGGCCGGTGTCAAATTCGACGGCGAGGAGCGGCGCCACCGTGCGCGGCGGGGAGGTGAAGAGGCCGAAGTACTGGCCCGCGACGACGCGGGGAGGGGTGGTGGTGTTGGAGAGGACGAAGGCGAGGCCGAAGCCGGGGCTGTCGGCGATTTCGGGGAGGATTGAGAAGACGAATTGGGTGGAAAACGACGTCGTGGCGTTGGTGGAGTTTGGGGGCTTGAAGGGGACTCTGTAGGGAAGGAAGGCGCGGCCGCTTGAGTACTGCTCGGAGTCGTTGGTGAAGCGGATCACAGGCGGGTCGATGCGGGCATGGTTTATGAGGGTGAGGTTGGCGCCGGCGGGAAAGGAGTTGAAGATGAAGTCGAGAGCTGAGGATGGAGCTGCTGCCAACTGCAGGAACAGGAGGAGAAGCACTGTAGGGAGCATTATTCGGGTTGGGAGTTGCAAGAACTTGAAAATCCAGAGATTTGGGGGCGTGTCAGAGCTCCGACATGATGAGGTTGCCGTTTTCGGACAAAGATAAACACAAACTCCACAAAGTGGACTTTTACTTTC

> NAT0014

TAACTAGTCTTGTCGAAGAATTAGAAAGGGAAAAGGATGCTGGGAATAACCAAGGTGTTGAGCAGAAAGCACGGCGGAGCAGCCGTGTTCCGCTGCCTTGGGCGGCGGTGGATGGTCGGCAAAGCGGCCAACCGCGCTGCCGTTCATGGCTCTAAGCTTCCCGTGCCTAATGCCATCCCGGTGAAGGAGTTGGAAATGGAGATCCACAAATCTATAGAAGAAATGGAAAGAAAGAGGAGGGATATAGAAAATACAGTTGATTCCAAGCTCAAAAACTGGCCAGTGCTCTACTTTGAACAAATGGTGTGTTCCAAGTAAATAATATGTAGTATGAGAATATTTGACTTTTTTGTCTACAAATAATCATCATTACCTTGTATAATTCGCACCAATGTAATGAATTTCAAATGAAAAAAGTTTCATCACCGTTGCAACAGCT

> ST0014

CACGACTAGGTCGATGTAAGATGTTAAAAAAAATAGAAAATTGTTGATATATAAACCAACCCAAGAAAAGGGGAAAATAAATAAGTAAAAAGATATTATCATTATCCACCGCCGATCTCCCACACAATACACTCTGTGTGTGGCTCTCGCTTCGCTCCGATTTATCATATTTCAATTCCTTCTCCGTCAATTCACGAACCCTCTTGCTTTATTCAATCTGATTTCAGTTTGCTTGATACAATTTGAAGAATTGCATGGAATTCCCCCAGACGGCGGCGATTTGTAGTTGTACTTGAACAGTGTGCTTTGCATTCCCGACTTAATGAGCTCCCCATGCCTTTATTAGATATTGCTATTTCAAAACCTTGCTCTTGTTTCCTCAACAATATAATAGCATTTAGGTCAGAAATCGATCTTCGTAAGAAAATTGTAGTTGGCAATGATAGGAGATTGGTATTGGCATCCACATTTTCTGGGAGGAATTTTTGTACTGGAGGAAAAGTGGGAGGAGATTTTATCTCGGGGAGAGCCGGGATATGGGGGGTAGGCTGATGATAAGGGCCGTTGGTACGCTCGAAATTGCTAGTGTACCTTGTAAAAATGGAGGGGTGAAGAGGCTTCAAAATGTATTGATGATGGATGTTGATTCAGTGAGGTCGAATTCCACTGATTTTGAACCGCCACAATCTTCAAGTGAAGTCTCAACAGATGTGGATGACAGGGAGAAGTTGAGACGGATGAGGATTTCCAAAGCGAATAAAGGGAATACACCGTGGAACAAAGGCAAGAAGCACAGCCCAGAAACCTTGAGGCGGATAAAAGAAAGAACAAGGCTGGCAATGCAAAATCCTAAGGTTAAAGCCAAGCTGATTAACCTGGGCCATGCTCAAAGCGAAGAGACAAAGTTGAAAATCGGAGCTGGTGTTCGGCTAGGATGGGAGAAACGCCGTGAGAAGTTGATGGTCCAGGATACTTGCTACTACGACTGGCAAAATCTTATTGCAGAGACTGCCCGTAAGGGCCTTCAAGGTGAGGAACAATTGCAATGGGACTCGTACAAGATCTTGGATGAAGAACTTCAGCAGCAATGGCAGCAGAGCGTCGAGAAAAGAAGAAGCATGCCAAGACTGAAGGGAAGCAAGAGAGCACCCAAATCTGCAGAGCAGAAGAGGAAGATCTCAGAGGCCATAGCTGCAAAATGGGCTGATCCTGAATATCGAACTCGGGTTTACTCTGGTCTAGCTAAATATCATGGCATTCCGGAAGGGGCTGAATGGAAGCCTAAGAGAAAGCCTGCTGGTGGACAGACTCGAAGCAGAAGCCGCAAAGACAAAGATGAGAACGACGATGCTGCAAAACACAAGATGAAGAGCCTAAATCAACTTAGAGCAAAGAGAAGCAAAGCGCCATCATACAAGGACCCATTAGCAAGCACCAAACTTGAGATGTTGAAAAAAATTCGAGCCAAAAGAGCTGCTGCAGTCGATGACCCGAAAACTGAAGCCATCACGAAGGCCAAGTCGTTGATTGCAGAAGCTGAGAAGGCTGCCAAGGCACTTGAAATAGCTGCTAAGACGAGCCCTCTCGCGCATGCTTCCTTAATGGAAAGCAGGATGCTGATCGCGGAAGCACATAAGCTCATCGAATCCATAGAAATCGAAGATGCAGCAGCATCCTTTGAAGATGAGAACGGAAATGATCTATCCGAGCGAGTTCCAAATCTCGTAGAGCCCATGAAAGTAAATGGAGTCCACAGCATCTCTGCTGATCTTGAAGAAACCGATAGTTTCAGTTTTGATGAGTTCATTTTCGCTGATTTCATCAACGGCAACAGCTCAAGTCCTTACCCTCTTGAAACGGAAGAACGCCGGCCTAATGGCTTCGTCTCATCGGTTTTGGATAACATGATGAACAGCTCTGATCAGACCAAGAAGCACGTCAATCCTAAACCAAACCTTAATGGGATCTCAGTTCATACGCGAAATCTGGCTGACAACTGGCCGGAGCTCCGATCAGAGAATGCAGAAGCAGCTATTAAAGAGGCGAATGTGATCAAGAAATGGGTTCGTGGGAGGCTCGTTGAAGTTGAAGAAGAAGAAGCCTAGGTTAGTTCTAGCATTTTTAGAATTCATGCATAGTTTCTATATCCATATTGCTAGGAAATATAGAGTTTTCGATACTAGCTTATTATATCCAGGTTTTCATGTCCAAGAATCATCCAAGTTTCACTTAAATCTCAATATTCAATTAAGAGATTCGAAATGCAATACATGTCAAAATTTTGAAAATTTTATTGCATTGCAGCTGTTGCAACGGTGATGAAACTTTTTTCATTTGAAATTCATTACAT

> NAT0015

ACGGCCACAACCACCTTCTTAGGGCTGCCGCTGCCGCCTTTCCTCAAAATTGACATAGTCCCAGTGGCCTTCCAGGGAGAAATCAACCAAGAATCCGGCAAGGTTGATCTCAAGTTCATAGCCGAGTTCTGCTTCTCGGTCGGTAGCCTGTACAAAGCGCGGCCGCTGCTTGTGGAGACGGTGTTGACGTCGGAAG

> ST0015

ATGTCAATCGTGACGATATACACCAATATTACTATTACAGGCAAGGGGAAATTTTTCGAAGTATTGATTGGGGCAAGACCAGAACCTATCTGGCAGCTGCTGAGTAAAGTGGGAATACTGTATACATTGGAACCCATTTTCACAATTATTTTTGTGGTGGAGTTTTTTGACAAGTACAAGGTTGGCGAACTGACTTCCTTATTAACATCGGATTTGGGTTCTCTGAAGACCATTGTTAGTGATAACTATCAAGAGACCGTCATTGGAACACTTTGCTTACTTTTTGCTCTTTCCCCCCAGCTGGCGCCAGTTTTAGGCCTACTTATGCTAACAGTGTCTGCAGTAGTTGCTGTATATAAGAGAACAACCGTCAGTGTCTTTAAAGTTCATGGATCTGCACTAGCCACTATAGCTGATTGTGTTACTGAAACTTTTGCTGCTATACGTACTGTCAGATCATTCGGGGGTGAAAAACGTCAAATGTCAGTTTTTGGTCGGCAGGTTCTTGACTATGAGAGAAGTGGCATGACCCTCGGAGTATTTAAGTCCATAAATGAATCCATCACAAGGGTTGCCGTCTATGTCTCTTTAATGGCTTTGTATTGTCTTGGAGGAAGCAAAGTAAAAGCTGGCAAACTTGCAGTTGGAACCATGGTTTCTTTTATTGGATACACTTTTACATTAACATTTGCCGTTCAAGGTGTGGTGAACACATTTGGGGATCTTCGAGGAGCCTTGGCTGCTGTTGAAAGAATTAACTCGGTCCTGCCTGGTGCTGAAATTGATGAAGCACTAGCTTATGCTCTAGAAAAAGACTTGAAGAGAAGGAAATCACATGATCCAAACCTCGAACCTCTATTGGCCAATTCCAACGGGAAAATGCAAACCAGCAGTGTGGGATACATGTCGTCACTAAAATCTGCTAGTGATGTGCGCAAGCTTGCAGAGTCCGGTGATATTCACCTTGAAGATGTTTCTTTCTCATACCCTCTAAGGCCCGACGTGGAAATCTTACAAGGCCTTGATCTGACTTTAAAATGTGGAACGGTCACTGCTCTGGTTGGTCCTAGTGGTGCCGGGAAAAGTACAGTCGTACAGCTTTTAGCTCGCTTTTATGAGCCAACAAGAGGTCGCATAACTGTTGCTGGAGAGGATCTGCGATCATTTGATAAGAGTGAATGGGCTCGTGCGGTTTCCATAGTGAACCAAGAACCTGTTCTATTCTCCGTGTCTGTTGGAGAAAATATTGCTTATGGGCTCCCAGATGACAACGTTTCCAAGGACGACGTAATAAAAGCTGCTAAAGCAGCTAATGCTCATGAGTTCATAATCTCATTGCCACAGGCTACGAGTGCTTTGGATACTGTGAGTGAACGTCTGGTTCAGGATGCTTTGACTCGTCTGATGAAGGGAAGAACGACTTTAGTGATTGCTCACAGACTCAGCACGGTTCAGAATGCTGATCTTATTGCTCTGTGCTCCGATGGGAAGATTTCAGAACTCGGGACACACTCAGAGTTGTTAGAACAGAAGGGCCTATATGCTTCTCTCGTTGGCTCCCAAAGACTTGCATTCGAGTGACAGTCACTATGCACTGATCGTTTCACAAAATTTCTCTGCCAAAGCATGCAAATAAACTCGCTTCTGCAATTTGAAACGGATCGCCAATGAAAAAACCGCGAATGCTTCCGCACTAACATCATGGAAGGTGAAACGCCACTTTCTGATACACTGGACAAAACAACGTCGTAAAACCGTTATTTCATATAGTCTTTTTGATATATATATAGTATATATATATGCAGCAAAAACCACCTGCAACTCTTCATTTTATACTGAACTACATTGCTGTATCATTGAACATGGCTTACATCACATACGCCATACATATATATATGTTACAGATTAATATTTCATCAAGGTAAATACATGAGTATAGTTTGAGAGATATGTGTATCTTTTCAAATAAAGGGGGTATTATTCTGTGGTTGAAAATGAGATAACAGCATTCAAATTTGCGAGACATTCCGTGGGGAGGCTAAGAAATGTGTCCATGAAAAGGTCGTTGATGGGCTCAACCGTCGCCACCCCAACAAGCCTGCATTTCCCATCTCCATCCATTCGCTCTCCTCTTCCCTCTCTCATTTTTCCCTTCGATTCTTCCGACGTCAACACCGTCTCCACAAGCAGCGGCCGCGCTTTGTACAGGCTACCGACCGAGAAGCAGAACTCGGCTATGAACTTGAGATCAACCTACATGCCAGATTCTTGGTTGATTTCTCCCTGGAAGGCCACTGGGACTATGTCAATTTTGAGGAAAGGCGGCAGCGGCAGCCCTAAGAAGGTGGTTGTGGCCGTGGATAGGGGTGGAATGGAGAGCTTTTTTACGTCAAACTCGACGTAAACTTGGCCATCAGAAAGCTTTGTGGCTGTGCCGGAGCCTGCACCGCCGGAAGCATTGTAGTCGAAGTCAGGGTATCGGGAAATGCCGAGCTTGCAATCTCCTAGAGTTTTGAAGTTAACGTTGGATGAATGAGTTGTGGGGTTTTGTGTTTGATTGATTGTGGCAGTTGAAAGTGGTTCGTTTAGGTTGTTGGAGGATAGAGAGGTAGTTTTGAGGAGTTTAAGGTTTGGTTGGAGAGAGAAACGTCTTGTGTTTGGTGCGTGGGTTCTGTTGTAGAGAAATTGAGGATTGAGATGGAGGGTTTGACACCCCATTATATGGTGTTGTTTAGTTTCTGCAAAAAGAGAATGTTTGAGCGTTAAACTATAATACAGTACATTTTTTCAGAATATATCGGAGCTTCCATTTTAGATAGTCTAATGTGAGGCCAGTATGAGACCATGAGTTGAGGTCCTCATCCTCTCTCTCACACACACACAC

> NAT0016

CTCAATTTCCGTCTCCTCCTCCAGAATTGACACTCCTCGAACCGGTGGCTCATCATTTGCGATCGCCGCCCTCACCCTGAAATTCCGCTTCGTCTTCATCGTTTTTCTGATCGGGAGGGCGGGAAAACTGGTTCGTTTAGTAGGAGATGATGCATTTTTAACGAGGTTTTCAGAGGAATGTGGGAGGGTTTTACATGAGAAGAAGTTGTTTACACAAGGCATGGAAGCTGCCATTTTCGGAAGAAATTGTTAATGCCGACTCCTTATATTG

> ST0016

ATGAAGACGAAGCGGAATTTCAGGGTGAGGGCGGCGATCGCAAATGATGAGCCACCGGTTCGAGGAGTGTCAATTCTGGAGGAGGAGACGGAAATTGAGGTACTGAAGAAACAAGTTTTGGAGTCGTTCTGTGGAGCTAACATGGGTTTGAGCGCCAGTAGCGAAACCAGGGCGGAAATCGTCGAGTTAATCACTCACCTTGAGGCCAAAAACCCTAACCCGGCTCCCACTCAGGCTCTCACCCTGCTCAACGGCAAATGGATTCTCGCCTACACATCGTTTCTGGAATTCTATCCTCTATTATCAAGGGAAGGAGCTTTCCCGCTGGTGAAGGTTGAGGAGATATCGCAGACTTTCAACTCCGACAACTTCACTGTCCAGAACTCCATCGTCTTCTCCGGCCCCCTCGCAACAACTTCACTTACCACTAATGCCAAGTTTGAAGTTCGAAGTCCCAAGCGTGTGCAGATTAAGTTGGAGCAGGGCGTTATTGGGACACCCCAGTTGACGGACTCCGTGGTGTTGCCGGAGAACATAGAGTTTTTGGGACAGAATATCGATCTGGCGCCGTTCAAAGGCTTGCTCACTTCTCTACAGGACACTGCATCCTCTGTCGCAAGGTCCATTTCCAGCCGACCGCCCTTCAAATTCTCCATCTCCACTAGCAGCGCCGACTCGTGGCTGCTCACCACCTACCTAGACCACGAGCTTCGCGTTTCCAGAGGAGATGGTGGCAGCATATTTGTGCTCATCAAGGAGGCCAGCCCCCTTCTCACTAAATCCTAG

> NAT0017

GGAGTGGACGGCGGCAGCGGCGACTTCACAGCAAGGGCGGAGTTTGCGGCGGTCTGCAGGAGACTGATTTCCGGCGGAGGAGGGTGGTGGGCGGCGCGATTGTTAGGGATTTAGGGATTTAGGTACGGCGGCAGCGGCGACTTCACAGCAAGGGCGGAGATTGCGGCGGTCTGCAGGAGACTGATTTCCGGCGGAGATGGTGGCGGAGGAGGGTGGTGGGCGGCGCGACTGTTGCTTTTGAGAAGGGTTAGGGATTTAGGGATTTCAAAGTGAGGAACAAGCGTCACTGATGCTTTTGGTAGAAGGCCGT

> ST0017

ACCCTCCTCCGCCGGAAATCAGTCTCCTGCAGACCGCCGCAAACTCCGCCCTTGCTGTGAAGTCGCCGCTGCCGCCGTCCACTCCGATCTGCTGGACTTCCAGAGAAGGCGAAGTCTCCGCCGCCTGCCTCCGCCGTCCATTCCGGCCGCCTGCGTTGAACCTGCCGCCCTTGCCTCCGTCGGCCAATCTAACTCCATCGTCTCCTTCATTCTGAGCTCAGAAACGCCCCAACCAAAATCTGTATACCACTCCATCCTTGATTCTTGTGGAATAAAGAAACTAGTCGAGGACAACAAGAGAAAAGAGAGCAAAGAAGTGTGATCAATAAAGGGGAAAAAGAGGACGACGAGTGAAGGAGAACAAGAAAAAAGAGCAAGGAAAAAAAAAAATTAAGCTTGTAACTTTTGATCATAGACGAATAGGAGATATGGGTCGTGGAGTTAGCAGCGGTGGAGGGCAGAGTTCATTGGATTACTTGTTTGGTAGCGGTGAGGCTACAAAACCAGCAGTGGGGAAAGCACAAGCTGCACCGAGTGAGAGTAGTGTTGCTAGTAAAGAACCTTCGCCAAAGCCTAATGCCGCTCCTAAGCCAGTAGACGTTAGCAAGCAGATTCCTGCAGGGATTCATAGTAGCACCACCAACAACTACGTCAGAGCAGATGGTCAGAGTACTTGCAATTTTATCACGGATCGACCATCAACTAAGGTTCATGCTGCTCCTGGAGGAGGTTCTTCTTTGGGTTACCTTTTTGGTTGCGGCAGCAGCAGCAACTAAGCAACAGCCAGGGTCCTGCAGCCGGGATCGCCAAAGTGTGTTTCGAGACATTTATTTGTAAAAATGTGGCAGCAATATGGTATTTTGCGCCTTTGCTGCAAGCCTCGTGGGATGTTTCTGTAAGGTTTTGGGTTATTTGTTTGTAAATGTAGCAGCAAATTATGGTATTGCGTCTCTGCTGCAAGAGTTGTCAATAGTTTTTCTACAGACAGATATGAATTCGTTGGTAAATTTGAAGTGTGAATCTCTACATCTTAGTTGTGTTTGGATGCATAATAATAAGCATCTCTCAAAAAGGCTTCCTTTGTAAAATAACTACACCGTATTCTAGATTGTATTATTGTGATTTTATGAGATTTTTCATACTGGAACAGGGTATTATTACTCTTTGCTGTTTGGAAAAAGAAAAGCTCCTAAAAAAACAGATTCTATTACAAAAATAAGCCAGTAAAATTATGATACAAGATCAGAAAGGCTACTATCA

> NAT0018

AGGGGGGGGCGCTCGCCGGCGGCGGTACCGCCGTGAAGGCAGTGGCGGCGGCAGAGAGGCAACCGAGAGAAATGAGAGGTTGGTGGCCGCAAGAACTTAATGATGGTGATCTTCAAAAGACTGATGCTTTATTCCATCGCTGTATTCAAGATGGGCGTGGTCTCATTAACCAACTTCATGGCAGATTTTGCCCCAAACGAGATGCTTTGAGTATTTGCAAAGTTCGGAATCTATTGTCATTTTTCTTTCTTGACGTCTCCACCGACCTGACTGAAGACCATGTCCGGAGAGTTCATCCAAAAATCTGGCAGCAGCCCATCTAAGACCTTATTTGAGTGATGTAAACTTTGCACTTCAGGTGGCTCAATTGGCTTTTAATCAGATTTCGGGTGCGCTCGGATCTGGGCCATTGTGAGTCCAACCTTTGAAGATTGCTGGTTACATTTTCTCTTTGCGCGCATTCGAGCAGGCTAGATGACTCAATGGTGTTCTATTATGTTTGGCGAACATGTGTTTCAACTCCACGAACGAATTCAACTTTAGCTTGGAGAGAAGTGAAGTTGTCTATGCATAGCCGACCTCAATGCCACATGACTCAATTCTTTGACAGGTCTTGGGTGCGCTCAGGCCTGGGATTGTTTGAATCTGGTTTGAGTGAGGTTGTTGTTATGTTTGGCATCTCACGAAGTTGGGAGAATTCTGCACAACGGCTCGAGAG

> ST0018

ATGAAAGGAGGCACTATCTTGCTTGAAGGACAACGTCATCTCTGTCTTCCACCTCTCTATCATAGCAAGCACTTCCTTGATCAAAGCAATTCGACGATGGCTTCATTATCCTTGGCTAAGCATCAAGATGAAAATACTCAATCTCTCGAGCCGTTGTGCAGAATTCTCCCAACTTCATGGGCTGCTGCCAGATTTTTGGATGAACTCTCCGGACATGGTCTTCAGTCAGGTCGGTGGAGACGTCAAGAAAGAAAAATGACAATAGATTCCGAACTTTGCAAATACTCAAAGCATCTCGTTTGGGGCAAAATCTGCCATGAAAAAAGAGTAGTTTACCCTCTCATTTCTCTCGGTTGCCTCTCTGCCGCCGCCACTGCCTTCACGGCGGTACCGCCGCCGGCGAGCGCCCCCCCCTCTTCCTCGATATCTCCCTCTTTTCCTCACACAACCGCCATCGTATCTCTGCCAGCAAGAGACAGGGACACAGCACCGCTCTTCTCCCTCGCGAAATCTGCCATTGTGGCTCCTCCTCTTCCGGCGAGGTCGACGCATAGTCGGTTGGACGCCCGCCTTCTCAAAGGATACAACAAGCTGGAAAAGAACGCCGCTGAAGATACGCCGCCGCCTGCTCTAGTCCCGGCGAGTCGACGGCAGCCGCGCCTCCTCACGGCCCATCCATTCTCGATTCTTCTCTCTCATCTGTCTGCTAGAGATTTTCACAAACACCTTCTACCCATGGCTGTCGAGATCTGCGTCAAGGCTGCTGCTGGATCCCCCGACATTGTCGGAGACTGCCCCTTTTGCCAGAGGGTGCTGCTGACTTTGGAGGAGAAGAAAGTCGAGTACAAGCTGCATTTGATCAGCTTCGACACCAAGCCCCAGTGGTTTATGGAAGTGAATCCAGAAGGGAAGGTGCCGCTCATAAAGTTGGATGACAAGTGGATTTCTGATTCTGATGTTATTGTTGGTATTTTAGAGGAGAAATACCCAAATCCGCCTCTCCGCACTCCAAAAGCTGGTTCCGACTGCTGCAAATCCGACAGGTCATTGCTGCCATCGCCACCTTTGAAGACTCCTGCTCCGGCATCTATTAACAGTGTCTGCTCGAAGTCGCCGTCGGCCTTCAATCTTGCGCAAAATCCCGTCACCGACATTCGCAAGTCAGATGCTGCCTTCTCTCTCGACGCCTCTACTGTCAAAGCCCGATCTCTCCTTTCGCCGGGACTACGGAGCAGCACACGTCGCCGTTCGACGCCTCTACTGTCAAAGCCCGATCTCTCCTTTCGCCGGGACTACGGAGCAGCACACGTCGCCGTTCGACGCCTCTACTGTCAAAGCCCGATCTCTCCTTTCGCCGGGACTACGGAGCAGCACACGTCATCGTTCGACGCCTCTACCTTCAACCGCCGCTAG

> NAT0019

TGCTCTCTCTCCACCTCTCACCCCTTTGCTTCAATTCCGACCGATCCCCAATCGTCCCATCTCACTACGCATCCCGATCAGATATCCAATGATGCACAGATGATTGATAATGAAATGGAGAATCTTGAACATGAAGATGAGGAAGAAGGAGATCCCATTTTCGTTCTTACCGATGAATGGAGGGAATTCTTCGCAAAGTCCGATGCCAAAAGGAAACTAGCGAAGAAGCAAGCTAAGAAGGGGA

> ST0019

GTTTAATTTTAGCTCTCTATATTTTATAGTGAAAAAAAGGTTAAAAAGAAAATGAAACCGAGAATAAAAGCGTTGAATTCAAAATCGCAATCAAACTGAGTGAAAATAAAGAAGTACACAGAGATAAAATCTGGATTTTTCCCTCAATTTTTTGTAGCTTGACAATTGTACGCTGAGAGTACGTTGCTGACTGCTGAGAGTACGCTGCATCGCGCCCGCCCGTCTCCGCTGCCTCGCGCTCGCACGTCGCCGCCCCCTCGACGCTTTCTTCCTTCGCGCGGCCACCATCGCCCACCGGCGCTGCCCGCCTCTTCCCGTCTCCGGCAGAACTAAGATCAATCCAGCTACAAAGCTATGGCTGCCAATTTCTGGACTTCATCTCACTACAAGCAGCTTCTTGACCCGGAAGAGGTGGATGTGGTGCATCCACTGGATAAAGAAAGGGGCATAACTGTTGAAGATTGCAAGCTCATCAAATTCCATATTTCCAGCTATATTGTGAAATTGGCACAGTATATCAAAGTGCGGCAGAGGGTGGTTGCTACAGCCATTACATACATGAGACGTGTCTATACCAGGAGAAGTATGACGGAATATAATCCGCGTCTGGTTGCTCCAACTTGCTTGTACTTGGCAGCCAAAGCTGAAGAAAGCACAGTGCAGGCCAGACTTCTTGCTTTTTATATCAAGAAAGTACAAAGCGATGAGAAGTACCGGTGTGAGATTAAAGAGATACTAGAGATGGAAATGAAAATCTTGGAAGCCCTCAACTATTATTTAGTTGTATTCCATCCTTATCGAGCACTGACGCAGTTGCTTCAGGATGCTGGCATGACTGATTCAACGCAACAAACTTGGGGACTTGTTAACGACACGTACAAGATGGATCTGATTCTTGTACACCCTCCTCACTTGATCGCATTAGCCTGCATATACATTGCAAGCGTCTGGAAGGATAAAGAAAACACGGCTTGGTTTGAAGAGCTTCGTGTTGACATGAACGTGGTGAAAAACATTGCAATGGAGATACTTGATTTCTACGACAGCCACAAAGTGATGACAGATGACAACAGGATTAGTGCAGCAATGAACAAGCTACCATGATCAATCACTATGTTTTTGTGTAAACAGCAGAAACGCGACAGCACAATGGCTTCCACAACGTTCAATAATTCTGGAAAATCAGAGCCTTGAAACGGCACATCACCACATCAATGTATCGAATGGCATTCTGTAGTTTGCTATCTAAAAAGGCAACACTTTTCTAATGCCTTTTTCATGCATGTGTTGAGATAAAAGTTGAAAAGCGGTATAGTAATCCACAAGGACATCTGGGATCAAAGCTTGGAAGATTAAGGTACAAGAAAGACTTAAAAACCCAGCTTGAAACGTTCTTGAACTCAGAAAGACAGAAAATGGAGCAATCACTCCATTGTATCAAGAATTAAGCTATCCAAAATGCAAAGAGTTTTTCTTTTCTACTTCTTCCCCTTCTTAGCTTGCTTCTTCGCTGCAAGATTTCGATGGCATCGGACTTTGCGAAGAATTCCCTCCATTCATCGGTAAGAACGAAAATGGGATCTCCTTCTTCCTCATCTTCATGTTCAAGATTCTCCATTTCATTATCAATCATCCTTGCATCCATAGGATATCTGATCGGGATGCGTAGTGAGATGGGACGATTGGGGATCGGTCGGAATTGAAGCAAAGGGGTGAGAGGTGGAGAGAGAGCACGACGGAGTGTGATTGCTTGGTTGCAGAATAGGGCGGTGGAGTGGGCAGCGAGGCGACACGTAAAAGTAATGGCGGTGGGCGCCGCCGCAGTGGTGGTGGTGGATAGGGTGTAGTGATGGGCTTTTGCCGCCGTTGTCGTTTGAGGCCATGCCGGCGGTTGAAGATGACACCTTTTTTACAGCTTAAATTTATTTATGTGGTTTGAGTCGGTATCTTTGACTTCTCCCCTTTTTTAGGGGGCGAAAACAACATA

> NAT0020

CATTTTTCGGGATTTTCACGCTTGGACTCAGGATGTCTACAATTGAGCCACGCTTCAGGCCAAGAGCATGCGCACACACACACTTATTTAAAAGGAGCATTTGATAAAATCACCTCAGGGATAGGGATCTACAATATGCCTCATGGGATTGGAAAGAAGGAATAAGAACTTGTTTGCTGTGGTGATCAATACGGGA

> ST0020

GCTCTCGCCGCCTCCCCTCCCTTCCTACGAACCGGCGTCTCTTCGCCTTCGACCGCCACCACACTCATCTCTCTATCCAATTTCTCGCTCAACCCCCCACCGCTTGCTCTCCCTCACTTATCTCGGAGACAGATTTCATTTCTCTTCAATAAATCATAAGAAGCCCAACCATCCAAACAGATACGACCATTCAGTTGCAGAGACAAACTTCCGCCATTAAAATCCGATCAATGAACACTTTGCACACAACCACTCTCTCAAACCTTATCTCCCAAAAATCACCAATTCGCCGTCGCGCTCCTCTCCGCTCCCACAGACTCCAACCATTTCTACCAAATACTAATTCAGCTGAACATCCAAAATTATCAATAAAACCTACGAGAATCGCGGCAGCGTCAATGGCTTCCACGCCGTCAACTGCCTTGGACGATAGCGCGCCGGCGAGCAAACCGTTCTCTGTTCTGTTCGTCTGCCTCGGCAACATCTGCCGGAGCCCGGCGGCTGAAGGCGTTTTCCGGCACCTCGTTAAGGAAAGGGGCCTCGATTCCAAATTCTACATTGATTCCGCCGGCACTATCAACTACCACGAGGGTGATCAAGCAGACCCAAGAATGAGGGCATCTTCAAAAAAGCGTGGAATTGAGATAACTTCAATCTCGAGGCCGATAAAGCCATCAGATTTTAGGGATTTTGATCTTATTCTTGCAATGGATAATCAAAACAGAGAGGACATACTAGGTGCATTCGAGAGGTGGAGGCACAGACAGCCGCTACCCGGAAACGCCCATGAAAAGGTTCGCTTGATGTGCTCTTTCTGCAAGAGGCATGATGAAACCGAAGTTCCAGATCCCTACTATGGCGGGCCACAAGGTTTCGAGAAGGTTCTGGATTTACTCCAAGACGCATGCGAAGCTCTATTGGAAAGCATTGTCGCTGAGAAGCTCTAAACTGTCACTTTCCTGATTCACTTGTTTCTTAAATGAAAACACAGCTTATATACTTCTTTTATTTGATAAGCAAATGTGATGCTCTTCCTAAATGCAAATAACTTATTAGCAAACATAAACCAAACAACACAAAATGATAAGGAGGTTGCATCAATGCTATAATCTCAAGTCATTCATGAATCACTAAGCTTTCAAGATATCAATACTTGGATAAGAAAAGCTAGCCTGCAAAGCTCTTCCCGTATTGATCACCACAGCAAACAAGTTCTTATTCCTTCTTTCCAATCCCATGAGGCATATTGTAGATCCCTATCCCCTAAGTCCAAGCGTGAAAATCCCGAAAAATGAAGTTCGCAGTTGCAGAGTTGATCAAATTAATAACAGCCAAGGAGAGAGTTGCGGGTCGACTTTGTTGTTTGGCTGGGCCCGATTGAGG

> NAT0021

CTTCTAGTGTAGTAGATGGTGACAGATAGTATAAGTAGACCAAGTATACCAAGTAATACTCCTGCCAAGTGGTAGAAATAGTATAGGTTGCAGAATTACACCAAAATAGATACTGAAAAATAAACTAAACTACTTCTTAATAACAGCTAAAATAATCAATCAAATTTGAACAACTACATGTTAGACCGTGGCACACTTATGTCAGAGGAGCAAGAGCAGTGGTGAATTATAGTGGCTGAGACGATCCTGCTTTTATGCTTTTAGGTATTAGTATTTATTTGAGTCAGTTATTGTTATTTCTTATTTTCAGTAGTTGGGCTAAGGGCTTGTACGTGTGCATGGGCTTTTTTCCCTGTATAATTAGCAGTAGCATAATTGGAGTTCGATCATCTTGATCAGAATATTGTTTGAGCAGTATTTTGCTGTAGGCCTCGGTTGAGGAGTTCTTGTTATTCAATTATTTTCGTTCTGTTTTCTTCGTGTTCCACCTTTACTCTATCACTACACATGAAGTTCAATTTTAGCTACTGAAACATATATGTATAACATGTGCTTTACATGCTGTCCTAGTGAGATTTGAGTACAAGATTATTTATTCAGTAATTACCTGATATTACATACTTCTGCAGATCAGTAAGCCCCTTTCTCTGTCCTGTAGATCCTGTAAGAAATATAGCAACTGTGAAACCTCGGAAATGCTTCTGCAAGCATAAATATGAAAAGATACGATGAACTGGTTATCAATACGCGCACACACAAATCTGAAAATTTGCCATAACTTAGTGGAAGCTTTTGCTTTCGACATTCTCCATCTTTAAAGAAGGCTACAATACAGTTGCAATCATCTAGACAGCCCTGTTTGCAACGTTCTTCATCAACCCTAAAATTGGTGGTGCAGCCGGCGCTCCATTCGCCTTCATGAGCAAAACCAAATCCCGGAAGGCATCTGCAATCAGCATCTTCATCTTTCTATCTTCTTGCTGCAGTGATCGACTGTACAGTCGAAGAATGCCATCCCAATCAAGCCTCGCAAGATAGATGGTCCCTATTATATGGTTCCCTTGTGTGGTTAAATTCACACGACAGAAAATCCATCGAACAAATAAAGATGACCATCATTTTCAAGATTCAACGTGACGTTGCCATGCCCATAGGTATTTGTTGAATACTCTGGATAGAAGCATCCCACCTGGTGGAAGGCGCTGTCCGGGCAGAAGGGAGTCGGTTGGGTGATCGAAGCTCTGCCAAATTACACTG

> ST0021

ATGGCTGCTCTTCTAGCCTTATTTCTCTTCCTTTCTGCTGCCATTTCAGCAGCAGCTTTAGAAGGATACTCATTGATCAAACTATCATCTTACTTAACACCCACATCCAATTCTTGGGTCTCACATTCTGGAACTTTCGCCTTCGGGTTCCACAACACAGAGGATGGCTACGTCGTCTGCATCTTTTGCGCCGGCTTCAAGGAGAGGACTGTGGTGTGGACAGCCAACAGAGAGAATCCCATTGTTCCTGACGACGTTGTTCTGCTGCTAACTGAAGATGGACTGGTCTTGCAGAGGAGTGATAGCCATGATATAATATCTCGGATTGATAATTCTTCCCAACCTATAGCAGGGGCAGAAATGCATGACAATGGTAACTTTGTGCTCTACAATTCCAATCACAGTGTAATTTGGCAGAGCTTCGATCACCCAACCGACTCCCTTCTGCCCGGACAGCGCCTTCCACCAGGTGGGATGCTTCTATCCAGAGCTTCAGAGACGGACATTAAGACAGGAATTTTCAGGCTCCGGATGCAATATGATGCAAACCTTGTGTTGTACTATGTGTACGGACTAGACACGGCAATAGATGCTTACTATTCAACAAATACCTATGGGCATGGCAACGTCACGTTGAATCTTGAAAATGATGGTCATCTTTATTTGTTCGATGGATTTTCTGTCGTTCGATCACTGCAGCAAGAAGATAGCTGGAAATCAATATGGTCAGTCACGGAGGACAAATGTTTACCTAAAGGTTTGTGTGGCATTAATAGCTACTGCACTTACAAAGATGAAGATGCTGATTGCAGATGCCTTCCGGGATTTGGTTTTGCTCATGAAGGCGAATGGAGCGCCGGCTGCACCACCAATTTTAGGGTTGACTGCAGAGACACATATCTAAGTAGAAAATATGAGATGACAAGCGTTCGGAATGTAACTTGGGAAGACAATTACTACAGTGCTTCAAGAGGACTCACTGAAGAACGTTGCAAACAGGGCTGTCTAGATGATTGCAACTGTATTGTAGCCTTCTTTAAAGATGGAGAATGTCGAAAGCAAAAGCTTCCACTAAGTTATGGCAAATTTTCTGTAAATGCCACAGACATGGCTTTTAAGCATTTCCGAGGTTTCACAGTTGCTATATTTCTTACAGGATCTACAGGACAGAGAAAGGGGCTTACTGATCTGCAGAAGTATGTAATATCAGGAGTATTACTTGGTATACTTGGTCTACTTATACTATCTGTCACCATCTACTACACTAGAAGACAACGTGGATGGTGCAATCAGGGTCTTGACAAATTTGTACTCAAGCATGGATCTCTGGCTCCAAAAAGATATAGTTACAATGAAATAAAAAAGATAACAAAATCCTTTTCAGAAAAACTAGGCCAGGGAGGATATGGCGTTGTATACAAAGGAAAACTGCCTGATGGTCAGCTTGTGGCAGTGAAGGTCTTAAAGAAAACTCATGATAATGCAGAAGAATTTGTTAATGAGGTCGCCAGCATCAGTAGAACCTCCCATGTCAATATTGTCAATCTGTTGGGATTTTGCTATGAGAGAAACAAAAAGGCTCTAGTCTATGAATTCATGCCCAACAAATCTTTAGACAAGTTCATCTACAAGAGTAAATCTGCAGATCCAAATTGCTCCTTGGGATGGAAGAAAATGTACGATATTGCAGTTGGGGTTGCCCGAGGTCTTGAATATCTGCACAGAGGTTGCAACACCAGGATCATTCATTTTGATATCAAGCCTCAGAATATTCTTTTGGACGAAGACTTTTGCCCAAAAATATCAGATTTTGGGCTTGCTAAATTGTGCATGAAGAAGCAGAGCATAATATCGATGCTTGGAACAAGAGGAACTATAGGGTACATTGCTCCTGAAGTATTCTCAAGAAATTTTGGCGTAGTTTCTCACAAATCAGATGTCTACAGCTATGGTATTATGCTTCTTCGAATGGCAGAAACAAGAACAACTGTTGAGACTAAATCATCCCAGTCAAGTGAACATTATTTTCTTGAAGAAATTTACGAGCACGTACTAGTGCAAAATGAAAAGATTCATGATCTCATGACAAAAGAGGAAGAAGAAGTTATAAGGAAAATGTTAATGGTAGGTTTTTGGTGCACTCAGACAGCTCCATTAGATAGGCCGTCAATGAATTCAGTAGTTGATATGTTGCAAGGGAATCTGCAGTCTATACAGGTTCCCCCAATGCCATTTTTGCTCTCTTCCCCAAAACCACACTTCTCGTGCTCACTTCCTCTAATTGTAGAAACGCATTCAAGTTCATATGGTTTAATAAGCCATTATTAA

> NAT0022

TGGTCCACTGTTTCAGTTTGGCCAAGTATAAATAATCTGTGACATTCAGCTTCAATCATTTTAATTAAAAATAAAAAAATCATCAAAGCCCCCAAATTAAGAGTATTCAGTGCATGCTGCTATACGCATGTGGCTTGCGCTGAAATTCGAATAATAAAAAACATAAAATAAGAGAAACATGGCGCTACAGCCAAAGAGCAGAAA

> ST0022

CATTTATTCAACTTTTTACCTATTATATTTAACAAATAAAATACTATCTTTAAAATTCGTGCCTAAAAGAAAAATTGTTCAACCTTTGCGATTATATGTCACGAATTGAAGATGTACAAATTAGGCAGCCATCCGCGCCGGCAAGTAGCACGCCCTTGTATTATATATGAGTGCAAATTCCCACCCCTCTCTCTCTCTCTCTAGACCCTGATACGAGCTAGAAAATGGGAAATTTATTGTGCTGTATTCAAGTGGACCAATCCACCGTTGCGATCAAGGAGCGATTTGGGAAATTTGATGAAGTTCTTGAGCCAGGCTGCCACTGTTTGCCTTGGTTTCTTGGAAGCCAGATGGCTGGCCATCTCTCTCTTCGACTGCAGCAGTTGGATGTCAAGTGTGAGACCAAGACCAAGGACAACGTGTTTGTGAATGTGGTGGCATCGGTGCAGTACCGCGCCATTGCTGATAAGGCTAACGACGCGTTTTACAAGCTCACCAACACAGGAGCCAGATTCAGGCCTATGTTGATCAGAGCCAGTGTCCCAAAACTCAACCTTGATGATGCTTTTGAGCAGAAAAACGACATTGCTAAGGCCGTTGAAGATGAACTTGAGAAGGCTATGTCGGCCTATGGTTATGAGATCGTGCAGACGCTGATTGTTGATATAGAGCCGGACGAGCATGTTAAGAGAGCTATGAATGAGATCAATGCAGCTGCTAGGATGAGGGTGGCTGCAAATGAGAAAGCAGAAGCTGAAAAGATTCTGCAGATCAAGAGAGCCGAGGGCGAGGCAGAGGCCAAGTACCTGGCGGGTGTGGGCATAGCCCGTCAGCGCCAGGCGATTGTGGATGGTCTGAGGGACAGCGTGCTGGGGTTCTCCGTGAACGTGCCTGGGACCACTGCCAAGGATGTCATGGACATGGTCCTCGTCACCCAATACTTTGACACGATGAAGGAGATTGGCGCCCACTCCAAGTCATCCTCCGTCTTCATCCCACACGGACCAGGCCATGTCCGTGATGTGGCAGCCCAGATTCGTGACGGCCTTCTTCAGGCTTCCCAGCTTCCACCTTAACGTCAGCGCTGCATCAAAAGACATTTTCTCGGTGTTCAATTGTATCATCTAGTTCTCTCATTTTGTCGTTACTTGTCTGCATTTTGATATACTGTTTTTATTCCTCTGTTTGAAGAATTGCACAGTGCACATGAGTTTGGTAAAGATATATATTGTACAGTAAGATTGTTCTGTAATATACATAATGTTTCTAGCATAATTTGGTATTTACAATTGCATTGTTTTCTTCTTATTCTGAGCTGCTGCCTGTAAAAGGGGATGATTTTTTTTTTTAATAAAAGTGAAATTTATTCAAAAAGTTCAGGAGAGAGAGGGGATGATTATCTGCTTCAGATTTCAATCTTTTCGTTGGCTATCATTTTTGCAAGATGTCCTTCGAATCTTGAGAAGGAGTCTTCTTCGTCGTGTCTGAATGCGTATCTGTGGTCGTCTCCTCGTCTTCGTCTTCGTCTTGGTCTTCCTTGTCCTCTCCCGCGAGCTCATCCTTCTCTTCCTTTTCAGGCATTTTCTGCTCTTTGGCTGTAGCGCCATGTTTCTCTTATTTTATGTTTTTTATTATTCGAATTTCAGCGCAAGCCACATGCGTATAGCAGCATGCACTGCGAAACGGAAATGGAAATTTTGGGGGCTTTGATGATTTTTTTATTTTTAATT

> NAT0023

ATTATAATTCTCCGTCCGAACTCAAAGAGGTAGATGCTTTCTCATTCTCTCTTCTCACTTCCGTCTCTCGCCGACCCTCAGCAGGCAACGCAGCTGCGCGACTGCACCGCGCTGCCGCCCTAGGCGGTTCGCCCCGCCTGTTCGTCCTCAGCGCCTCCCTCCGCACGCAGCAGCAGCAATTGCCTCCGTCCAAAAGCAGTTCCTAAAATTTGCTGGTTTGAGAGTGAGATCTTGATATCATCATATCAAGATGAAAAAAGTTGCTAAGTCGAAGAAGCACTTAAAAGCAGA

> ST0023

ATGAATTGTTTGATTTTTATGATATTTGTGGCGGCTGTGGGAGTTCCGCCATGTTTTGGACAGAATATCAGTAGAGCAAGCTTCCCAAAGGGCTTTGTTTTTGGGACTGCATCTTCTGCTTATCAGTATGAAGGAGCTGTTAGTGAGGATGGAAGAGGTCCAACCATCTGGGACAAATTTTCCCATGAATTCGGAAAAGTAATCGATTTTAGCAACGCTGACGTAGCAGATGATCAGTACCACCTCTATGATGACGATATACATACAATGAAGGGAATAGGATTGGATGCCTACCGATTTTCCATTGCATGGTCCAGAATCTATCCAAAGGGGTCAGGAGAAATTAACCAAGCTGGAATCGAGCATTACCACAACGTGATCGATGCCTTGTTAGCACAAGGAATCCAACCATACGTGACACTCTTCCACTGGGATCTCCCTCAAAAGCTTGAAGACAAATACAATGGTTTCCTCCATCCTCAAATCATAAAAGACTTCACTGCATATGCTGAGACATGCTTCAAAGAATTTGGAGACAAGGTGAAGCACTGGATCACCTTGAATGAGCCTCACACCTTCGCCGTGCAGGGCTATGATGTGGGGCTGCAGGCGCCCGGGCATTGCTCCATCCTTCTCGGTGCATTCTGTAGGATCGGCAACTCTGCAACCGAGCCCTACATTGTCGGTCATAACATGCTCCTTGCGCACGCCTCTGCAGTCGATGTCTACAAGAAAAAATACCAGCCAAAACAGAAAGGATCAATCGGGATATCATTGGACTCGTTCTGGTACGAGCCAGCATCCGACTCCCCAGAGGACGTCCAAGCCACGCAGAGAGCAATAGACTTCAACTTGGGATGGTTCTTGGAGCCTCTGATCAAAGGGGATTATCCCAAGTCAATGAGAAGCAGAGTCCGTGAGAGGCTGCCAAGGTTCAGCGTGGAGCAGTCCGTGATGCTGAGGGGCTCCCATGACTTCATAGGCATAAATCACTACACAACATGGTATGCACAGCGAGACACCACTAACATCATAGGTGTCTTGCTCAATGACAGTCTTGCAGACTCCGGTGCCTTCACATTCCCCATTGCATTGGGGAAGCCTGTATACGAGAGGGCAAATTCTGTGTGGCTCTACATTGTTCCTCATGGGATAAGAAGCTTGATGAATTACATCAGGAAGGAGTATGGGAACCCCACCGTGATCATCACCGAGAATGGAATGGACGATGGAAACAACCCGCTTACGCCCATCAAGGAAGCTCTCAAGGATGTGAAGCGGATTAAGTATCACCATGACTATTTGACAAACCTGAATGAGGCTATAAAAGACGGTTGCAACGTGAAAGGCTACTTTGTTTGGTCGATGTTGGATAACTGGGAATGGGGAGCTGGATACACTTCAAGATTCGGGCTCTACTACGTCGATTACAAAAACAAGAATCAGAGATATCCCAAGGATTCTGCGACGTGGTTCAAGAATTTTTTGAAATCTTAGTGCAGTTATGGCCACAAAGATGATCAGCCAAGTTGGAAAAATGCAAAACGAGTGCAAGTGATTTGGTATACACGAAATAAAACCATATGTATATGCGGATGGATATCTGATTAAATAAGGGGACACAAGTGGATTTCAAATCTCTTTATATGTATTAGATTAAATATAAACTCGTTCACATAAATGATGACTTGATTTTGGCTCATATATTACAACAATCTTCTAACATCAAAGTTACAAGAAAGAAGAGTCGACTCAAAAATTTAAGCAGCTAACATCAGACAAAATTGTGATTGAGTCTATCAAAAACAGCAGTCGCAATCAAAGGGACACAAAGCTGTTCCTCCTGCATCTGGCTTCCCTATGCCCAGCTCTTCTTCCGAAAAAACAGACAGCCCGTCGGCAGTTTTCTTCCTCGGCCAAGAAGTGCCAACTGCAGATGAACTTTGTTTGAAGGAGCGACTATTGCTACTCTTTCCCTTCTTTTTGTCAAGTGCATTTGCAGCAGCTACTTTGGCTGGCTTCTCATCCTTGGCCTTCTTTTCCGTTTCCAGTCTCTTGCGTTTCTTTCCAGCAAAGATTTCATCAATCTCACTTCCCTTCTTTTTTGGTTGGACAGAGGCCTTCGCCTGTTCCACAGCATTACTCTCGTGTTCTGCTTTTAAGTGCTTCTTCGACTTAGCAACTTTTTTCATCTTGATATGATGATATCAAGATCTCACTCTCAAACCAGCAAATTTTAGGAACTGATTCAGAAAAAGAAAAACGTAATTTGAAGAATACCAAGGGGATGAGATGCAAAAAAAGATGAGAAATCAGGTGCAAAATATCAAACAGCATCTATAAATACTAGTATAAATAGTGATAGTTTAACTACAACGCAATTGTTGTGGCCTTAGATCATCTCGTAAACTAATTTGAGGACTTAAGGCTCATTCTTTTTTACGTTGTTTTTAGTTGTTCAGCTGTGAATTGAATCGAATAGGGACCTAGTAACCTTACCTCCTTCAGATTTGCTTGCATCACTTATACTTATAATACTAGTGTTGTATACATATTTCTTAATCTACGTGCCGAAAAAAAAAATGAGACATTTCGTTTGGGACGGAGGGAGTACTATATATAAAAATATTACATTATAAGTTCACTAATCAATAATGATTTAATCAAGCTGAGTACTGTAGCTTGGTAGTATTCCTTTATTTATAATAGTTTCATACCCCAATTAAAAAAAAAATAGATTTAATCAAACACATTGTAAGATAATTGAGTCATACTATTGAACAATCAACCAAGAAGTATTATATATTTCATCAATTATAATTACTAGAGAATAAGTCCAGTTTAAGATATGAGAAAAAAAAACACACACACAGACACGCACAATATTAATTACTCACACAACATGAATTCTTTAACACTATCAGAGGTTTCATTACAAATACAACGAACAAGACAAACTAAAGAATTAATGCTGGCATCAGTTGTTTTATCAGGCATAAACACTCTACAAAGCATGCTAACAACAGATAAAGATAAACAAAAATAAGATTTTTCTCAATTCTCAGGCATAACACTGTAAGTCGGCCAAGTAGTAGACAGCACTCTATAAAGTACAAAGCATTCTAACATCAGATAAAGAAAAACAAAATAAAAACCAGTGTAACAGCAATTGAAAATTTAACCAAATAATCCATAGTCTAGGTATGCTTGACTCTTTATTGCCGAATTTTATAGGGATTGAGATGCAAGAAAAGCAGATGCCCCACCCCGATTTTACCCATAAACCCCTACCCGACCCCCACTTGGGATTAAGATAAATACCTCTTAAATTTAGGACTCCAAAACATTAGGGGTAGGGCAGCGGCGGCTGCCGCCTTACCTGCTTTTGGACGGAGGCAATTGCTGCTGCTGCGTGC

> NAT0024

ATCGCGGACGGAGGAAGTATAAAATTATATCTGTAGTGAAACAATCTACTGAGTAGAACAGTTGCATGCAACAACAACCTAATTAGCCAGCTAACCATCTCTGAAAGCATCACATGGAAAGAATGAATCTAGTTTCCACAAATAAATCAGAGAGAGAGAGAGAGAGAGCAGGAGAAGCTGAGCTCCTGCATTCCGGAATATGTTTCTGAAACTTTCATCACCAAATAGAACCG

> ST0024

ATGGCTTATGAAGCTCTTGATACTCTGTTTCAAACCTTAGATCGAATCCTAAAGCATGGTGATAATCGCGTCATCACTCATTCTGTAAAGCAAGAAATTTTATCCATCCGCGTCCAAGCTATTGTCTTGCAGTTGAATCTCAAACACTATCCAGACAAAGAAAGAATCAGAGAGGCAGCAATTTCAACA

CATGAGATTACTGAATATCTCTTCTCTGAAGAAAACCTTTCAGATGTTGGATGGATAGATCCGACAGTCATACTTGAAAACCGGCTGAGGGAACTAGCACAGAGATTGGAGTCAACCGTTGGAGATGTGGTGGATTGCATCAAGGGTAACGACTCGGTCAGCAGCGTACAACGACTGAGTACTGATTCCCCTGATGTTAGTTCATCATCAAGATCAGCAATTACAAGAAGCAAAGATGACGACGTAGTTGGTTTGGAAGAGGATGTGGAATTGATCAAGGGTCGGCTTTGCAAGGGAACATCCAAACTACAGGTCCTCCCAATTGTTGGAACAGGAGGCATTGGTAAGACCACACTTGCTAAAATTGTTTATAATGATGAATTGATTAGGAAAAAATTTAGTACTTGTGGTTGGGTCACAATATCACAAGATCATAGTGTAGAACGAATTGTTCTAAATCTTCTAGCTTCCATAAAAGAAATTTCAGCACTAAGAGATGCTAAGAGCGACATCCCTAAAGAAACCTTAATTTGCGAGTACCTAAAAGGTCGAAGGTATCTTATTGTAATGGACGACATGTGGAGTAAGAAAGCCTGGGATGGTGTAAAGATGTTGTTTCCTGATGTCGGTCATGGCAGTCGGATCATAGTCACCACAAGGTTGCAAGATGTGGCTGCTTATGTCGACTCTTCTAAGTCCATTCATACTATGAGTTTGTTGGATGCACATCATAGCTGGAATTTGCTAAAGCAGAAGGTGTTCGGATATAAAGACATTCCTATTGAACTAGAAGACATTGGAAAGAAAATTGTTAAAAATTGTGGAGGGTTGCCCCTCTCAATTGTTGTGGTGGCCGGACTTCTATCTAAGATCCATACTCGATACTCATGGAAGCAAATTGCTGCGAATGGCGGGCAGCTTGAAACAATAATTGGTTTGAGTTATACCCATTTACCCAATCACTCAAAGCCGTGTCTTTTATATATGGCTGGCTTTCCAGAAGATTATGAGATTCGTGTCTCAGAACTTATTCACCTTTGGTTATCTGAAGGTTTTGTAACACTTTCAAATGAACCTAAAAGCTTGGAAGAAGAAGCAGAAGATTGTTTCGAGGATCTTGTCGAAAGAAGTTTAGTTTTGGTCACCAACAGGAAGTTTGGTGGGAAAATAAAGAGTTGCAGTCTTCATGATATTGTGCGGGAATTTCTTGTAAGGCAGGCTGCAAAAGAGAAGGTAATTCTTTCTGTTATGGATTACTTACCTACTCCTATCCTGCGAAAGCATTTTGTTCCACGTCTCATAAAAGATCATCATAGCATAAGTGCTAGTTCGTATGATCTACACCTCAAAGACTATGTGCATAGCTCACACATCCGTACCATAATATGTATTCCGAAGAAAGGGTATAGATCCGAGGGCGTCGTAGAGAAGTTTAGTTCACTGAGGGTCCTTCATGTTTTACGCAGAAACAATCATTGGGATTGGGAGCCTGGTCAAGTGTTTGATCTAGTTCATTTAACTTACCTTGCTTCCAACATTCCCAATAGTATTGTTCCTTCGGCCATATCAAAGCTTGAGAATGTTCAGACTTTAATTATTTATAGATCCGAGGTTCGTTTGCCCATTGAGATTTGGGGGTTGCGGCAATTAAGACATCTTATTGCCTTCTCATTTCAACCCTTACCCAACCCTGAAGGGGAACAAAATCCCCTTGACAACTTGGAGAAACTTTCGTTGGCAAGGGACTTGGTGTGCACTAAAAGAATGGTGAAAATGATTCCAAACATTAAAAATCTGGGAATATGTTACTCCATGGAGAAGTTTGATGTGGATGCTGGCTATCGCCTCAACAATCTTAAGTATCTATGTCGACTGGAGAAATTGAAGTTGGAGGTGCATGGAGGTATTTTATCCAGTCAACTATGTGATGAGATGGCATTGGGGATGCTTGGAGGTTTTTCATTCAGACTAGTTGATAAGATACTTGGATGGATTTCATTTAGACGAGCTAATAAGATACTTGGAGGTATAAACTTTCCTCTACAACTGAGAAGGTTAACCTTGAGTGGTTTGAAGCTTCCTTCGAGTGATATGACAATCGTTGGTTCATTACCTAATCTTCAAGTGCTTAAACTAAGAAACTTTGCTTGCAAAAGCATAGATTGGGTAACATCTAAGGGAGAATTTCCTGAGCTCAGACTTCTGCTAATTGACCAATCACATTTGGTTTTTTGGATAACAGAAGCTAGCCACTTTCCGAGACTCGAGTGCCTAATGCTTCATGGGTGTCTTGGTCTACGTGAGATTCCAAGTAATATTGGAGACATTCCAACACTTAAATTGATTGAGGTCGATGAGCAGTCCACGGGTCTTTTATTCTCGGCAGAAAAGATACAAAATAAACAAAAGAATTCTGGAAAGCAGTTTCTAAATGTTGATGACAATAACTTGGCTGACATGGATGATGACTATATGCCCGATGCTGAAGACACACGACATAATGAGAACACCGGGTGGTCTTCTTGTACAAGGGGCAGAGTGTGGAAGGAATGCTCTCTCCTTGGACAACCTCTTAATTGGCAAATCACGCAAGCCGCAAGGGAAGCATCGAGGATGTTTTTTGTAACACTGGTCCTGAAAACAAGAGACTGCATCCGTGTGGAAGAGCGGATTCCATTTGGCGATAAGACCACAAACAAGGCTGATGAAATCGGACTTGTAAACCACATCTGTGTGGAAGAGCGGTTCTATTTGGTGATGAAAGTTTCAGAAACATATTCCGGAATGCAGGAGCTCAGCTTCTCCTGCTCTCTCTCTCTCTCTCTCTGA

> NAT0025

AGCTCGTCTGTCATCTCATTGAGCTCTTCTGTCCTCTCATTCAGCACTTCCTCTAGCCGTCTAACCTTATCAGCAAGATCCACCCCCCTCTGCATAACATAAATGTCACAGCAGTAGTGAAAATGTACACTTCATTTATATATGTAGGAAACAGCTACAACTTTTACTTGTTTGGCCAGCTCCCAGACAGATACGTATGCAATTTGCTCAACCAAAACAGCAGACAAGAAAAGAAGTAATGATGATGTGCTAAGGTTCAAAATACCTAACCTCTGTGCTATCTTCATCTAGTCTCGGCCTCTTGAGCCTCTTTCCATCTTCGCTCCAAGAAAGTCGATCAGCTTCTGCTTTCACTTCCTCGCCAAGGTCAATTGCCAAGAATCTAGCCATGCCAAAATGATTTGCTCTGTGCTTCCCACTTTCCTCGCTCTCGGCCACTCGAATAGCCATCGGAGCCCCTTAATTTGTATGTTCGATACTGAAAAAGCTTAT

> ST0025

CGAAAATTTCCTTTTCCTTTTACAGTTCGAAGCAGTAAATGCAGGAAGGCAAACGAACTTGTTTTTAGTGAAGAGAAAGCTACTATTTCACTTCTTCACCTCCCAATCTCTTCTTCTGTATCTTCTGCAGATCCAATTTACTCATCTTATTTAAGATTAGGGATCTCCGTATCCACTAGCTTACAAATTCCTTCGGACGCACATACGATTCCGTCGATGCGGCGGCTTCCCTTGCCCCTGGGTATATCTAAGATTCAAATGAAGAAACCAATTGAAACGTTCCAACTATGAACCAAAAAAGCTCAAGAGTTGATGTTGAGGTCGGAAATAGTTCAACCGACAACTTTGACGACCTCAATGACGCACAAGTTACCGATTATAAACAACTTAGTTATAAGCTTTTTCAGTATCGAACATACAAATTAAGGGGCTCCGATGGTGAGTTTGGATGCCCCTTTTGTGATGATTACAAAGATGAGCATGAATGTAAGTACACTCATCTTCCTTTACATGCTATTCGAGTGGCCGAGAGCGAGGAAAGTGGGAAGCACAGAGCAAATCATTTTGGCATGGCTAGATTCTTGGCAATTGACCTTGGCGAGGAAGTGAAAGCAGAAGCTGATCGACTTTCTTGGAGCGAAGATGGAAAGAGGCTCAAGAGGCCGAGACTAGATGAAGATAGCACAGAGAGGGGGGTGGATCTTGCTGATAAGGTTAGACGGCTAGAGGAAGTGCTGAATGAGAGGACAGAAGAGCTCAATGAGATGACAGACGAGCTGAATGATTATATTACAAGAGAGCGCCAAAGCAATAATGAGTTGCATGGGGTTCGCAGAGAACTGATACAGGGCTTGAATGATATTCTCAAGGGAAATCATGTTCACATTGGGATAAAGAATATGGGAGAAATTGATCCCAAAGTCTTCATCAGTGAAATGGAAAAGAGATTACCCCCCATAGATGCTGAGATGAAAGGGGTTGAGTTGTGTTCCTTGTGGCAGGAAAAGATTAAAAATTCTCAGTGGTATCCGTATCAAATCATTGTAGATGATAATGGGAAACCTAAGAGAACACTGAAGGAGGATGACACATCACTTCAAGGCCTCAAGAAACAATGGGGCCAAGAAATTCATGATGCAGTTGTTGGAGCTCTGAGTGACCTCTACGACTACAATCCAAGTGGATGTTATGTGGTTCCTGAGCTGTGGAACTTCAAAGAAAATAGGAAGGCGAAGCTTGATGAAGTCATTAGCTACATCCTTGAAGCTGTTAAAGAGCATGGAAAGATAGGAAGGTGTTTGGGTAATCATTCAGATGGCATAGCCAGCATTAGTGATTCTGACGTCTATGAATACAAAGGAAGAATTTATGAGCTATTGAAATTATCAATCCCTTGCAAAGTGAGGGGACCTAGTGGTGAACTTAGATGCCCTTTCTGTTCTAATGAGGAAAATCGAGAGTACAACTATCTTCTTCGACATGCTCTTGCGGTAGGTGAGAGCTCCTCTGAGAGTGCAAAGCAGCGAGCAAAGCATTTTGCATTGGCTAAATACTTGGTTATTGACCTACAACATAATGTGGCACAAAGCACAGCATCTTCACCTGCTTCCTCAAGTGGCGGAGACCAGGAAATTGGGGAAGGGGTTCAATCAGAAGCGCAGCTCAATGATGCAACGATAAATGATGAGAAGTTCCCAGTAGTAAATGATTTGAAGAAATTGTCGAGGCAAATGATTTCTCAGTATTTCTGCATGCCAATTGCTCAAGCAGCAAAGCAGCTCAATATAGGGTTAACCTGCTTGAAGAGAAGGTGTAGGGAGTTGGGAATCCCGAGATGGCCATACAGAAAAAGGCGCAGCCTTCAAATCCGAAACAAGGATGTTCAGGCCAAGGCCAACAAGAATGTTCAGGTGGAGACCAACTCGGAGTCCGAGTCAGAGTGCGAGTTTGAGTCCGACGATGAAGATGATTTGCCATTAAGTAGGAAGTATGCAAGGAAGAAGTGATAATTTGACTACTTGTTTGTATGTAGAAGGTAAGTTGCAGGAATCTTTGTAACAATACAAGAAATTACTCACTCCGTCCTAATATTCATGATTCGTATTTTTTTTTTGAGTCGTCCATGTTACTTGACTCGTTTCCTTTTTTAGATGTAATTAGTGAACAATTAAATGTATAATTAATCCTAGTATTTAAACCTAAAATCTAACCTTAAAAATACC
