# Supplementary material for: Genome-wide identification and functional characterization of natural antisense transcripts in Salvia miltiorrhiza
Source: Sci Rep. 2021 Feb 26;11:4769. doi: 10.1038/s41598-021-83520-6 (PMC7910453; doi:10.1038/s41598-021-83520-6)
Supplement: Supplementary file 3 — Supplementary Information. [file 41598_2021_83520_MOESM3_ESM.pdf]

COVID-19 is an emerging, rapidly evolving situation.
Get the latest public health information from CDC: https://www.coronavirus.gov .
Get the latest research from NIH: https://www.nih.gov/coronavirus.
Find NCBI SARS-CoV-2 literature, sequence, and clinical content: https://www.ncbi.nlm.nih.gov/sars-cov-2/.

**Job Title**	ST0001 ...
**RID**	NWB01UZB016 Search expires on 09-14 22:26 pm
**Program**	BLASTP
**Database**	swissprot
**Query ID**	lcl|Query_31394
**Description**	None ...
**Molecule type**	amino acid
**Query Length**	595

Descriptions

Description	Max Score	Total Score	Query Cover	E value	Per. Ident	Accession
RecName: Full=Miltiradiene synthase KSL1, chloroplastic; AltName: Full=Kaurene synthase-like 1; Short=SmKSL; Short=SmKSL1; Flags: Precursor [Salvia miltiorrhiza]	1233	1233	100%	0.0	99.16%	C8XPS0.1
RecName: Full=Class I diterpene synthase 2, chloroplastic; Short=VacTPS2; AltName: Full=(13S)-vitexifolin A synthase; AltName: Full=9,13-epoxylabda-14-ene synthase; AltName: Full=Viteagnusin D synthase; Flags: Precursor [Vitex agnus-castus]	826	826	99%	0.0	67.23%	A0A2K9RFZ2.1
RecName: Full=Class I diterpene synthase TPS6, chloroplastic; AltName: Full=Labd-13(16),14-diene-9-ol synthase; AltName: Full=Syn-isopimara-7,15-diene synthase; AltName: Full=Terpene synthase 6; Short=VacTPS6 [Vitex agnus-castus]	795	795	99%	0.0	63.41%	A0A2K9RFZ9.1
RecName: Full=9,13-epoxylabda-14-ene synthase, chloroplastic; AltName: Full=Manoyl oxide synthase; AltName: Full=Miltiradiene synthase; Flags: Precursor [Marrubium vulgare]	766	766	99%	0.0	64.37%	A0A075FBG7.1
RecName: Full=Cis-abienol synthase, chloroplastic; Short=NtABS; Flags: Precursor [Nicotiana tabacum]	371	371	84%	6e-118	37.40%	G3CCC1.1
RecName: Full=Santalene and bergamotene synthase, chloroplastic; AltName: Full=(+)-alpha-santalene synthase ((2Z,6Z)-farnesyl diphosphate cyclizing); AltName: Full=(+)-endo-beta-bergamotene synthase ((2Z,6Z)-farnesyl diphosphate cyclizing); AltName: Full=(-)-endo-alpha-bergamotene synthase ((2Z,6Z)-farnesyl diphosphate cyclizing); Flags: Precursor [Solanum habrochaites]	351	351	84%	2e-110	37.20%	B8XA41.1
RecName: Full=Beta-phellandrene synthase (neryl-diphosphate-cyclizing), chloroplastic; Flags: Precursor [Solanum lycopersicum]	346	346	84%	1e-108	37.01%	C1K5M3.1
RecName: Full=Ent-kaurene synthase-like 2; Short=OsKSL2 [Oryza sativa Japonica Group]	316	316	83%	8e-96	34.13%	Q0JA81.2
RecName: Full=Ent-kaur-16-ene synthase, chloroplastic; AltName: Full=Ent-kaurene synthase; AltName: Full=Ent-kaurene synthase B; Short=KSB; Flags: Precursor [Cucurbita maxima]	309	309	92%	3e-94	33.27%	Q39548.1
RecName: Full=Ent-kaur-16-ene synthase, chloroplastic; AltName: Full=Ent-kaurene synthase; Short=AtKS; AltName: Full=Ent-kaurene synthase B; Short=KSB; AltName: Full=Protein GA REQUIRING 2; Flags: Precursor [Arabidopsis thaliana]	305	305	86%	8e-93	35.23%	Q9SAK2.1

Graphic Summary

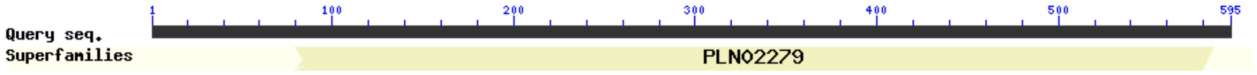

Distribution of the top 10 Blast Hits on 10 subject sequences

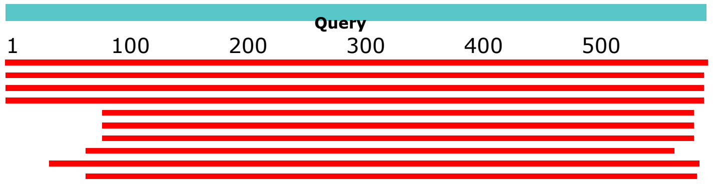

Alignments

Alignment view Pairwise ☐ CDS feature Restore defaults

RecName: Full=Miltiradiene synthase KSL1, chloroplastic; AltName: Full=Kaurene synthase-like 1; Short=SmKSL; Short=SmKSL1; Flags: Precursor [Salvia miltiorrhiza]

Sequence ID: **C8XPS0.1** Length: 595 Number of Matches: 1
Range 1: 1 to 595

Score	Expect	Method	Identities	Positives	Gaps	Frame
1233 bits(3190)	0.0()	Compositional matrix adjust.	590/595(99%)	592/595(99%)	0/595(0%)	

```
Query  1    MSLAFNPAATAFSGNGARSRRENFPVKHVTVRGFPMITNKSSFAVKCNLTTTDLMGKIAE  60
            MSLAFNPAATAFSGNGARSRRENFPVKHVTVRGFPMITNKSSFAVKCNLTTTDLMGKIAE
Sbjct  1    MSLAFNPAATAFSGNGARSRRENFPVKHVTVRGFPMITNKSSFAVKCNLTTTDLMGKIAE  60

Query  61   KFKGEDSNFPAAAAVQPAADMPSNLCIIDTLQRLGVDRYFRSEIDTILEDTYRLWQRKER  120
```

```
              KFKGEDSNFPAAAAVQPAADMPSNLCIIDTLQRLGVDRYFRSEIDTILEDTYRLWQRKER
Sbjct  61    KFKGEDSNFPAAAAVQPAADMPSNLCIIDTLQRLGVDRYFRSEIDTILEDTYRLWQRKER  120

Query  121   AIFSDTTIHAMAFRLLRVKGYEVSSEELAPYADQEHVDLQTIEVATVIELYRAAQERTGE  180
              AIFSDT IHAMAFRLLRVKGYEVSSEELAPYADQEHVDLQTIEVATVIELYRAAQERTGE
Sbjct  121   AIFSDTAIHAMAFRLLRVKGYEVSSEELAPYADQEHVDLQTIEVATVIELYRAAQERTGE  180

Query  181   DESSLKKLHAWTTTFLKQKLLTNSIPDKKLHKLVEYYLKNYHGILDRMGVRQNLDLYDIS  240
              DESSLKKLHAWTTTFLKQKLLTNSIPDKKLHKLVEYYLKN HGILDRMGVRQNLDLYDIS
Sbjct  181   DESSLKKLHAWTTTFLKQKLLTNSIPDKKLHKLVEYYLKNXHGILDRMGVRQNLDLYDIS  240

Query  241   YYRTAKAANRFSNLCSEDFLAFARQDFNICQAQHQKELQQLQRWYADCKLDTLKYGRDVV  300
              YYRT+KAANRFSNLCSEDFLAFARQDFNICQAQHQKELQQLQRWYADCKLDTLKYGRDVV
Sbjct  241   YYRTSKAANRFSNLCSEDFLAFARQDFNICQAQHQKELQQLQRWYADCKLDTLKYGRDVV  300

Query  301   RVANFLTSAIIGDPELSDVRIVFAQHIVLVTRIDDFFDHRGSREESYKILELIKEWKEKP  360
              RVANFLTSAIIGDPELSDVRIVFAQHIVLVTRIDDFFDHRGSREESYKILELIKEWKEKP
Sbjct  301   RVANFLTSAIIGDPELSDVRIVFAQHIVLVTRIDDFFDHRGSREESYKILELIKEWKEKP  360

Query  361   AAEYGSEEVEILFTAVYNTVNELAERAHVEHGRSVKDFLIKLWVQILSIFKRELDTWSDD  420
              AAEYGSEEVEILFTAVYNTVNELAERAHVE GRSVKDFLIKLWVQILSIFKRELDTWSDD
Sbjct  361   AAEYGSEEVEILFTAVYNTVNELAERAHVEQGRSVKDFLIKLWVQILSIFKRELDTWSDD  420

Query  421   TALTLDDYLSASWVSIGCRICILMSMQFIGIKLSDEMLLSEECIDLCRHVSMVDRLLNDV  480
              TALTLDDYLSASWVSIGCRICILMSMQFIGIKLSDEMLLSEECIDLCRHVSMVDRLLNDV
Sbjct  421   TALTLDDYLSASWVSIGCRICILMSMQFIGIKLSDEMLLSEECIDLCRHVSMVDRLLNDV  480

Query  481   QTFEKERKENTGNSVTLLLAANKDDSSFTEEEAIRIAKEMAECNRRQLMQIVYKTGTIFP  540
              QTFEKERKENTGNSVTLLLAANKDDSSFTEEEAIRIAKEMAECNRRQLMQIVYKTGTIFP
Sbjct  481   QTFEKERKENTGNSVTLLLAANKDDSSFTEEEAIRIAKEMAECNRRQLMQIVYKTGTIFP  540

Query  541   RQCKDMFLKVCRIGCYLYASGDEFTSPQQMMEDMKSLVYEPLTIHPLVANNVRGE  595
              RQCKDMFLKVCRIGCYLYASGDEFTSPQQMMEDMKSLVYEPLTIHPLVANNVRG+
Sbjct  541   RQCKDMFLKVCRIGCYLYASGDEFTSPQQMMEDMKSLVYEPLTIHPLVANNVRGK  595
```

RecName: Full=Class I diterpene synthase 2, chloroplastic; Short=VacTPS2; AltName: Full=(13S)-vitexifolin A synthase; AltName: Full=9,13-epoxylabda-14-ene synthase; AltName: Full=Viteagnusin D synthase; Flags: Precursor [Vitex agnus-castus]

Sequence ID: **A0A2K9RFZ2.1** Length: 589 Number of Matches: 1

Range 1: 1 to 584

Score	Expect	Method	Identities	Positives	Gaps	Frame
826 bits(2133)	0.0()	Compositional matrix adjust.	398/592(67%)	487/592(82%)	9/592(1%)	

```
Query  1     MSLAFNPAATAFSGNGARSRRENFPVKHVTVRGFPMITNKSSFAVKCNL-TTTDLMGKIA  59
             MSL FN   T FS +  R+RRE FP +      FP+ T+KS+  VKCNL T+TDL+GK+
Sbjct  1     MSLRFNLIVTPFSNHRIRNRRETFPAQE-----FPVATSKSAVKVKCNLITSTDLVGKVR  55

Query  60    EKFKGEDSNFPAAAAVQPAADMPSNLCIIDTLQRLGVDRYFRSEIDTILEDTYRLWQRKE  119
             EK  G+  N     A+ P  D+PSNLC+IDTL+RLGVDRYF+SEID +LE+TYRLWQ+KE
Sbjct  56    EKINGKVDNSLEVPAIHPV-DIPSNLCMIDTLERLGVDRYFQSEIDGVLEETYRLWQQKE  114

Query  120   RAIFSDTTIHAMAFRLLRVKGYEVSSEELAPYADQEHVDLQTIEVATVIELYRAAQERTG  179
             + IF+D T  AMAFRLLRVKGYEVSS+ELAPYADQ HV+LQ  +V  VIELYRA+QER
Sbjct  115   KDIFADVTCRAMAFRLLRVKGYEVSSDELAPYADQAHVNLQISDVTAVIELYRASQERIY  174

Query  180   EDESSLKKLHAWTTTFLKQKLLTNSIPDKKLHKLVEYYLKNYHGILDRMGVRQNLDLYDI  239
             E+ES+L+KLHAWT+T+LKQ+L++ +I DKKLHK VEYYLKNYHGILD +G+R++LDLYDI
Sbjct  175   EEESTLEKLHAWTSTYLKQQLVSGTISDKKLHKQVEYYLKNYHGILDLVGIRRSLDLYDI  234

Query  240   SYYRTAKAANRFSNLCSEDFLAFARQDFNICQAQHQKELQQLQRWYADCKLDTLKYGRDV  299
              +Y+  KAA+RF  +C +D LAF+RQDFN CQAQ+Q+ELQ LQRWY DC+LD L YGRDV
Sbjct  235   DHYQILKAADRFRTIC-KDLLAFSRQDFNNCQAQYQRELQLLQRWYEDCRLDKLNYGRDV  293

Query  300   VRVANFLTSAIIGDPELSDVRIVFAQHIVLVTRIDDFFDHRGSREESYKILELIKEWKEK  359
             +R++ F++SAIIGDPELSD R+ FA++ VL T IDDFFDH GSREESY+ILEL+KEWKEK
Sbjct  294   LRISYFVSSAIIGDPELSDARLAFAKYCVLTTCIDDFFDHAGSREESYRILELVKEWKEK  353

Query  360   PAAEYGSEEVEILFTAVYNTVNELAERAHVEHGRSVKDFLIKLWVQILSIFKRELDTWSD  419
             PA +YGS+EVE LFTAVYNTVNELAE A+VE GR VK  LIKLWV++L+ FK+ELD+W+D
Sbjct  354   PAEDYGSKEVEFLFTAVYNTVNELAEMAYVEQGRCVKSLLIKLWVELLTSFKKELDSWTD  413

Query  420   DTALTLDDYLSASWVSIGCRICILMSMQFIGIKLSDEMLLSEECIDLCRHVSMVDRLLND  479
             DTAL+LD+YLS+SWVSI  RI IL S+QF+G+KLS+EML S+EC DLCRH S+V RLLND
Sbjct  414   DTALSLDEYLSSSWVSITSRINILTSIQFLGLKLSEEMLSSQECTDLCRHGSLVVRLLND  473

Query  480   VQTFEKERKENTGNSVTLLLAANKDDSSFTEEEAIRIAKEMAECNRRQLMQIVYKTGTIF  539
             +QTFEKER+ENT NSV++LL A K + + TEEE I   KE+ E NRR+LMQ+VY+ GTIF
Sbjct  474   MQTFEKERRENTKNSVSILLEAPKHEGAITEEEVISKIKEIVEQNRRKLMQMVYQRGTIF  533

Query  540   PRQCKDMFLKVCRIGCYLYASGDEFTSPQQMMEDMKSLVYEPLTIHPLVANN  591
             PR+CKD+FLK CR G YLY++GDEFTSP Q+MEDMK L YEPLT HPL ANN
Sbjct  534   PRKCKDVFLKSCRGGYYLYSNGDEFTSPVQIMEDMK-LCYEPLTFHPLEANN  584
```

RecName: Full=Class I diterpene synthase TPS6, chloroplastic; AltName: Full=Labd-13(16),14-diene-9-ol synthase; AltName: Full=Syn-isopimara-7,15-diene synthase; AltName: Full=Terpene synthase 6; Short=VacTPS6 [Vitex agnus-castus]

Sequence ID: **A0A2K9RFZ9.1** Length: 594 Number of Matches: 1

Range 1: 1 to 588

Score	Expect	Method	Identities	Positives	Gaps	Frame
795 bits(2053)	0.0()	Compositional matrix adjust.	376/593(63%)	478/593(80%)	6/593(1%)	

```
Query  1     MSLAFNPAATAFSGNGARSRRENFPVKHVTVRGFPMITNKSSFAVKCNL-TTTDLMGKIA  59
             MSL FN   T FS    R+RRE FPV+      F M T+KS+  VKC+L T+ DL+GKI
Sbjct  1     MSLRFNLIVTPFSNYEIRNRRETFPVQ-----KFLMTTSKSAIKVKCSLKTSIDLVGKIR  55

Query  60    EKFKGEDSNFPAAAAVQPAADMPSNLCIIDTLQRLGVDRYFRSEIDTILEDTYRLWQRKE  119
             EK  G+  N      +    D+PSNLC+ID+L+RLGV RYF+SEID +LE TYRLWQ++E
Sbjct  56    EKINGKVDNSLEVPTINYLVDIPSNLCMIDSLERLGVARYFQSEIDGVLEKTYRLWQQRE  115

Query  120   RAIFSDTTIHAMAFRLLRVKGYEVSSEELAPYADQEHVDLQTIEVATVIELYRAAQERTG  179
             + IF+D T  AMAFR LRVKGYEVSS+ELAPYADQ HV+ Q  +V TV+ELYRA+Q R
Sbjct  116   KDIFADVTCRAMAFRFLRVKGYEVSSDELAPYADQVHVNPQISDVTTVVELYRASQVRIY  175

Query  180   EDESSLKKLHAWTTTFLKQKLLTNSIPDKKLHKLVEYYLKNYHGILDRMGVRQNLDLYDI  239
             E++S L+KLHAWT+TFLKQ+L + +I DKKLH+ VEYYLKNYHGI +++ VR++LDLYDI
Sbjct  176   EEDSILEKLHAWTSTFLKQQLQSKTISDKKLHEQVEYYLKNYHGIQNQVAVRRSLDLYDI  235

Query  240   SYYRTAKAANRFSNLCSEDFLAFARQDFNICQAQHQKELQQLQRWYADCKLDTLKYGRDV  299
              +Y   K A+RF  + +EDF  F RQDFN+CQAQHQKELQQLQRWY DC+LDTL YGR+V
```

```
Sbjct  236   DHYPILKVADRFRIIYNEDFFVFLRQDFNLCQAQHQKELQQLQRWYEDCRLDTLNYGRNV  295

Query  300   VRVANFLTSAIIGDPELSDVRIVFAQHIVLVTRIDDFFDHRGSREESYKILELIKEWKEK  359
             V V+ FL +A  GDPELS+ R+ FA+ IVLVTRIDDFFD  GSREESYKILEL+KEWKEK
Sbjct  296   VHVSCFLAAANFGDPELSNARLAFAKTIVLVTRIDDFFDLAGSREESYKILELVKEWKEK  355

Query  360   PAAEYGSEEVEILFTAVYNTVNELAERAHVEHGRSVKDFLIKLWVQILSIFKRELDTWSD  419
             P  +YGS+EVEILFTA+Y+TVNE AE A++E GR VK  LIKLWV++L+ FK+ELD+W+D
Sbjct  356   PTEDYGSKEVEILFTALYDTVNEFAEIAYIEQGRCVKPLLIKLWVELLTSFKKELDSWTD  415

Query  420   DTALTLDDYLSASWVSIGCRICILMSMQFIGIKLSDEMLLSEECIDLCRHVSMVDRLLND  479
             DTALTLD+YLS++W+SI CR+C L ++QF+G+KLS+EML S+EC DLCRH+S V+RLLND
Sbjct  416   DTALTLDEYLSSAWMSIACRVCTLTALQFLGVKLSEEMLSSQECTDLCRHLSFVNRLLND  475

Query  480   VQTFEKERKENTGNSVTLLLAANKDDSSFTEEEAIRIAKEMAECNRRQLMQIVYKTGTIF  539
             VQTFE+ERKENT N+V++LLAA++ + + TEEEAI   +E+ E NRR+LM++VY+  ++F
Sbjct  476   VQTFERERKENTINAVSVLLAAHRHERAITEEEAISKIQEIVEQNRRKLMRMVYQRESVF  535

Query  540   PRQCKDMFLKVCRIGCYLYASGDEFTSPQQMMEDMKSLVYEPLTIHPLVANNV  592
             PR+C+++FL+V ++G YLYASGDE T+PQQ+MEDMKSLV+EPL +HPL  NNV
Sbjct  536   PRKCRNVFLEVSKMGHYLYASGDELTTPQQLMEDMKSLVFEPLALHPLETNNV  588
```

RecName: Full=9,13-epoxylabda-14-ene synthase, chloroplastic; AltName: Full=Manoyl oxide synthase; AltName: Full=Miltiradiene synthase; Flags: Precursor [Marrubium vulgare]

Sequence ID: **A0A075FBG7.1** Length: 580 Number of Matches: 1

Range 1: 1 to 580

Score	Expect	Method	Identities	Positives	Gaps	Frame
766 bits(1978)	0.0()	Compositional matrix adjust.	383/595(64%)	471/595(79%)	18/595(3%)	

```
Query  1     MSLAFNPAATAFSGNGARSRRENFPVKHVTVRGFPMITNKSSFAVKCNLT-TTDLMGKIA  59
             MS+ FN     FSG G +  +E FP    + +       + KS+   KC+   +TD MGK+
Sbjct  1     MSITFNLKIAPFSGPGIQRSKETFPATEIQITA----STKSTMTTKCSFNASTDFMGKLR  56

Query  60    EKFKGEDSNFPAAAAVQPAADMPSNLCIIDTLQRLGVDRYFRSEIDTILEDTYRLW-QRK  118
             EK  G+    P    + P  D+ SNLC+IDTLQ LGVDRYF+SEI+T+LE TYRLW ++K
Sbjct  57    EKVGGKADKPPVV--IHPV-DISSNLCMIDTLQSLGVDRYFQSEINTLLEHTYRLWKEKK  113

Query  119   ERAIFSDTTIHAMAFRLLRVKGYEVSSEELAPYADQEHVDLQTIEVATVIELYRAAQERT  178
             +  IF D +  A+AFRLLR KGY+VSS++LAP+AD    D     VAT++ELYRA+Q R
Sbjct  114   KNIIFKDVSCCAIAFRLLREKGYQVSSDKLAPFADYRIRD-----VATILELYRASQARL  168

Query  179   GEDESSLKKLHAWTTTFLKQKLLTNSIPDKKLHKLVEYYLKNYHGILDRMGVRQNLDLYD  238
              EDE +L+KLH W++  LKQ LL  SIPD KLHK VEY+LKNYHGILDR+ VR++LDLY+
Sbjct  169   YEDEHTLEKLHDWSSNLLKQHLLNGSIPDHKLHKQVEYFLKNYHGILDRVAVRRSLDLYN  228

Query  239   ISYY-RTAKAANRFSNLCSEDFLAFARQDFNICQAQHQKELQQLQRWYADCKLDTLKYGR  297
             I+++ R    A+ F      EDFL ++ QDFNICQAQ Q+EL QLQRWYADC+LDTL YGR
Sbjct  229   INHHHRIPDVADGFPK---EDFLEYSMQDFNICQAQQQEELHQLQRWYADCRLDTLNYGR  285

Query  298   DVVRVANFLTSAIIGDPELSDVRIVFAQHIVLVTRIDDFFDHRGSREESYKILELIKEWK  357
             DVVR+ANFLTSAI G+PE SD R+ FA+HI+LVTRIDDFFDH GSREESYKIL+L++EWK
Sbjct  286   DVVRIANFLTSAIFGEPEFSDARLAFAKHIILVTRIDDFFDHGGSREESYKILDLVQEWK  345

Query  358   EKPAAEYGSEEVEILFTAVYNTVNELAERAHVEHGRSVKDFLIKLWVQILSIFKRELDTW  417
             EKPA EYGS+EVEILFTAVYNTVN+LAE+AH+E GR VK  LIKLWV+IL+ FK+ELD+W
Sbjct  346   EKPAEEYGSKEVEILFTAVYNTVNDLAEKAHIEQGRCVKPLLIKLWVEILTSFKKELDSW  405

Query  418   SDDTALTLDDYLSASWVSIGCRICILMSMQFIGIKLSDEMLLSEECIDLCRHVSMVDRLL  477
             +++TALTLD+YLS+SWVSIGCRICIL S+Q++GIKLS+EML S+EC DLCRHVS VDRLL
Sbjct  406   TEETALTLDEYLSSSWVSIGCRICILNSLQYLGIKLSEEMLSSQECTDLCRHVSSVDRLL  465

Query  478   NDVQTFEKERKENTGNSVTLLLAANKDDSSFTEEEAIRIAKEMAECNRRQLMQIVYKTGT  537
             NDVQTF+KER ENT NSV L LAA+K + + TEE+A+   KEMA+ +RR+LMQIVYK GT
Sbjct  466   NDVQTFKKERLENTINSVGLQLAAHKGERAMTEEDAMSKIKEMADYHRRKLMQIVYKEGT  525

Query  538   IFPRQCKDMFLKVCRIGCYLYASGDEFTSPQQMMEDMKSLVYEPLTIHPLVANNV  592
             +FPR+CKD+FL+VCRIG YLY+SGDEFTSPQQM EDMKSLVY+P+ IHPL A NV
Sbjct  526   VFPRECKDVFLRVCRIGYYLYSSGDEFTSPQQMKEDMKSLVYQPVKIHPLEAINV  580
```

RecName: Full=Cis-abienol synthase, chloroplastic; Short=NtABS; Flags: Precursor [Nicotiana tabacum]

Sequence ID: **G3CCC1.1** Length: 792 Number of Matches: 1

Range 1: 284 to 788

Score	Expect	Method	Identities	Positives	Gaps	Frame
371 bits(952)	6e-118()	Compositional matrix adjust.	190/508(37%)	309/508(60%)	10/508(1%)	

```
Query  83    SNLCIIDTLQRLGVDRYFRSEIDTILEDTYRLWQRKERAIFSDTTIHAMAFRLLRVKGYE  142
             SNL ++D LQ LGVDRYF++E+  +L++ YRLW  K   IFSD    AMAFRLLR+  YE
Sbjct  284   SNLFLVDALQNLGVDRYFKTEVKRVLDEIYRLWLEKNEEIFSDVAHCAMAFRLLRMNNYE  343

Query  143   VSSEELAPYADQEHV----DLQTIEVATVIELYRAAQERTGEDESS-LKKLHAWTTTFLK  197
             VSSEEL   + DQEH       ++EL+RA+Q     E +    L K+  WT  F++
Sbjct  344   VSSEELEGFVDQEHFFTTSSGKLMNHVAILELHRASQVAIHERKDHILDKISTWTRNFME  403

Query  198   QKLLTNSIPDKKLHKLVEYYLKNYHGILDRMGVRQNLDLYDISYYRTAKAANRFSNLCSE  257
             QKLL   IPD+   K +E+ ++ ++G  DR+  R+ ++ Y +  ++  KAA R S + +
Sbjct  404   QKLLDKHIPDRS-KKEMEFAMRKFYGTFDRVETRRYIESYKMDSFKILKAAYRSSGINNI  462

Query  258   DFLAFARQDFNICQAQHQKELQQLQRWYADCKLDTLKYGRDVVRVANFLTSAIIGDPELS  317
             D L F+  DFN+CQ +H++ELQQ++RW+ DCKL+ +   +  +  F+ +AI+ +PE +
Sbjct  463   DLLKFSEHDFNLCQTRHKEELQQMKRWFTDCKLEQVGLSQQYLYTSYFIIAAILFEPEYA  522

Query  318   DVRIVFAQHIVLVTRIDDFFDHRGSREESYKILELIKEWKEKPAAEYGSEEVEILFTAVY  377
             D R+ +A++ +++T +DDFFD    +EE   I+EL++ W+       + SE V I F A+Y
Sbjct  523   DARLAYAKYAIIITAVDDFFDCFICKEELQNIIELVERWEGYSTVGFRSERVRIFFLALY  582

Query  378   NTVNELAERAHVEHGRSVKDFLIKLWVQILSIFKRELDTWS-DDTALTLDDYLSASWVSI  436
               V E+A +A   + GR VKD LI LW+ +L       ELD W   T  ++++YLS + V+I
Sbjct  583   KMVEEIAAKAETKQGRCVKDHLINLWIDMLKCMLVELDLWKIKSTTPSIEEYLSVACVTI  642

Query  437   GCRICILMSMQFIGIKLSDEMLLSEECIDLCRHVSMVDRLLNDVQTFEKERKENTGNSVT  496
             G    +L S+  +G KLS +++ S E   LC   + V RL+ND+ ++++E+ E++ N V+
Sbjct  643   GVPCFVLTSLYLLGPKLSKDVIESSEVSALCNCTAAVARLINDIHSYKREQAESSTNMVS  702

Query  497   LLLAANKDDSSFTEEEAIRIAKEMAECNRRQLMQIVYKTG-TIFPRQCKDMFLKVCRIGC  555
             +L+  ++    + +EEEAIR  KEM E  RR+L+ +V +     P+ CKD+F
Sbjct  703   ILITQSQ--GTISEEEAIRQIKEMMESKRRELLGMVLQNKESQLPQVCKDLFWTTINAAY  760
```

```
Query  556   YLYASGDEFTSPQQMMEDMKSLVYEPLT  583
              ++  GD +  P++    +  ++Y+PL
Sbjct  761   SIHTHGDGYRFPEEFKNHINDVIYKPLN  788
```

RecName: Full=Santalene and bergamotene synthase, chloroplastic; AltName: Full=(+)-alpha-santalene synthase ((2Z,6Z)-farnesyl diphosphate cyclizing); AltName: Full=(+)-endo-beta-bergamotene synthase ((2Z,6Z)-farnesyl diphosphate cyclizing); AltName: Full=(-)-endo-alpha-bergamotene synthase ((2Z,6Z)-farnesyl diphosphate cyclizing); Flags: Precursor [Solanum habrochaites]

Sequence ID: **B8XA41.1** Length: 777 Number of Matches: 1

Range 1: 275 to 776

Score	Expect	Method	Identities	Positives	Gaps	Frame
351 bits(900)	2e-110()	Compositional matrix adjust.	189/508(37%)	310/508(61%)	13/508(2%)	

```
Query  83    SNLCIIDTLQRLGVDRYFRSEIDTILEDTYRLWQRKERAIFSDTTIHAMAFRLLRVKGYE  142
             S LC++DTLQ LGV R+F+SEI   L++ YRLWQ+K   IFS+ T  AMAFRLLR+  Y+
Sbjct  275   SLLCLVDTLQNLGVHRHFKSEIKKALDEIYRLWQQKNEQIFSNVTHCAMAFRLLRMSYYD  334

Query  143   VSSEELAPYADQEH---VDLQTIEVATVIELYRAAQERTG-EDESSLKKLHAWTTTFLKQ  198
             VSS+ELA + D+EH   +  +      ++EL++A+Q     E +  L K++ WT TF++Q
Sbjct  335   VSSDELAEFVDEEHFFAISGKYTSHVEILELHKASQLAIDHEKDDILDKINNWTRTFMEQ  394

Query  199   KLLTNSIPDKKLHKLVEYYLKNYHGILDRMGVRQNLDLYDISYYRTAKAANRFSNLCSED  258
             KLL N   D+   K VE  L+ ++ I D    R+ +  Y+ + ++  KAA R  N+ ++D
Sbjct  395   KLLNNGFIDRMSKKEVELALRKFYTISDLAENRRCIKSYEENNFKILKAAYRSPNIYNKD  454

Query  259   FLAFARQDFNICQAQHQKELQQLQRWYADCKLDTLKYGRDVVRVANFLTSAIIGDPELSD  318
                F+ ++F +CQAQHQ+ELQQ +RW+ D +LD L      +         ++ +PELSD
Sbjct  455   LFIFSIRNFELCQAQHQEELQQFKRWFEDYRLDQLGIAERYIHDTYLCAVIVVPEPELSD  514

Query  319   VRIVFAQHIVLVTRIDDFFDHRGSREESYKILELIKEWKEKPAAEYGSEEVEILFTAVYN  378
              R+++A++++L+T +DD FD   S +E   I+EL++ W +  +  Y SE+V++ F+ +Y
Sbjct  515   ARLLYAKYVLLLTIVDDQFDSFASTDECLNIIELVERWDDYASVGYKSEKVKVFFSTLYK  574

Query  379   TVNELAERAHVEHGRSVKDFLIKLWVQILSIFKRELDTW-SDDTALTLDDYLSASWVSIG  437
             ++ EL   A ++ GRSVK+ L+ LW++++ +   E   W S  T  ++++YL  + ++ G
Sbjct  575   SIEELVTIAEIKQGRSVKNHLLNLWLELVKLMLMERVEWFSGKTIPSIEEYLYVTSITFG  634

Query  438   CRICILMSMQFIGIKLSDEMLLSEECIDLCRHVSMVDRLLNDVQTFEKERKENTGNSVTL  497
              R+  L +  F+GIK+S+++L S+E   LC     V R+LND+Q  +KE+KE++   VTL
Sbjct  635   ARLIPLTTQYFLGIKISEDILESDEIYGLCNCTGRVLRILNDLQDSKKEQKEDSVTIVTL  694

Query  498   LLAANKDDSSFTEEEAIRIAKEMAECNRRQLMQIVY--KTGTIFPRQCKDMFLKVCRIGC  555
             L+       S +EEEAI   KE+ E NRR+L+++V   K G+  P+ CKD+F +
Sbjct  695   LM------KSMSEEEAIMKIKEILEMNRRELLKMVLVQKKGSQLPQICKDIFWRTSNWAD  748

Query  556   YLYASGDEFTSPQQMMEDMKSLVYEPLT  583
             ++Y   D +   ++M   +  + Y+PL
Sbjct  749   FIYLQTDGYRIAEEMKNHIDEVFYKPLN  776
```

RecName: Full=Beta-phellandrene synthase (neryl-diphosphate-cyclizing), chloroplastic; Flags: Precursor [Solanum lycopersicum]

Sequence ID: **C1K5M3.1** Length: 778 Number of Matches: 1

Range 1: 276 to 777

Score	Expect	Method	Identities	Positives	Gaps	Frame
346 bits(888)	1e-108()	Compositional matrix adjust.	188/508(37%)	310/508(61%)	13/508(2%)	

```
Query  83    SNLCIIDTLQRLGVDRYFRSEIDTILEDTYRLWQRKERAIFSDTTIHAMAFRLLRVKGYE  142
             S LC++DTLQ LGV R+F+SEI   L++ YRLWQ+K   IFS+ T  AMAFRLLR+  Y+
Sbjct  276   SLLCLVDTLQNLGVHRHFKSEIKKALDEIYRLWQQKNEQIFSNVTHCAMAFRLLRMSYYD  335

Query  143   VSSEELAPYADQEH---VDLQTIEVATVIELYRAAQERTG-EDESSLKKLHAWTTTFLKQ  198
             VSS+ELA + D+EH    + +      ++EL++A+Q     E +  L K++ WT  F++Q
Sbjct  336   VSSDELAEFVDEEHFFATNGKYKSHVEILELHKASQLAIDHEKDDILDKINNWTRAFMEQ  395

Query  199   KLLTNSIPDKKLHKLVEYYLKNYHGILDRMGVRQNLDLYDISYYRTAKAANRFSNLCSED  258
             KLL N   D+   K VE  L+ ++        R+ +  Y+ + ++  KAA R  N+ ++D
Sbjct  396   KLLNNGFIDRMSKKEVELALRKFYTTSHLAENRRYIKSYEENNFKILKAAYRSPNINNKD  455

Query  259   FLAFARQDFNICQAQHQKELQQLQRWYADCKLDTLKYGRDVVRVANFLTSAIIGDPELSD  318
              LAF+  DF +CQAQH++ELQQL+RW+ D +LD L      +  +      +I +PELSD
Sbjct  456   LLAFSIHDFELCQAQHREELQQLKRWFEDYRLDQLGLAERYIHASYLFGVTVIPEPELSD  515

Query  319   VRIVFAQHIVLVTRIDDFFDHRGSREESYKILELIKEWKEKPAAEYGSEEVEILFTAVYN  378
              R+++A++++L+T +DD F+   S++E + I+EL++ W +  +  Y SE+V++ F+  Y
Sbjct  516   ARLMYAKYVMLLTIVDDHFESFASKDECFNIIELVERWDDYASVGYKSEKVKVFFSVFYK  575

Query  379   TVNELAERAHVEHGRSVKDFLIKLWVQILSIFKRELDTW-SDDTALTLDDYLSASWVSIG  437
             ++ ELA  A ++ GRSVK+ LI LW++++ +   E   W S  T  ++++YL  + ++
Sbjct  576   SIEELATIAEIKQGRSVKNHLINLWLELMKLMLMERVEWCSGKTIPSIEEYLYVTSITFC  635

Query  438   CRICILMSMQFIGIKLSDEMLLSEECIDLCRHVSMVDRLLNDVQTFEKERKENTGNSVTL  497
              ++  L +  F+GIK+S ++L S+E   L      V R+LND+Q  ++E+KE + N VTL
Sbjct  636   AKLIPLSTQYFLGIKISKDLLESDEICGLWNCSGRVMRILNDLQDSKREQKEVSINLVTL  695

Query  498   LLAANKDDSSFTEEEAIRIAKEMAECNRRQLMQIVY--KTGTIFPRQCKDMFLKVCRIGC  555
             L+       S +EEEAI   KE+ E NRR+L+++V   K G+  P+ CKD+F +  +
Sbjct  696   LM------KSMSEEEAIMKIKEILEMNRRELLKMVLVQKKGSQLPQLCKDIFWRTSKWAH  749

Query  556   YLYASGDEFTSPQQMMEDMKSLVYEPLT  583
             + Y+  D +   ++M   +  + Y+PL
Sbjct  750   FTYSQTDGYRIAEEMKNHIDEVFYKPLN  777
```

RecName: Full=Ent-kaurene synthase-like 2; Short=OsKSL2 [Oryza sativa Japonica Group]

Sequence ID: **Q0JA81.2** Length: 937 Number of Matches: 1

Range 1: 198 to 698

Score	Expect	Method	Identities	Positives	Gaps	Frame
316 bits(810)	8e-96()	Compositional matrix adjust.	172/504(34%)	299/504(59%)	8/504(1%)	

```
Query  69    FPAAAAVQPAADMPSNLCIIDTLQRLGVDRYFRSEIDTILEDTYRLWQRKERAIFSDTTI  128
             F +A      A++ S L ++D L+++G+ R+F  EI +IL+ TY  W++++  I  D
Sbjct  198   FGSAVPAAYPANIQSQLYMVDVLEKMGISRHFVGEIKSILDMTYSCWKQRDEEIVLDMQT  257
```

```
Query  129   HAMAFRLLRVKGYEVSSEELAPYADQE--HVDLQTI--EVATVIELYRAAQERTGEDESS  184
               MAFR+LR+ GY+VSS+EL+ +++    H  LQ    +  +++EL++A++    E E
Sbjct  258   CGMAFRMLRMNGYDVSSDELSHFSEPSSFHNSLQGYLNDTRSLLELHKASKVSIAEKEVI  317

Query  185   LKKLHAWTTTFLKQKLLTNSIPDKKLHKLVEYYLK-NYHGILDRMGVRQNLDLYDISYYR  243
             L  + +WT   LK++LL++++    L + VEY L+  ++ ILDR+  ++N++ +DI+  +
Sbjct  318   LDNIGSWTGCLLKEQLLSSAMKRNPLSEEVEYALEFPFYTILDRLDHKRNIEHFDITSSQ  377

Query  244   TAKAANRFSNLCSEDFLAFARQDFNICQAQHQKELQQLQRWYADCKLDTLKYGRDVVRVA  303
              + A       +E+ +A   +DF+  Q   Q+ELQQL  W  + +LD L++ R  +
Sbjct  378   MLETA-YLPCHSNEEIMALGVRDFSSSQFIFQEELQQLNSWVKESRLDQLQFARQKLDYF  436

Query  304   NFLTSAIIGDPELSDVRIVFAQHIVLVTRIDDFFDHRGSREESYKILELIKEWKEKPAAE  363
              F  +A I  PELSDVRI++A++ VL T +DDFFD  GS+EE   ++ L+++W +    E
Sbjct  437   YFSAAATIFTPELSDVRILWAKNGVLTTVVDDFFDVGGSKEELENLVALVEKWDKNDKTE  496

Query  364   YGSEEVEILFTAVYNTVNELAERAHVEHGRSVKDFLIKLWVQILSIFKRELDTWSDDTAL  423
             Y SE+VEI+F+A+Y + N+L   A V  GR V   L+++W ++L     E++
Sbjct  497   YYSEQVEIVFSAIYTSTNQLGSMASVVQGRDVTKHLVEIWQELLRSMMTEVEWRQSRYVP  556

Query  424   TLDDYLSASWVSIGCRICILMSMQFIGIKLSDEMLLSEECIDLCRHVSMVDRLLNDVQTF  483
             T ++Y+  + V+       +L ++  +G K+ D ++ S+EC +L R +S   RLLNDVQ++
Sbjct  557   TAEEYMENAVVTFALGPVVLPALYLVGPKMPDSVIRSQECSELFRLMSKCGRLLNDVQSY  616

Query  484   EKERKENTGNSVTLLLAANKDDSSFTEEEAIRIAKEMAECNRRQLMQIVYKTGTIFPRQC  543
             E+E  +   NSV+LL  A     S + EEA++  +   E  RR+L+++V   G   PR C
Sbjct  617   EREGSQGKLNSVSLL--ALHSGGSVSMEEAVKQIQRPIEKCRRELLKLVVSRGGAVPRPC  674

Query  544   KDMFLKVCRIGCYLYASGDEFTSP  567
             +++F  +C++  + Y+ GD F+SP
Sbjct  675   RELFWSMCKVCHFFYSGGDGFSSP  698
```

RecName: Full=Ent-kaur-16-ene synthase, chloroplastic; AltName: Full=Ent-kaurene synthase; AltName: Full=Ent-kaurene synthase B; Short=KSB; Flags: Precursor [Cucurbita maxima]

Sequence ID: **Q39548.1** Length: 789 Number of Matches: 1

Range 1: 239 to 787

Score	Expect	Method	Identities	Positives	Gaps	Frame
309 bits(791)	3e-94()	Compositional matrix adjust.	186/559(33%)	315/559(56%)	18/559(3%)	

```
Query  38    TNKSSFAVKCNLTTTDLMGKIAEKFKGEDSNFPAAAAVQPAADMPSNLCIIDTLQRLGVD  97
             T  ++F  + +     D +  + +KF G        +    + P  D+ + L ++D+LQ+ G+
Sbjct  239   TTAAAFMHRNDDGCFDYLRSLLQKFDG------SVPTIYPL-DIYARLHMVDSLQKFGIA  291

Query  98    RYFRSEIDTILEDTYRLWQRKERAIFSDTTIHAMAFRLLRVKGYEVSSEELAPYADQ---  154
             R+F+ EI ++L++TYR W + E  IF D +  AMAFR+LRV+GY+VSS+L  +++
Sbjct  292   RHFKEEIRSVLDETYRCWMQGEENIFLDASTCAMAFRMLRVEGYDVSSDQLTQFSEDIFP  351

Query  155   EHVDLQTIEVATVIELYRAAQERTGEDESSLKKLHAWTTTFLKQKLLTNSIPDKKLHKLV  214
               +     +   +ELY+A+Q  T  DES L+ +++WT+ FLK  L ++S+   +   +V
Sbjct  352   NCLGGYLKDFGASLELYKASQIITHPDESVLENINSWTSRFLKHGLSSDSVWSDRTDSVV  411

Query  215   EYYLKN-----YHGILDRMGVRQNLDLYDISYYRTAKAANRFSNLCSEDFLAFARQDFNI  269
             +    N      Y+  L+R+  ++ ++ Y      R +K+      N   +DFL  A +DFN
Sbjct  412   KQEAVNALEFPYNATLERLISKRAMESYSGDIVRISKSPYACLNFGHQDFLELAVEDFNT  471

Query  270   CQAQHQKELQQLQRWYADCKLDTLKYGRDVVRVANFLTSAIIGDPELSDVRIVFAQHIVL  329
              Q  H KEL++LQRW   + KLD LK+  R  +    F  +A + DPEL D RI +AQ+ VL
Sbjct  472   LQRIHLKELEELQRWVVENKLDELKFFRLHLGYCYFAAAATLTDPELHDARIAWAQNGVL  531

Query  330   VTRIDDFFDHRGSREESYKILELIKEWKEKPAAEYGSEEVEILFTAVYNTVNELAERAHV  389
              T +DDF+D  GS EE   ++EL+++W        Y S++VEI+F A+++TV E+  RA V
Sbjct  532   TTVVDDFYDGGGSEEELDNLIELVEKWDPDGEVGYCSKDVEIVFLALHSTVCEIGRRALV  591

Query  390   EHGRSVKDFLIKLWVQILSIFKRELDTWSDDTALTLDDYLSASWVSIGCRICILMSMQFI  449
                GRSV   +I  W+ +L + ++E +  ++    ++ +Y+  + VS      IL   + F+
Sbjct  592   WQGRSVMRNVIDGWLALLKVMRKEAEWSTNKVVPSMGEYMEQAHVSFALGPIILPMLFFV  651

Query  450   GIKLSDEMLLSEECIDLCRHVSMVDRLLNDVQTFEKERKENTGNSVTLLLAANKDDSSFT  509
             G KLS+EM+ S E   L + +S   RL ND++++++E KE   N ++L +        + T
Sbjct  652   GPKLSEEMIGSCEYQKLYKLMSTAGRLKNDIRSYDRECKEGKLNILSLWMIDGG--GNVT  709

Query  510   EEEAIRIAKEMAECNRRQLMQIVYKTGTIFPRQCKDMFLKVCRIGCYLYASGDEFTSPQQ  569
             +EEAI   K   E   R+L+ +V +  T  PR CKD+F K+  I   Y   D +TS  +
Sbjct  710   KEEAIEAIKGDFERAIRELLGLVLQENTTIPRACKDLFWKLMSIVNLFYMEDDGYTS-NR  768

Query  570   MMEDMKSLVYEPLTIHPLV  588
             +M  +K++  +P+ +  L+
Sbjct  769   LMNTVKAMFEQPMDLDALL  787
```

RecName: Full=Ent-kaur-16-ene synthase, chloroplastic; AltName: Full=Ent-kaurene synthase; Short=AtKS; AltName: Full=Ent-kaurene synthase B; Short=KSB; AltName: Full=Protein GA REQUIRING 2; Flags: Precursor [Arabidopsis thaliana]

Sequence ID: **Q9SAK2.1** Length: 785 Number of Matches: 1

Range 1: 259 to 780

Score	Expect	Method	Identities	Positives	Gaps	Frame
305 bits(781)	8e-93()	Compositional matrix adjust.	186/528(35%)	299/528(56%)	17/528(3%)	

```
Query  69    FPAAAAVQPAADMPSNLCIIDTLQRLGVDRYFRSEIDTILEDTYRLWQRKERAIFSDTTI  128
             F AA        D   + L II TL+ LG+DR F++EI  +IL++TYR W R +  I  D
Sbjct  259   FEAAVPSVYPFDQYARLSIIVTLESLGIDRDFKTEIKSILDETYRYWLRGDEEICLDLAT  318

Query  129   HAMAFRLLRVKGYEVSSEELAPYADQEHVDLQTIE-----VATVIELYRAAQERTGEDES  183
              A+AFRLL   GY+VS + L P+A++       T+E       +V+EL++AAQ   +   ES
Sbjct  319   CALAFRLLLAHGYDVSYDPLKPFAEESGFS-DTLEGYVKNTFSVLELFKAAQ--SYPHES  375

Query  184   SLKKLHAWTTTFLKQKL---LTNSIPDKKLHKLVEYYL--KNYHGILDRMGVRQNLDLYD  238
             +LKK   WT  +L+ +L    +  S+ DK L K VE  L   +Y  +      R+ L+
Sbjct  376   ALKKQCCWTKQYLEMELSSWVKTSVRDKYLKKEVEDALAFPSYASLERSDHRRKILNGSA  435

Query  239   ISYYRTAKAANRFSNLCSEDFLAFARQDFNICQAQHQKELQQLQRWYADCKLDTLKYGRD  298
             +   R  K + R  N+C+ D L  A  DFN CQ+ H++E+++L RW  + +L  LK+ R
Sbjct  436   VENTRVTKTSYRLHNICTSDILKLAVDDFNFCQSIHREEMERLDRWIVENRLQELKFARQ  495

Query  299   VVRVANFLTSAIIGDPELSDVRIVFAQHIVLVTRIDDFFDHRGSREESYKILELIKEWKE  358
              +    F  +A +  PELSD RI +A+  VL T +DDFFD  GS+EE   ++ L+++W
Sbjct  496   KLAYCYFSGAATLFSPELSDARISWAKGGVLTTVVDDFFDVGGSKEELENLIHLVEKWDL  555
```

```
Query  359  KPAAEYGSEEVEILFTAVYNTVNELAERAHVEHGRSVKDFLIKLWVQILSIFKRELDTWS  418
                EY SE VEI+F+ + +T+ E  ++A    GR+V   ++K+W+ +L    RE +  S
Sbjct  556  NGVPEYSSEHVEIIFSVLRDTILETGDKAFTYQGRNVTHHIVKIWLDLLKSMLREAEWSS  615

Query  419  DDTALTLDDYLSASWVSIGCRICILMSMQFIGIKLSDEMLLSEECIDLCRHVSMVDRLLN  478
            D +  +L+DY+  +++S      +L +   IG  L ++ + S +   L + VS + RLLN
Sbjct  616  DKSTPSLEDYMENAYISFALGPIVLPATYLIGPPLPEKTVDSHQYNQLYKLVSTMGRLLN  675

Query  479  DVQTFEKERKENTGNSVTLLLAANKDDSSFTEEEAIRIAKEMAECNRRQLMQIVY-KTGT  537
            D+Q F++E  E   N+V+L +   +D+ S  +E  I   K +AE  R +L ++V  + G+
Sbjct  676  DIQGFKRESAEGKLNAVSLHMKHERDNRS--KEVIIESMKGLAERKREELHKLVLEEKGS  733

Query  538  IFPRQCKDMFLKVCRIGCYLYASGDEFTSPQQMMEDMKSLVYEPLTIH  585
            + PR+CK+ FLK+ ++    Y   D FTS   +M  +KS++YEP+++
Sbjct  734  VVPRECKEAFLKMSKVLNLFYRKDDGFTS-NDLMSLVKSVIYEPVSLQ  780
```

Taxonomy

Reports

Lineage

Organism	Blast Name	Score	Number of Hits	Description
Mesangiospermae	flowering plants		10	
.Pentapetalae	eudicots		9	
..lamiids	eudicots		7	
...Lamiaceae	eudicots		4	
....Salvia miltiorrhiza	eudicots	1233	1	**Salvia miltiorrhiza hits**
....Vitex agnus-castus	eudicots	826	2	**Vitex agnus-castus hits**
....Marrubium vulgare	eudicots	766	1	**Marrubium vulgare hits**
...Nicotiana tabacum	eudicots	371	1	**Nicotiana tabacum hits**
...Solanum habrochaites	eudicots	351	1	**Solanum habrochaites hits**
...Solanum lycopersicum	eudicots	346	1	**Solanum lycopersicum hits**
..Cucurbita maxima	eudicots	309	1	**Cucurbita maxima hits**
..Arabidopsis thaliana	eudicots	305	1	**Arabidopsis thaliana hits**
.Oryza sativa Japonica Group	monocots	316	1	**Oryza sativa Japonica Group hits**

Organism

Description	Score	E value	Accession
Salvia miltiorrhiza [eudicots]			
**RecName: Full=Miltiradiene synthase KSL1, chloroplastic; AltName: Full=Kaurene synthase-like 1; Short=SmKSL; Short=SmKSL1; Flags: Precursor [Salvia miltiorrhiza]**	1233	0.0	**C8XPS0**
Vitex agnus-castus [eudicots]			
**RecName: Full=Class I diterpene synthase 2, chloroplastic; Short=VacTPS2; AltName: Full=(13S)-vitexifolin A synthase; AltName: Full=9,13-epoxylabda-14-ene synthase; AltName: Full=Viteagnusin D synthase; Flags: Precursor [Vitex agnus-castus]**	826	0.0	**A0A2K9RFZ2**
**RecName: Full=Class I diterpene synthase TPS6, chloroplastic; AltName: Full=Labd-13(16),14-diene-9-ol synthase; AltName: Full=Syn-isopimara-7,15-diene synthase; AltName: Full=Terpene synthase 6; Short=VacTPS6 [Vitex agnus-castus]**	795	0.0	**A0A2K9RFZ9**
Marrubium vulgare (white horehound) [eudicots]			
**RecName: Full=9,13-epoxylabda-14-ene synthase, chloroplastic; AltName: Full=Manoyl oxide synthase; AltName: Full=Miltiradiene synthase; Flags: Precursor [Marrubium vulgare]**	766	0.0	**A0A075FBG7**
Nicotiana tabacum (common tobacco) [eudicots]			
**RecName: Full=Cis-abienol synthase, chloroplastic; Short=NtABS; Flags: Precursor [Nicotiana tabacum]**	371	6e-118	**G3CCC1**
Solanum habrochaites [eudicots]			
**RecName: Full=Santalene and bergamotene synthase, chloroplastic; AltName: Full=(+)-alpha-santalene synthase ((2Z,6Z)-farnesyl diphosphate cyclizing); AltName: Full=(+)-endo-beta-bergamotene synthase ((2Z,6Z)-farnesyl diphosphate cyclizing); AltName: Full=(-)-endo-alpha-bergamotene synthase ((2Z,6Z)-farnesyl diphosphate cyclizing); Flags: Precursor [Solanum habrochaites]**	351	2e-110	**B8XA41**
Solanum lycopersicum (tomato) [eudicots]			
**RecName: Full=Beta-phellandrene synthase (neryl-diphosphate-cyclizing), chloroplastic; Flags: Precursor [Solanum lycopersicum]**	346	1e-108	**C1K5M3**
Oryza sativa Japonica Group (Japanese rice) [monocots]			
**RecName: Full=Ent-kaurene synthase-like 2; Short=OsKSL2 [Oryza sativa Japonica Group]**	316	8e-96	**Q0JA81**
Cucurbita maxima (winter squash) [eudicots]			
**RecName: Full=Ent-kaur-16-ene synthase, chloroplastic; AltName: Full=Ent-kaurene synthase; AltName: Full=Ent-kaurene synthase B; Short=KSB; Flags: Precursor [Cucurbita maxima]**	309	3e-94	**Q39548**
Arabidopsis thaliana (thale cress) [eudicots]			
**RecName: Full=Ent-kaur-16-ene synthase, chloroplastic; AltName: Full=Ent-kaurene synthase; Short=AtKS; AltName: Full=Ent-kaurene synthase B; Short=KSB; AltName: Full=Protein GA REQUIRING 2; Flags: Precursor [Arabidopsis thaliana]**	305	8e-93	**Q9SAK2**

Taxonomy
