# Supplementary material for: Genome-wide identification and functional characterization of natural antisense transcripts in Salvia miltiorrhiza
Source: Sci Rep. 2021 Feb 26;11:4769. doi: 10.1038/s41598-021-83520-6 (PMC7910453; doi:10.1038/s41598-021-83520-6)
Supplement: Supplementary file 4 — Supplementary Information. [file 41598_2021_83520_MOESM4_ESM.pdf]

COVID-19 is an emerging, rapidly evolving situation.
Get the latest public health information from CDC: https://www.coronavirus.gov .
Get the latest research from NIH: https://www.nih.gov/coronavirus.
Find NCBI SARS-CoV-2 literature, sequence, and clinical content: https://www.ncbi.nlm.nih.gov/sars-cov-2/.

**Job Title**	ST0002 ...
**RID**	MYHK7N21016 Search expires on 09-03 15:14 pm
**Program**	BLASTP
**Database**	swissprot
**Query ID**	lcl|Query_94544
**Description**	unnamed protein product ...
**Molecule type**	amino acid
**Query Length**	468

Descriptions

Description	Max Score	Total Score	Query Cover	E value	Per. Ident	Accession
RecName: Full=UDP-glucose flavonoid 3-O-glucosyltransferase 6; AltName: Full=Flavonol 3-O-glucosyltransferase 6; Short=FaGT6 [Fragaria x ananassa]	442	442	97%	2e-151	48.54%	Q2V6K0.1
RecName: Full=UDP-glycosyltransferase 71A15; AltName: Full=UDP-glucose:chalcone 2'-O-glucosyltransferase; AltName: Full=UDP-glucose:flavonol 2'-O-glucosyltransferase [Malus domestica]	436	436	97%	5e-149	47.99%	D3THI6.1
RecName: Full=Putative UDP-glucose flavonoid 3-O-glucosyltransferase 3; Short=FaGT3; AltName: Full=Flavonol 3-O-glucosyltransferase 3 [Fragaria x ananassa]	431	431	98%	7e-147	46.33%	Q66PF3.1
RecName: Full=UDP-glycosyltransferase 71A16; AltName: Full=UDP-glucose:chalcone 2'-O-glucosyltransferase; AltName: Full=UDP-glucose:flavonol 2'-O-glucosyltransferase [Pyrus communis]	426	426	97%	5e-145	47.12%	D3UAG1.1
RecName: Full=UDP-glycosyltransferase 71E1 [Stevia rebaudiana]	424	424	97%	2e-144	49.26%	Q6VAB2.1
RecName: Full=UDP-glycosyltransferase 101; Short=UGTPg101 [Panax ginseng]	402	402	97%	7e-136	46.44%	A0A0K0PVM5.1
RecName: Full=UDP-glycosyltransferase 1; Short=UGTPg1 [Panax ginseng]	402	402	97%	7e-136	46.65%	A0A068J840.1
RecName: Full=UDP-glycosyltransferase 71A27; Short=PgUGT71A27 [Panax ginseng]	390	390	97%	5e-131	45.59%	A0A0A7HB61.1
RecName: Full=UDP-glycosyltransferase 100; Short=UGTPg100 [Panax ginseng]	385	385	97%	3e-129	46.22%	A0A0K0PVW1.1
RecName: Full=UDP-glucosyltransferase 102; Short=UGTPg102 [Panax ginseng]	384	384	97%	7e-129	44.77%	A0A0K0PVL0.1

Graphic Summary

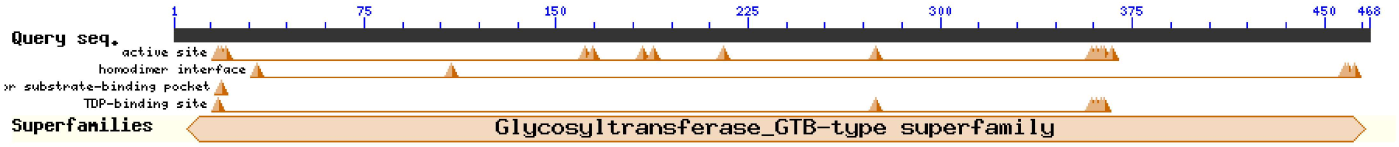

Distribution of the top 10 Blast Hits on 10 subject sequences

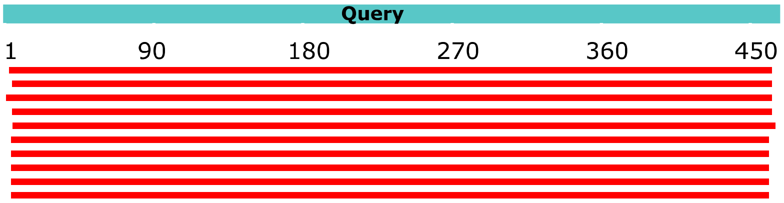

Alignments

Alignment view Pairwise ▼ ☐ CDS feature Restore defaults

RecName: Full=UDP-glucose flavonoid 3-O-glucosyltransferase 6; AltName: Full=Flavonol 3-O-glucosyltransferase 6; Short=FaGT6 [Fragaria x ananassa]

Sequence ID: **Q2V6K0.1** Length: 479 Number of Matches: 1

Range 1: 2 to 473

Score	Expect	Method	Identities	Positives	Gaps	Frame
442 bits(1137)	2e-151()	Compositional matrix adjust.	232/478(49%)	315/478(65%)	27/478(5%)	

```
Query  5    EKAS-LVFIPFPVVSHLVTAVKTAELLASHDSRLSITVLVMSMP-----TDTKISSY---  55
            +KAS L+FIP P + H+V+ V+ A+LL   D  L IT+L+M  P     +D  I S
Sbjct  2    KKASELIFIPIPGIGHIVSTVEIAKLLLCRDDNLFITILIMKFPFTADGSDVYIKSLAVD  61

Query  56   --IKNPRINFVQLEQDVSNGAEAIMKPPKSMMHFAGRHRDSARAVVSEM----KRSCRVA  109
              +K  RI FV L Q+   G  A          F   H+   +  V+ +      + R+A
Sbjct  62   PSLKTQRIRFVNLPQEHFQGTGAT-----GFFTFIDSHKSHVKDAVTRLMETKSETTRIA  116

Query  110  GIFVDIMCVDMIDVAKELKISSYIFFASGAAVLGLTFDLQSLRDDGGRNLAEFEGSDEVV  169
            G  +D+ C  MID+A E  + SY+F+ SGAA LGL F LQ+LRD+  ++  EF+ SD  +
Sbjct  117  GFVIDMFCTGMIDLANEFGLPSYVFYTSGAADLGLMFHLQALRDEENKDCTEFKDSDAEL  176

Query  170  SISSYVNPVPA-RVWPESVFDGESG--FLELSRKAREADGIVINTFLELESYAIGSTYAN  226
             +SS+VNP+PA RV P  VF+ E G  FL  +++ RE  GI++NTFLELE +AI S  ++
Sbjct  177  VVSSFVNPLPAARVLPSVVFEKEGGNFFLNFAKRYRETKGILVNTFLELEPHAIQSLSSD  236

Query  227  ERIPRFYPIGPIIG---EGKDENDESRQRRGEIMRWLDGQPDSSVVFLCFGSMGAFGEEQ  283
             +I   YP+GPI+    EG   + E  +++ +I+ WLD QP SSVVFLCFGSMG FGE+Q
Sbjct  237  GKILPVYPVGPILNVKSEGNQVSSEKSKQKSDILEWLDDQPPSSVVFLCFGSMGCFGEDQ  296

Query  284  VVDIAEALERSGKRFLWSLRKPIFEGGFAYPTEYENPGEVLPVGFLERTAGVGKVIGWAP  343
            V +IA ALE+ G RFLWSLR+P  E    +P++Y +   VLP GFL+RT  +GKVIGWAP
Sbjct  297  VKEIAHALEQGGIRFLWSLRQPSKEK-IGFPSDYTDYKAVLPEGFLDRTTDLGKVIGWAP  355

Query  344  QVAVLSHPSVGGFVSHCGWNSTLESVCCGVPMAAWPLGAEQQTNAFQLVKDIGIAVEIKM  403
            Q+A+L+HP+VGGFVSHCGWNSTLES+  GVP+A WP  AEQQ NAF+LVK++ +AVEI M
Sbjct  356  QLAILAHPAVGGFVSHCGWNSTLESIWYGVPIATWPFYAEQQVNAFELVKELKLAVEIDM  415

Query  404  DYRKNSGEIVPKSIIEKAIKQLMDPTNEIRVRVKELKEKSTRALMEGGSSYNHLGLLI  461
             YRK+SG IV +  IEK IK++M+  +E+R RVKE+ + S +AL E GSSY+ LG  +
Sbjct  416  GYRKDSGVIVSRENIEKGIKEVMEQESELRKRVKEMSQMSRKALEEDGSSYSSLGRFL  473
```

RecName: Full=UDP-glycosyltransferase 71A15; AltName: Full=UDP-glucose:chalcone 2'-O-glucosyltransferase; AltName: Full=UDP-glucose:flavonol 2'-O-glucosyltransferase [Malus domestica]

Sequence ID: **D3THI6.1** Length: 471 Number of Matches: 1

Range 1: 5 to 468

Score	Expect	Method	Identities	Positives	Gaps	Frame
436 bits(1120)	5e-149()	Compositional matrix adjust.	227/473(48%)	308/473(65%)	27/473(5%)	

```
Query  7    ASLVFIPFPVVSHLVTAVKTAELLASHDSRLSITVLVMSMP-------TDTKISSYIKNP  59
            A LVF+P P + H+V+ V+ A+ LA+ D +L ITVLVM +P       TD+ IS
Sbjct  5    AQLVFVPAPGIGHIVSTVEMAKQLAARDDQLFITVLVMKLPYAQPFTNTDSSISH-----  59

Query  60   RINFVQLEQDVSNGAEAIMKPPKSMMHFAGRHRDSARAVV---------SEMKRSCRVAG  110
            RINFV L +   +  + +  P      F   H+   R  V         SE     R+AG
Sbjct  60   RINFVNLPEAQPDKQDIVPNPGSFFRMFVENHKSHVRDAVINVLPESDQSESTSKPRLAG  119

Query  111  IFVDIMCVDMIDVAKELKISSYIFFASGAAVLGLTFDLQSLRDDGGRNLAEFEGSDEVVS  170
              +D+    +IDVA E K+ SY+FF S A+ L L    QSLRD+GG ++ E   S   ++
Sbjct  120  FVLDMFSASLIDVANEFKVPSYLFFTSNASALALMSHFQSLRDEGGIDITELTSSTAELA  179

Query  171  ISSYVNPVPARVWPESVFDGESGFLELSR--KAREADGIVINTFLELESYAIGSTYANER  228
            + S++NP PA V P S+ D ES    L+   K ++  GI++NTF+ELES+A+    + ++
Sbjct  180  VPSFINPYPAAVLPGSLLDMESTKSTLNHVSKYKQTKGILVNTFMELESHALHYLDSGDK  239

Query  229  IPRFYPIGPIIGEGKDENDESRQRRGEIMRWLDGQPDSSVVFLCFGSMGAFGEEQVVDIA  288
            IP  YP+GP++     + D++     +I+RWLD QP  SVVFLCFGSMG+FGE QV +IA
Sbjct  240  IPPVYPVGPLLNLKSSDEDKA----SDILRWLDDQPPFSVVFLCFGSMGSFGEAQVKEIA  295

Query  289  EALERSGKRFLWSLRKPIFEGGFAYPTEYENPGEVLPVGFLERTAGVGKVIGWAPQVAVL  348
             ALE SG RFLWSLR+P  +G  A P++YE+   VLP GFL+RTA VGKVIGWAPQ A+L
Sbjct  296  CALEHSGHRFLWSLRRPPPQGKRAMPSDYEDLKTVLPEGFLDRTATVGKVIGWAPQAAIL  355

Query  349  SHPSVGGFVSHCGWNSTLESVCCGVPMAAWPLGAEQQTNAFQLVKDIGIAVEIKMDYRKN  408
             HP+ GGFVSHCGWNSTLES+  GVP+AAWPL AEQ  NAFQLV ++G+AVEIKMDYR++
Sbjct  356  GHPATGGFVSHCGWNSTLESLWNGVPIAAWPLYAEQNLNAFQLVVELGLAVEIKMDYRRD  415

Query  409  SGEIVPKSIIEKAIKQLMDPTNEIRVRVKELKEKSTRALMEGGSSYNHLGLLI  461
            S  +V    IE+ I+++M+  +++R RVKE+ EKS +AL++GGSSY+ LG  I
Sbjct  416  SDVVVSAEDIERGIRRVMELDSDVRKRVKEMSEKSKKALVDGGSSYSSLGRFI  468
```

RecName: Full=Putative UDP-glucose flavonoid 3-O-glucosyltransferase 3; Short=FaGT3; AltName: Full=Flavonol 3-O-glucosyltransferase 3 [Fragaria x ananassa]

Sequence ID: **Q66PF3.1** Length: 478 Number of Matches: 1

Range 1: 1 to 475

Score	Expect	Method	Identities	Positives	Gaps	Frame
431 bits(1107)	7e-147()	Compositional matrix adjust.	221/477(46%)	315/477(66%)	20/477(4%)	

```
Query  3    VDEKASLVFIPFPVVSHLVTAVKTAELLASHDSRLSITVLVMSMPTDTK-ISSYIKN---  58
            +++ A LV IP P + HLV+ ++ A+LL S D +L ITVL+M  P  +K   +Y+++
Sbjct  1    MEKPAELVLIPSPGIGHLVSTLEIAKLLVSRDDKLFITVLIMHFPAVSKGTDAYVQSLAD  60

Query  59   ------PRINFVQLEQDVSNGAEAIMKPPKSMMHFAGRHRDSARAVVSEMK--RSCRVAG  110
```

```
                   RINF+ L     +  E  ++   S++ F    +   +  V+ ++  ++ R+AG
Sbjct  61    SSSPISQRINFINLPHTNMDHTEGSVR--NSLVGFVESQQPHVKDAVANLRDSKTTRLAG  118

Query  111   IFVDIMCVDMIDVAKELKISSYIFFASGAAVLGLTFDLQSLRDDGGRNLAEFEGSDEVVS  170
               VD+ C  MI+VA +L + SY+FF SGAA LGL F LQ LRD   ++  EF+ SD   +
Sbjct  119   FVVDMFCTTMINVANQLGVPSYVFFTSGAATLGLLFHLQELRDQYNKDCTEFKDSDAELI  178

Query  171   ISSYVNPVPARVWPESVF--DGESGFLELSRKAREADGIVINTFLELESYAIGSTYANER  228
             I S+ NP+PA+V P  +   D    FL + ++ RE  GI++NTF +LES+A+ +  ++
Sbjct  179   IPSFFNPLPAKVLPGRMLVKDSAEPFLNVIKRFRETKGILVNTFTDLESHALHALSSDAE  238

Query  229   IPRFYPIGPIIGEGKDE---NDESRQRRGEIMRWLDGQPDSSVVFLCFGSMGAFGEEQVV  285
             IP  YP+GP++    +E   + +  +++ +I++WLD QP  SVVFLCFGSMG+F E QV
Sbjct  239   IPPVYPVGPLLNLNSNESRVDSDEVKKKNDILKWLDDQPPLSVVFLCFGSMGSFDESQVR  298

Query  286   DIAEALERSGKRFLWSLRKPIFEGGFAYPTEYENPGEVLPVGFLERTAGVGKVIGWAPQV  345
             +IA ALE +G RFLWSLR+    G  A+P++Y++   VLP GFL+RT G+GKVIGWAPQV
Sbjct  299   EIANALEHAGHRFLWSLRRSPPTGKVAFPSDYDDHTGVLPEGFLDRTGGIGKVIGWAPQV  358

Query  346   AVLSHPSVGGFVSHCGWNSTLESVCCGVPMAAWPLGAEQQTNAFQLVKDIGIAVEIKMDY  405
             AVL+HPSVGGFVSHCGWNSTLES+  GVP+A WPL AEQQ NAFQ VK++ +AVEI M Y
Sbjct  359   AVLAHPSVGGFVSHCGWNSTLESLWHGVPVATWPLYAEQQLNAFQPVKELELAVEIDMSY  418

Query  406   RKNSGEIVPKSIIEKAIKQLMD-PTNEIRVRVKELKEKSTRALMEGGSSYNHLGLLI  461
             R  S  +V    IE+ I+++M+  +++IR RVKE+ EK  +ALM+GGSSY  LG  I
Sbjct  419   RSKSPVLVSAKEIERGIREVMELDSSDIRKRVKEMSEKGKKALMDGGSSYTSLGHFI  475
```

RecName: Full=UDP-glycosyltransferase 71A16; AltName: Full=UDP-glucose:chalcone 2'-O-glucosyltransferase; AltName: Full=UDP-glucose:flavonol 2'-O-glucosyltransferase [Pyrus communis]

Sequence ID: **D3UAG1.1** Length: 471 Number of Matches: 1

Range 1: 5 to 468

Score	Expect	Method	Identities	Positives	Gaps	Frame
426 bits(1094)	5e-145()	Compositional matrix adjust.	221/469(47%)	300/469(63%)	19/469(4%)	

```
Query  7     ASLVFIPFPVVSHLVTAVKTAELLASHDSRLSITVLVMSMPTD---TKISSYIKNPRINF  63
             A LVF+P P + H+V+ V+ A+ L + D +L ITVLVM +P D   T   S I + RINF
Sbjct  5     AQLVFVPAPGIGHIVSTVEMAKQLVARDDQLFITVLVMKLPYDQPFTNTDSSISH-RINF  63

Query  64    VQLEQDVSNGAEAIMKPPKSMMHFAGRHRDSARAVV---------SEMKRSCRVAGIFVD  114
             V L +   +  + +  P      F   H+   R  V         SE     R+AG  +D
Sbjct  64    VNLPEAQLDKQDTVPNPGSFFRMFVENHKTHVRDAVINLLPESDQSESTSKPRLAGFVLD  123

Query  115   IMCVDMIDVAKELKISSYIFFASGAAVLGLTFDLQSLRDDGGRNLAEFEGSDEVVSISSY  174
             +    +IDVA E ++ SY+FF S ++ L L    QSLRD+GG ++ E   S   +++ S+
Sbjct  124   MFSASLIDVANEFEVPSYVFFTSNSSTLALLSHFQSLRDEGGIDITELTSSTAELAVPSF  183

Query  175   VNPVPARVWPESVFDGESGFLELSRKAR--EADGIVINTFLELESYAIGSTYANERIPRF  232
             +NP P  V P S  D ES    L+   R  +  GI++NTFLELES+A+    +  +IP
Sbjct  184   INPYPVAVLPGSFLDKESTKSTLNNVGRYKQTKGILVNTFLELESHALHYLDSGVKIPPV  243

Query  233   YPIGPIIGEGKDENDESRQRRGEIMRWLDGQPDSSVVFLCFGSMGAFGEEQVVDIAEALE  292
             YP+GP++       D+      +I+RWLD QP  SVVFLCFGSMG+FG+ QV +IA  LE
Sbjct  244   YPVGPLLNLKSSHEDKG----SDILRWLDDQPPLSVVFLCFGSMGSFGDAQVKEIACTLE  299

Query  293   RSGKRFLWSLRKPIFEGGFAYPTEYENPGEVLPVGFLERTAGVGKVIGWAPQVAVLSHPS  352
              SG RFLWSLR+P  +G  A P++Y +   VLP GFL+RTA VG+VIGWAPQ A+L HP+
Sbjct  300   HSGHRFLWSLRQPPSKGKRALPSDYADLKTVLPEGFLDRTATVGRVIGWAPQAAILGHPA  359

Query  353   VGGFVSHCGWNSTLESVCCGVPMAAWPLGAEQQTNAFQLVKDIGIAVEIKMDYRKNSGEI  412
             +GGFVSHCGWNSTLES+  GVP+AAWP+ AEQ  NAFQLV ++G+AVEIKMDYRK+S  +
Sbjct  360   IGGFVSHCGWNSTLESIWNGVPIAAWPMYAEQNMNAFQLVVELGLAVEIKMDYRKDSDVV  419

Query  413   VPKSIIEKAIKQLMDPTNEIRVRVKELKEKSTRALMEGGSSYNHLGLLI  461
             V    IE+ I+Q+M+  +++R RVKE+ EKS +AL++GGSSY+ LG  I
Sbjct  420   VSAEDIERGIRQVMELDSDVRKRVKEMSEKSKKALVDGGSSYSSLGRFI  468
```

RecName: Full=UDP-glycosyltransferase 71E1 [Stevia rebaudiana]

Sequence ID: **Q6VAB2.1** Length: 474 Number of Matches: 1

Range 1: 4 to 468

Score	Expect	Method	Identities	Positives	Gaps	Frame
424 bits(1090)	2e-144()	Compositional matrix adjust.	232/471(49%)	307/471(65%)	20/471(4%)	

```
Query  7     ASLVFIPFPVVSHLVTAVKTAELLASHDSRLSITVLVMSMPTDTKISSYIKN--PRINFV  64
             + LVFIP P   HL   V+ A+LL   D RLS+T++VM++    K ++  +   P + FV
Sbjct  4     SELVFIPSPGAGHLPPTVELAKLLLHRDQRLSVTIIVMNLWLGPKHNTEARPCVPSLRFV  63

Query  65    QLEQDVSNGAEAIMKPPKSMMHFAGRHRDSARAVVSEM--KRSCRVAGIFVDIMCVDMID  122
              +  D S    A++ P   +  F   H+   R +V  +    S R+AG  +D+ C+ M D
Sbjct  64    DIPCDES--TMALISPNTFISAFVEHHKPRVRDIVRGIIESDSVRLAGFVLDMFCMPMSD  121

Query  123   VAKELKISSYIFFASGAAVLGLTFDLQSLRDDGGRNLAEFEGSDEVVSISSYVNPVPARV  182
             VA E  + SY +F SGAA LGL F LQ  RD  G +  E + SD  +S+ SYVNPVPA+V
Sbjct  122   VANEFGVPSYNYFTSGAATLGLMFHLQWKRDHEGYDATELKNSDTELSVPSYVNPVPAKV  181

Query  183   WPESVFDGESG---FLELSRKAREADGIVINTFLELESYAIGSTYANER-IPRFYPIGPI  238
              PE V D E G   FL+L+ + RE+ GI++N+   +E +A+    +N   IP  +P+GPI
Sbjct  182   LPEVVLDKEGGSKMFLDLAERIRESKGIIVNSCQAIERHALEYLSSNNNGIPPVFPVGPI  241

Query  239   IGEGKDENDESRQRRGEIMRWLDGQPDSSVVFLCFGSMGAFGEEQVVDIAEALERSGKRF  298
             +     EN +   +  EIMRWL+ QP+SSVVFLCFGSMG+F E+QV +IA A+ERSG RF
Sbjct  242   LNL---ENKKDDAKTDEIMRWLNEQPESSVVFLCFGSMGSFNEKQVKEIAVAIERSGHRF  298

Query  299   LWSLRKPIFEGGFAYPTEYENPGEVLPVGFLERTAGVGKVIGWAPQVAVLSHPSVGGFVS  358
             LWSLR+P  +    +P EYEN  EVLP GFL+RT+ +GKVIGWAPQ+AVLSHPSVGGFVS
Sbjct  299   LWSLRRPTPKEKIEFPKEYENLEEVLPEGFLKRTSSIGKVIGWAPQMAVLSHPSVGGFVS  358
```

```
Query  359   HCGWNSTLESVCCGVPMAAWPLGAEQQTNAFQLVKDIGIAVEIKMDYRKNS------GEI  412
             HCGWNSTLES+ CGVPMAAWPL AEQ  NAF LV ++G+A EI+MDYR ++      G
Sbjct  359   HCGWNSTLESMWCGVPMAAWPLYAEQTLNAFLLVVELGLAAEIRMDYRTDTKAGYDGGME  418

Query  413   VPKSIIEKAIKQLMDPTNEIRVRVKELKEKSTRALMEGGSSYNHLGLLIHN  463
             V    IE  I++LM    EIR +VK++KEKS  A++EGGSSY  +G  I +
Sbjct  419   VTVEEIEDGIRKLMS-DGEIRNKVKDVKEKSRAAVVEGGSSYASIGKFIEH  468
```

RecName: Full=UDP-glycosyltransferase 101; Short=UGTPg101 [Panax ginseng]

Sequence ID: **A0A0K0PVM5.1** Length: 475 Number of Matches: 1

Range 1: 2 to 470

Score	Expect	Method	Identities	Positives	Gaps	Frame
402 bits(1034)	7e-136()	Compositional matrix adjust.	222/478(46%)	301/478(62%)	32/478(6%)	

```
Query  6     KASLVFIPFPVVSHLVTAVKTAELLASHDSRLSITVLVMSMPTDTKISSYIKN------P  59
             K+ L+F+P P + HLV  V+ A+L  S    LS+TVL+     DT + +Y K+      P
Sbjct  2     KSELIFLPAPAIGHLVGMVEMAKLFISRHENLSVTVLIAKFYMDTGVDNYNKSLLTNPTP  61

Query  60    RINFVQL-EQDVSNGAEAIMKPPKSMM-HFAGRHRDSARAVVSEMKRS--CRVAGIFVDI  115
             R+  V L E D  N    ++KP  ++          +   R ++S M +S   RV G+  D+
Sbjct  62    RLTIVNLPETDPQN---YMLKPRHAIFPSVIETQKTHVRDIISGMTQSESTRVVGLLADL  118

Query  116   MCVDMIDVAKELKISSYIFFASGAAVLGLTFDLQSLRDDGGRNLAEFEGSDEVVSISSYV  175
             + ++++D+A E  + +Y++  +GA  LGL F LQ+L +D  +++ EF  SD  + + S+
Sbjct  119   LFINIMDIANEFNVPTYVYSPAGAGHLGLAFHLQTL-NDKKQDVTEFRNSDTELLVPSFA  177

Query  176   NPVPARVWPESVFDGESGF---LELSRKAREADGIVINTFLELESYAIGSTYANERIPRF  232
             NPVPA V P    D E G+     L R+ RE+  I+INTF ELE YAI S   +  IP
Sbjct  178   NPVPAEVLPSMYVDKEGGYDYLFSLFRRCRESKAIIINTFEELEPYAINSLRMDSMIPPI  237

Query  233   YPIGPII---GEGKDENDESRQRRGEIMRWLDGQPDSSVVFLCFGSMGAFGEEQVVDIAE  289
             YP+GPI+   G+G++ ++ +       I+ WLD QP SSVVFLCFGS G F E QV +IA
Sbjct  238   YPVGPILNLNGDGQNSDEAAV-----ILGWLDDQPPSSVVFLCFGSYGTFQENQVKEIAM  292

Query  290   ALERSGKRFLWSLRKPIFEGGFAYPTEYENPGEVLPVGFLERTAGVGKVIGWAPQVAVLS  349
              LERSG RFLWSLR  I +G      +Y N  E+LPVGFL+RT+ VGKVIGWAPQVAVL
Sbjct  293   GLERSGHRFLWSLRPSIPKGETKLQLKYSNLEEILPVGFLDRTSCVGKVIGWAPQVAVLG  352

Query  350   HPSVGGFVSHCGWNSTLESVCCGVPMAAWPLGAEQQTNAFQLVKDIGIAVEIKMDYRK--  407
             H +VGGF+SHCGWNSTLESV CGVP+A WP+  EQQ NAF++VK++GIAVEI++DY+
Sbjct  353   HEAVGGFLSHCGWNSTLESVWCGVPVATWPMYGEQQLNAFEMVKELGIAVEIEVDYKNEY  412

Query  408   ---NSGEIVPKSIIEKAIKQLM--DPTNEIRVRVKELKEKSTRALMEGGSSYNHLGLL  460
                N+  IV    IE  IK+LM  +  +EIR +VKE+KEKS  A+ E GSSYN L  L
Sbjct  413   FNMNNDFIVRAEEIETKIKKLMMDEKNSEIRKKVKEMKEKSRLAMSENGSSYNSLAKL  470
```

RecName: Full=UDP-glycosyltransferase 1; Short=UGTPg1 [Panax ginseng]

Sequence ID: **A0A068J840.1** Length: 475 Number of Matches: 1

Range 1: 2 to 470

Score	Expect	Method	Identities	Positives	Gaps	Frame
402 bits(1034)	7e-136()	Compositional matrix adjust.	223/478(47%)	302/478(63%)	32/478(6%)	

```
Query  6     KASLVFIPFPVVSHLVTAVKTAELLASHDSRLSITVLVMSMPTDTKISSYIKN------P  59
             K+ L+F+P P + HLV  V+ A+L  S    LS+TVL+     DT + +Y K+      P
Sbjct  2     KSELIFLPAPAIGHLVGMVEMAKLFISRHENLSVTVLIAKFYMDTGVDNYNKSLLTNPTP  61

Query  60    RINFVQL-EQDVSNGAEAIMKPPKSMM-HFAGRHRDSARAVVSEMKRS--CRVAGIFVDI  115
             R+  V L E D  N    ++KP  ++          +   R ++S M +S   +V G+  D+
Sbjct  62    RLTIVNLPETDPQN---YMLKPRHAIFPSVIETQKTHVRDIISGMTQSESTQVVGLLADL  118

Query  116   MCVDMIDVAKELKISSYIFFASGAAVLGLTFDLQSLRDDGGRNLAEFEGSDEVVSISSYV  175
             + ++++D+A E  + +Y++  +GA  LGL F LQ+L +D  +++ EF  SD  + + S+
Sbjct  119   LFINIMDIANEFNVPTYVYSPAGAGHLGLAFHLQTL-NDKKQDVTEFRNSDTELLVPSFA  177

Query  176   NPVPARVWPESVFDGESGF---LELSRKAREADGIVINTFLELESYAIGSTYANERIPRF  232
             NPVPA V P    D E G+     L R+ RE+  I+INTF ELE YAI S   +  IP
Sbjct  178   NPVPAEVLPSMYVDKEGGYDYLFSLFRRCRESKAIIINTFEELEPYAINSLRMDSMIPPI  237

Query  233   YPIGPII---GEGKDENDESRQRRGEIMRWLDGQPDSSVVFLCFGSMGAFGEEQVVDIAE  289
             YP+GPI+   G+G++ ++ +       I+ WLD QP SSVVFLCFGS G+F E QV +IA
Sbjct  238   YPVGPILNLNGDGQNSDEAAV-----ILGWLDDQPPSSVVFLCFGSYGSFQENQVKEIAM  292

Query  290   ALERSGKRFLWSLRKPIFEGGFAYPTEYENPGEVLPVGFLERTAGVGKVIGWAPQVAVLS  349
              LERSG RFLWSLR  I +G      +Y N  E+LPVGFL+RT+ VGKVIGWAPQVAVL
Sbjct  293   GLERSGHRFLWSLRPSIPKGETKLQLKYSNLKEILPVGFLDRTSCVGKVIGWAPQVAVLG  352

Query  350   HPSVGGFVSHCGWNSTLESVCCGVPMAAWPLGAEQQTNAFQLVKDIGIAVEIKMDYRKN-  408
             H SVGGF+SHCGWNSTLESV CGVP+A WP+  EQQ NAF++VK++GIAVEI++DY+K+
Sbjct  353   HESVGGFLSHCGWNSTLESVWCGVPVATWPMYGEQQLNAFEMVKELGIAVEIEVDYKKDY  412

Query  409   ----SGEIVPKSIIEKAIKQLMDPTN--EIRVRVKELKEKSTRALMEGGSSYNHLGLL  460
                 +  IV    IE  IK+LM   N  EIR +VKE+KEKS  A+ E GSSYN L  L
Sbjct  413   FNMKNDFIVRAEEIETKIKKLMMDENNSEIRKKVKEMKEKSRAAMSENGSSYNSLAKL  470
```

RecName: Full=UDP-glycosyltransferase 71A27; Short=PgUGT71A27 [Panax ginseng]

Sequence ID: **A0A0A7HB61.1** Length: 473 Number of Matches: 1

Range 1: 2 to 468

Score	Expect	Method	Identities	Positives	Gaps	Frame
390 bits(1001)	5e-131()	Compositional matrix adjust.	217/476(46%)	298/476(62%)	30/476(6%)	

```
Query  6    KASLVFIPFPVVSHLVTAVKTAELLASHDSRLSITVLVMSMPTDTKISSYIKN------P  59
            K+ L+F+P P + HLV  V+ A+L  S    LS+TVL+     DT + +Y K+      P
Sbjct  2    KSELIFLPAPAIGHLVGMVEMAKLFISRHENLSVTVLIAKFYMDTGVDNYNKSLLTNPTP  61

Query  60   RINFVQL-EQDVSNGAEAIMKPPKSMM-HFAGRHRDSARAVVSEMKRS--CRVAGIFVDI  115
            R+  V L E D  N     ++KP  ++           +    R ++S M +S   RV G+  D+
Sbjct  62   RLTIVNLPETDPQN---YMLKPRHAIFPSVIETQKTHVRDIISGMTQSESTRVVGLLADL  118

Query  116  MCVDMIDVAKELKISSYIFFASGAAVLGLTFDLQSLRDDGGRNLAEFEGSDEVVSISSYV  175
            + ++++D+A E   + +Y++  +GA  LGL F LQ+L +D  +++ EF  SD  + + S+
Sbjct  119  LFINIMDIANEFNVPTYVYSPAGAGHLGLAFHLQTL-NDKKQDVTEFRNSDTELLVPSFA  177

Query  176  NPVPARVWPESVFDGESGF---LELSRKAREADGIVINTFLELESYAIGSTYANERIPRF  232
            NPVPA V P    D E G+      L R+ RE+  I+INTF ELE YAI S    +  IP
Sbjct  178  NPVPAEVLPSMYVDKEGGYDYLFSLFRRCRESKAIIINTFEELEPYAINSLRMDSMIPPI  237

Query  233  YPIGPII---GEGKDENDESRQRRGEIMRWLDGQPDSSVVFLCFGSMGAFGEEQVVDIAE  289
            YP+GPI+   G+G++ ++ +       I+ WLD QP SSVVFLCFGS G+F E QV +IA
Sbjct  238  YPVGPILNLNGDGQNSDEAAV-----ILGWLDDQPPSSVVFLCFGSYGSFQENQVKEIAM  292

Query  290  ALERSGKRFLWSLRKPIFEGGFAYPTEYENPGEVLPVGFLERTAGVGKVIGWAPQVAVLS  349
             LERSG RFLWSLR  I +G       +Y N  E+LPVGFL+RT+ VGKVIGWAPQVAVL+
Sbjct  293  GLERSGHRFLWSLRPSIPKGETKLQLKYSNLKEILPVGFLDRTSCVGKVIGWAPQVAVLA  352

Query  350  HPSVGGFVSHCGWNSTLESVCCGVPMAAWPLGAEQQTNAFQLVKDIGIAVEIKMDYR---  406
            H +VGGFVSHCGWNS LESV   + +A WP+  EQQ NAF++VK++G+AVEI++DYR
Sbjct  353  HKAVGGFVSHCGWNSILESVWYDMSVATWPMYGEQQLNAFEMVKELGLAVEIEVDYRNEY  412

Query  407  KNSGEIVPKSIIEKAIKQLM--DPTNEIRVRVKELKEKSTRALMEGGSSYNHLGLL  460
              +G IV    IE  IK+LM  +   +EIR +VKE+KEKS  A+ E GSSY  L  L
Sbjct  413  NKTGFIVRADEIETKIKKLMMDEKNSEIRKKVKEMKEKSRVAMSENGSSYTSLAKL  468
```

RecName: Full=UDP-glycosyltransferase 100; Short=UGTPg100 [Panax ginseng]

Sequence ID: **A0A0K0PVW1.1** Length: 472 Number of Matches: 1

Range 1: 2 to 467

Score	Expect	Method	Identities	Positives	Gaps	Frame
385 bits(989)	3e-129()	Compositional matrix adjust.	220/476(46%)	295/476(61%)	31/476(6%)	

```
Query  6    KASLVFIPFPVVSHLVTAVKTAELLASHDSRLSITVLVMSMPTDTKISSYIKN------P  59
            K+ L+F+P P    HLV  V+ A+L  S    LS+TVL+     DT I +Y K+      P
Sbjct  2    KSELIFLPVPAFGHLVGMVEMAKLFISRHENLSVTVLISKFFIDTGIDNYNKSLLAKPTP  61

Query  60   RINFVQLEQDVSNGAEAIMKPPKSMM-HFAGRHRDSARAVVSEMKRS--CRVAGIFVDIM  116
            R+   + L +    +  + ++KP  ++           +    R V+S M +S   RV G+  DI+
Sbjct  62   RLTIINLPE--IDPQKYLLKPRCAIFPSLIENQKTHVRDVMSRMTQSESTRVVGLLADIL  119

Query  117  CVDMIDVAKELKISSYIFFASGAAVLGLTFDLQSLRDDGGRNLAEFEGSDEVVSISSYVN  176
             VD+ D+A E  + +Y++  +GA  LGL F LQ+L DD  +++ EF  SD  + + S+ N
Sbjct  120  FVDIFDIADEFNVPTYVYSPAGAGFLGLAFHLQTLNDDKKQDVTEFRNSDTELLVPSFAN  179

Query  177  PVPARVWPESVFDGESG----FLELSRKAREADGIVINTFLELESYAIGSTYANERIPRF  232
            PVPA   P S+F  + G      L L  + REA GI++NTF ELE YAI S    +  IP
Sbjct  180  PVPAEFLP-SIFLEKDGRHDVLLSLYWRCREAKGIIVNTFEELEPYAINSLRMDSMIPPI  238

Query  233  YPIGPII---GEGKDENDESRQRRGEIMRWLDGQPDSSVVFLCFGSMGAFGEEQVVDIAE  289
            YP+GPI+   GEG++ ++ +       I+ WLD QP SSVVFLCFGS G+F E QV +IA
Sbjct  239  YPVGPILNLNGEGQNSDEAAV-----ILGWLDDQPPSSVVFLCFGSFGSFPENQVKEIAM  293

Query  290  ALERSGKRFLWSLRKPIFEGGFAYPTEYENPGEVLPVGFLERTAGVGKVIGWAPQVAVLS  349
             LERSG RFLWSLR  I EG       +Y N    LP GFL+RT+ VGKVIGWAPQ+A+L+
Sbjct  294  GLERSGHRFLWSLRPCISEGETTLQLKYSNLE--LPAGFLDRTSCVGKVIGWAPQMAILA  351

Query  350  HPSVGGFVSHCGWNSTLESVCCGVPMAAWPLGAEQQTNAFQLVKDIGIAVEIKMDYR---  406
            H +VGGFVSHCGWNS LESV  G+P+A WP+  EQQ NAF++VK++G+AVEI++DYR
Sbjct  352  HEAVGGFVSHCGWNSVLESVWYGMPVATWPMYGEQQLNAFEMVKELGLAVEIEVDYRNEY  411

Query  407  KNSGEIVPKSIIEKAIKQL-MDPTN-EIRVRVKELKEKSTRALMEGGSSYNHLGLL  460
              S  IV    IE  IK+L MD  N +IR +VKE+KEKS  A+ E GSSY  L  L
Sbjct  412  NKSDFIVKADEIETKIKKLMMDGKNSKIRKKVKEMKEKSRVAMSENGSSYTSLAKL  467
```

RecName: Full=UDP-glucosyltransferase 102; Short=UGTPg102 [Panax ginseng]

Sequence ID: **A0A0K0PVL0.1** Length: 475 Number of Matches: 1

Range 1: 2 to 470

Score	Expect	Method	Identities	Positives	Gaps	Frame
384 bits(987)	7e-129()	Compositional matrix adjust.	214/478(45%)	294/478(61%)	32/478(6%)	

```
Query  6    KASLVFIPFPVVSHLVTAVKTAELLASHDSRLSITVLVMSMPTDTKISSYIKN------P  59
            K+ L+F+P P + HLV  V+ A+L  S    LS+TV +     DT + +Y K+      P
Sbjct  2    KSELIFLPAPAIGHLVGMVEMAKLFISRHENLSVTVFISKFYMDTGVDNYNKSLLTNPTP  61

Query  60   RINFVQL-EQDVSNGAEAIMKPPKSMM-HFAGRHRDSARAVVSEMKRS--CRVAGIFVDI  115
            R+  V L E D  N     ++KP  +++          +    R ++S M +S   RV G+  D+
Sbjct  62   RLTIVNLPETDPQN---YMLKPRHAILPSVIETQKTHVRDIISGMTQSESTRVVGLLADL  118

Query  116  MCVDMIDVAKELKISSYIFFASGAAVLGLTFDLQSLRDDGGRNLAEFEGSDEVVSISSYV  175
            + ++++D+A E  +  Y++  +GA  LGL F LQ+L D    +++ EF  SD  + +   +
Sbjct  119  LFINIMDIANEFNVPIYVYSPAGAGYLGLAFHLQTLYDKK-QDVTEFRNSDTELLVPGFA  177

Query  176  NPVPARVWPESVFDGESGF---LELSRKAREADGIVINTFLELESYAIGSTYANERIPRF  232
            NPVPA V P    D E G+      L R+ RE+  I+INTF ELE YAI S    +  IP
Sbjct  178  NPVPAEVLPSMYVDKEGGYDYLFSLFRRCRESKAIIINTFEELEPYAINSLRMDSMIPPI  237

Query  233  YPIGPII---GEGKDENDESRQRRGEIMRWLDGQPDSSVVFLCFGSMGAFGEEQVVDIAE  289
            YP+GPI+   G+G++ ++ +       I+ WLD QP SSVVFLCFGS G F E QV +IA
Sbjct  238  YPVGPILNLNGDGQNSDEAAV-----ILGWLDDQPPSSVVFLCFGSYGTFQENQVKEIAM  292

Query  290  ALERSGKRFLWSLRKPIFEGGFAYPTEYENPGEVLPVGFLERTAGVGKVIGWAPQVAVLS  349
```

```
                 LERSG RFLW+LR  I +G      +Y N  E+LPVGFL+RT+ VGKVIGWAPQVAVL
Sbjct  293  GLERSGHRFLWALRPSIPKGETKLQLKYSNLEEILPVGFLDRTSCVGKVIGWAPQVAVLG  352

Query  350  HPSVGGFVSHCGWNSTLESVCCGVPMAAWPLGAEQQTNAFQLVKDIGIAVEIKMDYRK--  407
            H +V GF+SHCGWNSTLESV  GVP+A WP+  EQ  NAF++VK++G+AVEI++DY+
Sbjct  353  HEAVAGFMSHCGWNSTLESVWFGVPVATWPMYGEQHLNAFEMVKELGLAVEIEVDYKNEY  412

Query  408  ---NSGEIVPKSIIEKAIKQLM--DPTNEIRVRVKELKEKSTRALMEGGSSYNHLGLL  460
                +  IV    IE  IK+LM  +  +EIR +VKE+KEKS  A+ E GSSYN L  L
Sbjct  413  FNTKNDFIVRAEEIETKIKKLMMDEKNSEIRKKVKEMKEKSRVAMSENGSSYNSLAKL  470
```

Taxonomy

Reports

- **Lineage**

Organism	Blast Name	Score	Number of Hits	Description
Pentapetalae	eudicots		10	
.Rosaceae	eudicots		4	
..Fragaria x ananassa	eudicots	442	2	**Fragaria x ananassa hits**
..Malus domestica	eudicots	436	1	**Malus domestica hits**
..Pyrus communis	eudicots	426	1	**Pyrus communis hits**
.Stevia rebaudiana	eudicots	424	1	**Stevia rebaudiana hits**
.Panax ginseng	eudicots	402	5	**Panax ginseng hits**

- **Organism**

Description	Score	E value	Accession
Fragaria x ananassa (strawberry) [eudicots]			
**RecName: Full=UDP-glucose flavonoid 3-O-glucosyltransferase 6; AltName: Full=Flavonol 3-O-glucosyltransferase 6; Short=FaGT6 [Fragaria x ananassa]**	442	2e-151	**Q2V6K0**
**RecName: Full=Putative UDP-glucose flavonoid 3-O-glucosyltransferase 3; Short=FaGT3; AltName: Full=Flavonol 3-O-glucosyltransferase 3 [Fragaria x ananassa]**	431	7e-147	**Q66PF3**
Malus domestica (apple) [eudicots]			
**RecName: Full=UDP-glycosyltransferase 71A15; AltName: Full=UDP-glucose:chalcone 2'-O-glucosyltransferase; AltName: Full=UDP-glucose:flavonol 2'-O-glucosyltransferase [Malus domestica]**	436	5e-149	**D3THI6**
Pyrus communis (pear) [eudicots]			
**RecName: Full=UDP-glycosyltransferase 71A16; AltName: Full=UDP-glucose:chalcone 2'-O-glucosyltransferase; AltName: Full=UDP-glucose:flavonol 2'-O-glucosyltransferase [Pyrus communis]**	426	5e-145	**D3UAG1**
Stevia rebaudiana [eudicots]			
**RecName: Full=UDP-glycosyltransferase 71E1 [Stevia rebaudiana]**	424	2e-144	**Q6VAB2**
Panax ginseng [eudicots]			
**RecName: Full=UDP-glycosyltransferase 101; Short=UGTPg101 [Panax ginseng]**	402	7e-136	**A0A0K0PVM5**
**RecName: Full=UDP-glycosyltransferase 1; Short=UGTPg1 [Panax ginseng]**	402	7e-136	**A0A068J840**
**RecName: Full=UDP-glycosyltransferase 71A27; Short=PgUGT71A27 [Panax ginseng]**	390	5e-131	**A0A0A7HB61**
**RecName: Full=UDP-glycosyltransferase 100; Short=UGTPg100 [Panax ginseng]**	385	3e-129	**A0A0K0PVW1**
**RecName: Full=UDP-glucosyltransferase 102; Short=UGTPg102 [Panax ginseng]**	384	7e-129	**A0A0K0PVL0**

- **Taxonomy**

Taxonomy	Number of hits	Number of Organisms	Description
Pentapetalae	10	5	
. Rosaceae	4	3	
.. Fragaria x ananassa	2	1	**Fragaria x ananassa hits**
.. Maleae	2	2	
... Malus domestica	1	1	**Malus domestica hits**
... Pyrus communis	1	1	**Pyrus communis hits**
. campanulids	6	2	
.. Stevia rebaudiana	1	1	**Stevia rebaudiana hits**
.. Panax ginseng	5	1	**Panax ginseng hits**
