# Supplementary material for: Genome-wide identification and functional characterization of natural antisense transcripts in Salvia miltiorrhiza
Source: Sci Rep. 2021 Feb 26;11:4769. doi: 10.1038/s41598-021-83520-6 (PMC7910453; doi:10.1038/s41598-021-83520-6)
Supplement: Supplementary file 5 — Supplementary Information. [file 41598_2021_83520_MOESM5_ESM.pdf]

COVID-19 is an emerging, rapidly evolving situation.
Get the latest public health information from CDC: https://www.coronavirus.gov .
Get the latest research from NIH: https://www.nih.gov/coronavirus.
Find NCBI SARS-CoV-2 literature, sequence, and content: https://www.ncbi.nlm.nih.gov/sars-cov-2/.

**Job Title**	ST0004 ...
**RID**	MYJ5YKBX014 Search expires on 09-03 15:24 pm
**Program**	BLASTP
**Database**	swissprot
**Query ID**	lcl|Query_70949
**Description**	None ...
**Molecule type**	amino acid
**Query Length**	376

Descriptions

Description	Max Score	Total Score	Query Cover	E value	Per. Ident	Accession
RecName: Full=UDP-glycosyltransferase 90A1 [Arabidopsis thaliana]	201	370	94%	5e-59	47.01%	Q9ZVX4.1
RecName: Full=UDP-glycosyltransferase 90A2 [Arabidopsis thaliana]	181	313	84%	1e-51	47.03%	Q9SY84.1
RecName: Full=UDP-glycosyltransferase 1; Short=PIUGT1; AltName: Full=Glycosyltransferase UGT88E12; AltName: Full=UDP-glucose:isoflavone 7-O-glucosyltransferase KGT1 [Pueraria montana var. lobata]	141	141	61%	1e-36	38.10%	A0A067YB04.1
RecName: Full=Isoflavone 7-O-glucosyltransferase 1; AltName: Full=UDP-glucose:isoflavone 7-O-glucosyltransferase; Flags: Precursor [Glycine max]	137	137	37%	4e-35	46.10%	A6BM07.1
RecName: Full=UDP-glycosyltransferase 2; Short=PIUGT2; AltName: Full=UDP-glucose:isoflavone 4' 7-O-glucosyltransferase [Pueraria montana var. lobata]	135	135	83%	2e-34	31.84%	A0A172J2D0.1
RecName: Full=UDP-glycosyltransferase 13; Short=PIUGT13; AltName: Full=Glycosyltransferase UGT88H1; AltName: Full=UDP-glucose:isoflavone 7-O-glucosyltransferase KGT13 [Pueraria montana var. lobata]	131	131	37%	4e-33	47.22%	A0A067YBQ3.1
RecName: Full=Anthocyanidin 5,3-O-glucosyltransferase; AltName: Full=UDP-glucose: anthocyanidin 5,3-O-glucosyltransferase [Rosa hybrid cultivar]	129	129	37%	2e-32	44.68%	Q4R1I9.1
RecName: Full=Chalcone 4'-O-glucosyltransferase; Short=4'CGT; Short=Am4'CGT [Antirrhinum majus]	127	127	38%	1e-31	44.52%	Q33DV3.1
RecName: Full=UDP-glycosyltransferase 73C1; AltName: Full=Cytokinin-O-glucosyltransferase 1; AltName: Full=Zeatin O-glucosyltransferase 1; Short=AtZOG1 [Arabidopsis thaliana]	124	229	84%	2e-30	42.14%	Q9ZQ99.1
RecName: Full=UDP-glycosyltransferase 73C5; AltName: Full=Cytokinin-O-glucosyltransferase 3; AltName: Full=Deoxynivalenol-glucosyl-transferase 1; AltName: Full=Zeatin O-glucosyltransferase 3; Short=AtZOG3 [Arabidopsis thaliana]	124	231	90%	4e-30	42.17%	Q9ZQ94.1

Graphic Summary

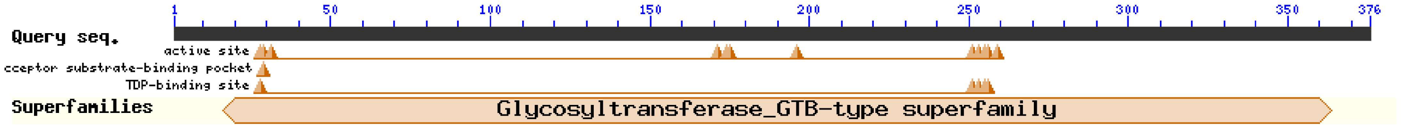

Distribution of the top 14 Blast Hits on 10 subject sequences

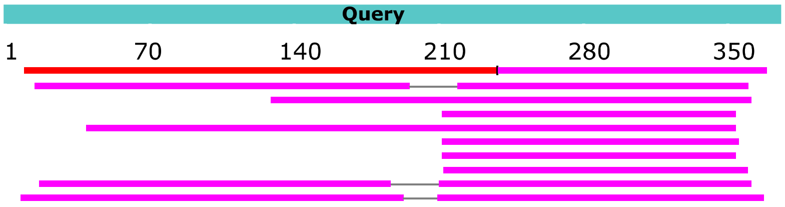

Alignments

Alignment view Pairwise ▼ ☐ CDS feature Restore defaults

RecName: Full=UDP-glycosyltransferase 90A1 [Arabidopsis thaliana]

Sequence ID: **Q9ZVX4.1** Length: 478 Number of Matches: 2

Range 1: 2 to 231

Score	Expect	Method	Identities	Positives	Gaps	Frame
201 bits(511)	5e-59()	Compositional matrix adjust.	110/234(47%)	147/234(62%)	11/234(4%)	

```
Query  11   SSSSSKPNIVVFPFMSKGHTIPLLHLAQLLLDRGLA----TVTIFTTPANHPFISQSLAG  66
            S S+   ++V+FPFMSKGH IPLL   +LLL         TVT+FTTP N PFIS  L+
Sbjct  2    SVSTHHHHVVLFPFMSKGHIIPLLQFGRLLLRHHRKEPTITVTVFTTPKNQPFISDFLSD  61

Query  67   A-HVSIVDLPFPRNIQGVPPGVESTDKLPSMALFIPFVRGLELMQPAFEQELEKIHSQVT  125
               + ++ LPFP NI G+PPGVE+T+KLPSM+LF+PF R  +L+QP FE+ L+ +  +V+
Sbjct  62   TPEIKVISLPFPENITGIPPGVENTEKLPSMSLFVPFTRATKLLQPFFEETLKTL-PKVS  120

Query  126  CIISDGFLPWTLESASRFGIPRLSYYGMSYYSMAVSRDAAFSGLMSSPE--TDDEPFTVK  183
             ++SDGFL WT ESA++F IPR   YGM+ YS AVS       L + PE  +D EP TV
Sbjct  121  FMVSDGFLWWTSESAAKFNIPRFVSYGMNSYSAAVSISVFKHELFTEPESKSDTEPVTVP  180

Query  184  SFPWIQITRNDFDEPGRGELLMGGKEERLELLSDDFQSTVSERGIIVTDWVDQE  237
             FPWI++ + DFD  G  E    G    LEL  D  +ST +  G +V  + + E
Sbjct  181  DFPWIKVKKCDFDH-GTTEPEESGA--ALELSMDQIKSTTTSHGFLVNSFYELE  231
```

Range 2: 306 to 476

Score	Expect	Method	Identities	Positives	Gaps	Frame
169 bits(428)	6e-47()	Compositional matrix adjust.	85/171(50%)	111/171(64%)	1/171(0%)	

```
Query  197  EPGRGELLMGGKEERLELLSDDFQSTVSERGIIVTDWVDQEEILEHPIVQGFLSHCGWNS  256
            E  +   L   +++  E++ + F   + E G+IV DWVDQ EIL H  V+GFLSHCGWNS
Sbjct  306  EDSKVNFLWVTRKDVEEIIGEGFNDRIRESGMIVRDWVDQWEILSHESVKGFLSHCGWNS  365

Query  257  VLDGICAGVPILAWPMMAEQGLNAKLVVEEIKVGLRIGTVDGRSKGFVTANNLKSAVREL  316
              + IC GVP+LAWPMMAEQ LNAK+VVEEIKVG+R+ T DG  KGFVT   L   ++EL
Sbjct  366  AQESICVGVPLLAWPMMAEQPLNAKMVVEEIKVGVRVETEDGSVKGFVTREELSGKIKEL  425

Query  317  MGSGKGKQLRERVKEIAKAAIEATSEG-GSSWNALSNLISEIQREKEINSS  366
            M    GK  R+ VKE +K A  A  EG GSSW  L  ++ E+ + ++ N +
Sbjct  426  MEGETGKTARKNVKEYSKMAKAALVEGTGSSWKNLDMILKELCKSRDSNGA  476
```

RecName: Full=UDP-glycosyltransferase 90A2 [Arabidopsis thaliana]

Sequence ID: **Q9SY84.1** Length: 467 Number of Matches: 2

Range 1: 5 to 188

Score	Expect	Method	Identities	Positives	Gaps	Frame
181 bits(460)	1e-51()	Compositional matrix adjust.	87/185(47%)	127/185(68%)	6/185(3%)	

```
Query  16   KPNIVVFPFMSKGHTIPLLHLAQLLLDRGLA---TVTIFTTPANHPFISQSLAGAHVSIV  72
            K ++V+FP++SKGH IP+L LA+LLL    A   +VT+FTTP N PFI  SL+G   +IV
Sbjct  5    KVHVVLFPYLSKGHMIPMLQLARLLLSHSFAGDISVTVFTTPLNRPFIVDSLSGTKATIV  64

Query  73   DLPFPRNIQGVPPGVESTDKLPSM--ALFIPFVRGLELMQPAFEQELEKIHSQVTCIISD  130
            D+PFP N+  +PPGVE TDKLP++   +LF+PF R  + MQ  FE+EL  +   +V+ ++SD
Sbjct  65   DVPFPDNVPEIPPGVECTDKLPALSSSLFVPFTRATKSMQADFERELMSL-PRVSFMVSD  123

Query  131  GFLPWTLESASRFGIPRLSYYGMSYYSMAVSRDAAFSGLMSSPETDDEPFTVKSFPWIQI  190
            GFL WT ESA + G PRL ++GM+  S  +      + L+S+ +++ EP +V  FPWI++
Sbjct  124  GFLWWTQESARKLGFPRLVFFGMNCASTVICDSVFQNQLLSNVKSETEPVSVPEFPWIKV  183

Query  191  TRNDF  195
             + DF
Sbjct  184  RKCDF  188
```

Range 2: 325 to 462

Score	Expect	Method	Identities	Positives	Gaps	Frame
132 bits(331)	3e-33()	Compositional matrix adjust.	73/141(52%)	93/141(65%)	5/141(3%)	

```
Query  219  FQSTVSERGIIVTD-WVDQEEILEHPIVQGFLSHCGWNSVLDGICAGVPILAWPMMAEQG  277
            F+  V ERG++V D WVDQ +ILEH  V+GFLSHCGWNS+ + IC+ VPILA+P+ AEQ
Sbjct  325  FEERVGERGMMVRDEWVDQRKILEHESVRGFLSHCGWNSLTESICSEVPILAFPLAAEQP  384

Query  278  LNAKLVVEEIKVGLRIGTVDGRSKGFVTANNLKSAVRELMGSGKGKQLRERVKEIAKAAI  337
            LNA LVVEE++V  R+      S+G V    +   V+ELM   KGK+LR  V+   K A
Sbjct  385  LNAILVVEELRVAERVVAA---SEGVVRREEIAEKVKELMEGEKGKELRRNVEAYGKMAK  441

Query  338  EATSEG-GSSWNALSNLISEI  357
            +A  EG GSS   L NLI+E
Sbjct  442  KALEEGIGSSRKNLDNLINEF  462
```

RecName: Full=UDP-glycosyltransferase 1; Short=PlUGT1; AltName: Full=Glycosyltransferase UGT88E12; AltName: Full=UDP-glucose:isoflavone 7-O-glucosyltransferase KGT1 [Pueraria montana var. lobata]

Sequence ID: **A0A067YB04.1** Length: 471 Number of Matches: 1

Range 1: 262 to 470

Score	Expect	Method	Identities	Positives	Gaps	Frame
141 bits(355)	1e-36()	Compositional matrix adjust.	88/231(38%)	129/231(55%)	22/231(9%)	

```
Query  130  DGFLPWTLESASRFGIPRLSYYGMSYYSMAVSRDAAFSGLMSSPETDDEPFTVKSFPWIQ  189
            DG L W L+S     +  LS+  M  +S    R+ A  GL  S +           F W+
Sbjct  262  DGCLSW-LDSQPSHSVVFLSFGSMGRFSRTQLREIAI-GLEKSEQ---------RFLWV-  309

Query  190  ITRNDFDEPGRGELLMGGKEERLELLSDDFQSTVSERGIIVTDWVDQEEILEHPIVQGFL  249
              R++F+E    E          ELL + F      +G+++ DW  Q  IL H  V GF+
Sbjct  310  -VRSEFEEGDSVE-----PPSLDELLPEGFLERTKGKGMVLRDWAPQAAILSHDSVGGFV  363

Query  250  SHCGWNSVLDGICAGVPILAWPMMAEQGLNAKLVVEEIKVGLRIGTVDGRSKGFVTANNL  309
            +HCGWNSVL+ +C GVP++AWP+ AEQ LN  ++VEE+KVGL    V     G V++  L
Sbjct  364  THCGWNSVLEAVCEGVPMVAWPLYAEQKLNKVILVEEMKVGL---AVKQNKDGLVSSTEL  420

Query  310  KSAVRELMGSGKGKQLRERVKEIAKAAIEATSEGGSSWNALSNLISEIQRE  360
               VRELM S +GK++R+++ ++  +A EA ++GGSS  AL+ L+ E+ RE
Sbjct  421  GDRVRELMDSDRGKEIRQKIFKMKMSANEAMAKGGSSIMALNRLV-EVWRE  470
```

RecName: Full=Isoflavone 7-O-glucosyltransferase 1; AltName: Full=UDP-glucose:isoflavone 7-O-glucosyltransferase; Flags: Precursor [Glycine max]

Sequence ID: **A6BM07.1** Length: 474 Number of Matches: 1

Range 1: 330 to 467

Score	Expect	Method	Identities	Positives	Gaps	Frame
137 bits(345)	4e-35()	Compositional matrix adjust.	65/141(46%)	94/141(66%)	3/141(2%)	

```
Query  213  ELLSDDFQSTVSERGIIVTDWVDQEEILEHPIVQGFLSHCGWNSVLDGICAGVPILAWPM  272
            ELL + F     E+G++V DW  Q  IL H  V GF++HCGWNSVL+ +C GVP++AWP+
Sbjct  330  ELLPEGFLERTKEKGMVVRDWAPQAAILSHDSVGGFVTHCGWNSVLEAVCEGVPMVAWPL  389

Query  273  MAEQGLNAKLVVEEIKVGLRIGTVDGRSKGFVTANNLKSAVRELMGSGKGKQLRERVKEI  332
             AEQ +N  ++V+E+KV L    V+    GFV++  L   VRELM S KGK++R+R+ ++
Sbjct  390  YAEQKMNRMVMVKEMKVAL---AVNENKDGFVSSTELGDRVRELMESDKGKEIRQRIFKM  446

Query  333  AKAAIEATSEGGSSWNALSNL  353
              +A EA +EGG+S  +L  L
Sbjct  447  KMSAAEAMAEGGTSRASLDKL  467
```

RecName: Full=UDP-glycosyltransferase 2; Short=PlUGT2; AltName: Full=UDP-glucose:isoflavone 4' 7-O-glucosyltransferase [Pueraria montana var. lobata]

Sequence ID: **A0A172J2D0.1** Length: 472 Number of Matches: 1

Range 1: 137 to 465

Score	Expect	Method	Identities	Positives	Gaps	Frame
135 bits(341)	2e-34()	Compositional matrix adjust.	114/358(32%)	171/358(47%)	74/358(20%)	

```
Query  41   LDRGLATVTIFTTPA-------NHPFISQSLAGAHVSIVDLPFPRNIQGVPPGVESTD--  91
            L++ + T   +T+ A       ++P I ++L   +V   D P    I G+   + + D
Sbjct  137  LNKNIHTYFYYTSGASTLALLLHYPTIHETLTKNYVK--DQPLQIQIPGLRANITTDDFA  194

Query  92   ---KLPSMALFIPFVRGLELMQPAF----------EQELEKIHSQ------VTCI---IS  129
               K PS      F++  E M+ +F          E+EL +  S+      + CI   IS
Sbjct  195  KDSKDPSNYSSQAFLKIAETMRGSFGIIINTFEAIEEELIRALSEDGTVPPLFCIGPVIS  254

Query  130  -------DGFLPWTLESASRFGIPRLSYYGMSYYSMAVSRDAAFSGLMSSPETDDEPFTV  182
                    G L W L+S     +  L +  M  +S    ++ A  GL  S +
Sbjct  255  APYGEDDKGCLSW-LDSQPSQSVVLLCFGSMGSFSRTQLKEIAV-GLEKSEQ--------  304

Query  183  KSFPWIQITRNDFD-------EPGRGELLMGGKEERLELLSDDFQSTVSERGIIVTDWVD  235
              F W+   R + D       +P   EL+ GG  ER             E+G++V DW
Sbjct  305  -RFLWV--VRAELDCADSVDEQPSLDELMPGGFLER-----------TKEKGLVVRDWAP  350

Query  236  QEEILEHPIVQGFLSHCGWNSVLDGICAGVPILAWPMMAEQGLNAKLVVEEIKVGLRIGT  295
            Q +IL H  V GF++HCGWNSVL+ +C GVP+ AWP+ AEQ +N  ++VE++KV L
Sbjct  351  QVQILSHDSVGGFVTHCGWNSVLEAVCEGVPMAAWPLYAEQRVNRVIMVEDMKVAL---A  407

Query  296  VDGRSKGFVTANNLKSAVRELMGSGKGKQLRERVKEIAKAAIEATSEGGSSWNALSNL  353
            V+    GFV+A  L   VRELM S KGK++R+R  ++  +A EA +EGG+S  AL  L
Sbjct  408  VNEDKAGFVSATELGDRVRELMESDKGKEIRQRTFKMKISAAEAMAEGGTSRVALDKL  465
```

RecName: Full=UDP-glycosyltransferase 13; Short=PlUGT13; AltName: Full=Glycosyltransferase UGT88H1; AltName: Full=UDP-glucose:isoflavone 7-O-glucosyltransferase KGT13 [Pueraria montana var. lobata]

Sequence ID: **A0A067YBQ3.1** Length: 451 Number of Matches: 1

Range 1: 305 to 446

Score	Expect	Method	Identities	Positives	Gaps	Frame
131 bits(330)	4e-33()	Compositional matrix adjust.	68/144(47%)	95/144(65%)	4/144(2%)	

```
Query  213  ELLSDDFQSTVSERGIIVTDWVDQEEILEHPIVQGFLSHCGWNSVLDGICAGVPILAWPM  272
            ELL   F     ERG++V +W  Q ++L H  V GF++HCGWNSVL+ +  GVP++AWP+
Sbjct  305  ELLPKGFLERTKERGMVVKNWAPQVKVLSHDSVGGFVTHCGWNSVLEAVSWGVPMVAWPL  364

Query  273  MAEQGLNAKLVVEEIKVGLRIGTVDGRSKGFVTANNLKSAVRELMGS--GKGKQLRERVK  330
             AEQ LN  ++VEE+KV L +  VD    GFV A+ L+  VRELM S  G+GK++R+RV
Sbjct  365  YAEQRLNRVVMVEEMKVALPLKEVD--EDGFVRASELEERVRELMDSERGRGKEVRKRVL  422

Query  331  EIAKAAIEATSEGGSSWNALSNLI  354
                 A+ A S+GGSS   L++L+
```

```
Sbjct  423  GATNDAVAALSDGGSSRIELNDLV  446
```

RecName: Full=Anthocyanidin 5,3-O-glucosyltransferase; AltName: Full=UDP-glucose: anthocyanidin 5,3-O-glucosyltransferase [Rosa hybrid cultivar]

Sequence ID: **Q4R1I9.1** Length: 473 Number of Matches: 1

Range 1: 329 to 466

Score	Expect	Method	Identities	Positives	Gaps	Frame
129 bits(325)	2e-32()	Compositional matrix adjust.	63/141(45%)	88/141(62%)	3/141(2%)	

```
Query  213  ELLSDDFQSTVSERGIIVTDWVDQEEILEHPIVQGFLSHCGWNSVLDGICAGVPILAWPM  272
            E+L   F     +RG++V  W  Q E+L H  V GF++HCGWNSVL+ +C GVP++AWP+
Sbjct  329  EILPKGFVERTKDRGLVVRKWAPQVEVLSHDSVGGFVTHCGWNSVLEAVCNGVPMVAWPL  388

Query  273  MAEQGLNAKLVVEEIKVGLRIGTVDGRSKGFVTANNLKSAVRELMGSGKGKQLRERVKEI  332
             AEQ L    +VEE+KV +    V     GFV+A+ L+  VRELM S  G ++R RV E
Sbjct  389  YAEQKLGRVFLVEEMKVAV---GVKESETGFVSADELEKRVRELMDSESGDEIRGRVSEF  445

Query  333  AKAAIEATSEGGSSWNALSNL  353
            +   ++A  EGGSS  +L+ L
Sbjct  446  SNGGVKAKEEGGSSVASLAKL  466
```

RecName: Full=Chalcone 4'-O-glucosyltransferase; Short=4'CGT; Short=Am4'CGT [Antirrhinum majus]

Sequence ID: **Q33DV3.1** Length: 457 Number of Matches: 1

Range 1: 316 to 457

Score	Expect	Method	Identities	Positives	Gaps	Frame
127 bits(319)	1e-31()	Compositional matrix adjust.	65/146(45%)	90/146(61%)	4/146(2%)	

```
Query  214  LLSDDFQSTVSERGIIVTDWVDQEEILEHPIVQGFLSHCGWNSVLDGICAGVPILAWPMM  273
            LL + F S     G +   WV Q+E+L H  V GF++HCGW+SVL+ +  GVP++ WP+
Sbjct  316  LLPEGFLSRTKGVGFVTNTWVPQKEVLSHDAVGGFVTHCGWSSVLEALSFGVPMIGWPLY  375

Query  274  AEQGLNAKLVVEEIKVGLRIGTVDGRSKGFVTANNLKSAVRELMGSGKGKQLRERVKEIA  333
            AEQ +N   +VEEIKV L +   D    GFVTA  L+  VRELM S KGK+++ RV E+
Sbjct  376  AEQRINRVFMVEEIKVALPLDEED----GFVTAMELEKRVRELMESVKGKEVKRRVAELK  431

Query  334  KAAIEATSEGGSSWNALSNLISEIQR  359
             +   A S+GGSS  +L   I+ + R
Sbjct  432  ISTKAAVSKGGSSLASLEKFINSVTR  457
```

RecName: Full=UDP-glycosyltransferase 73C1; AltName: Full=Cytokinin-O-glucosyltransferase 1; AltName: Full=Zeatin O-glucosyltransferase 1; Short=AtZOG1 [Arabidopsis thaliana]

Sequence ID: **Q9ZQ99.1** Length: 491 Number of Matches: 2

Range 1: 327 to 485

Score	Expect	Method	Identities	Positives	Gaps	Frame
124 bits(311)	2e-30()	Compositional matrix adjust.	67/159(42%)	97/159(61%)	9/159(5%)	

```
Query  210  ERLELLSDD-FQSTVSERGIIVTDWVDQEEILEHPIVQGFLSHCGWNSVLDGICAGVPIL  268
            E LE +S+  ++  + ERG+++T W  Q  IL HP V GFL+HCGWNS L+GI +GVP+L
Sbjct  327  ELLEWISESGYKERIKERGLLITGWSPQMLILTHPAVGGFLTHCGWNSTLEGITSGVPLL  386

Query  269  AWPMMAEQGLNAKLVVEEIKVGLRIGTVDGRSKG-------FVTANNLKSAVRELMG-SG  320
             WP+  +Q  N KL V+ +K G+R G  +    G        V    +K AV ELMG S
Sbjct  387  TWPLFGDQFCNEKLAVQILKAGVRAGVEESMRWGEEEKIGVLVDKEGVKKAVEELMGDSN  446

Query  321  KGKQLRERVKEIAKAAIEATSEGGSSWNALSNLISEIQR  359
              K+ R+RVKE+ + A +A  EGGSS + ++ L+ +I +
Sbjct  447  DAKERRKRVKELGELAHKAVEEGGSSHSNITFLLQDIMQ  485
```

Range 2: 10 to 183

Score	Expect	Method	Identities	Positives	Gaps	Frame
104 bits(260)	2e-23()	Compositional matrix adjust.	57/175(33%)	98/175(56%)	7/175(4%)	

```
Query  18   NIVVFPFMSKGHTIPLLHLAQLLLDRGLATVTIFTTPANHPFISQSLA-----GAHVSIV  72
            + V+FPFM++GH IP++ +A+LL  RG+ T+TI TTP N       L+     G  +++V
Sbjct  10   HFVLFPFMAQGHMIPMVDIARLLAQRGV-TITIVTTPQNAGRFKNVLSRAIQSGLPINLV  68

Query  73   DLPFPRNIQGVPPGVESTDKLPSMALFIPFVRGLELMQPAFEQELEKIHSQVTCIISDGF  132
             + FP    G P G E+ D L S+   + F +   L++   E+ L++I  +  CII+D
Sbjct  69   QVKFPSQESGSPEGQENLDLLDSLGASLTFFKAFSLLEEPVEKLLKEIQPRPNCIIADMC  128

Query  133  LPWTLESASRFGIPRLSYYGMSYYSMAVSRDAAFSG-LMSSPETDDEPFTVKSFP  186
            LP+T   A   GIP++ ++GM  +++  +     +   + + E+D E F + +FP
Sbjct  129  LPYTNRIAKNLGIPKIIFHGMCCFNLLCTHIMHQNHEFLETIESDKEYFPIPNFP  183
```

RecName: Full=UDP-glycosyltransferase 73C5; AltName: Full=Cytokinin-O-glucosyltransferase 3; AltName: Full=Deoxynivalenol-glucosyl-transferase 1; AltName: Full=Zeatin O-glucosyltransferase 3; Short=AtZOG3 [Arabidopsis thaliana]

Sequence ID: **Q9ZQ94.1** Length: 495 Number of Matches: 2

Range 1: 330 to 495

Score	Expect	Method	Identities	Positives	Gaps	Frame
124 bits(310)	4e-30()	Compositional matrix adjust.	70/166(42%)	97/166(58%)	9/166(5%)	

```
Query  209  EERLELLSDD-FQSTVSERGIIVTDWVDQEEILEHPIVQGFLSHCGWNSVLDGICAGVPI  267
            +E +E  S+  F+  + +RG+++  W  Q  IL HP V GFL+HCGWNS L+GI AG+P+
Sbjct  330  KELVEWFSESGFEDRIQDRGLLIKGWSPQMLILSHPSVGGFLTHCGWNSTLEGITAGLPL  389

Query  268  LAWPMMAEQGLNAKLVVEEIKVGLRIGTVDGRSKG-------FVTANNLKSAVRELMG-S  319
            L WP+ A+Q  N KLVVE +K G+R G       G        V    +K AV ELMG S
Sbjct  390  LTWPLFADQFCNEKLVVEVLKAGVRSGVEQPMKWGEEEKIGVLVDKEGVKKAVEELMGES  449

Query  320  GKGKQLRERVKEIAKAAIEATSEGGSSWNALSNLISEIQREKEINS  365
               K+ R R KE+  +A +A  EGGSS + +S L+ +I    E N+
Sbjct  450  DDAKERRRRAKELGDSAHKAVEEGGSSHSNISFLLQDIMELAEPNN  495
```

Range 2: 2 to 192

Score	Expect	Method	Identities	Positives	Gaps	Frame
107 bits(268)	2e-24()	Compositional matrix adjust.	63/194(32%)	112/194(57%)	13/194(6%)	

```
Query  9    ISSSSSSKP-NIVVFPFMSKGHTIPLLHLAQLLLDRGLATVTIFTTPANHPFISQSL---  64
            +S ++ S P + V+FPFM++GH IP++ +A+LL  RG+  +TI TTP N       L
Sbjct  2    VSETTKSSPLHFVLFPFMAQGHMIPMVDIARLLAQRGV-IITIVTTPHNAARFKNVLNRA  60

Query  65   --AGAHVSIVDLPFPRNIQGVPPGVESTDKLPSMALFIPFVRGLELMQPAFEQELEKIHS  122
              +G  +++V + FP    G+  G E+ D L +M   IPF + +  ++   ++ +E+++
Sbjct  61   IESGLPINLVQVKFPYLEAGLQEGQENIDSLDTMERMIPFFKAVNFLEEPVQKLIEEMNP  120

Query  123  QVTCIISDGFLPWTLESASRFGIPRLSYYGMSYY---SMAVSRDAAFSGLMSSPETDDEP  179
            + +C+ISD  LP+T + A +F IP++ ++GM  +    M V R      ++ + ++D E
Sbjct  121  RPSCLISDFCLPYTSKIAKKFNIPKILFHGMGCFCLLCMHVLRKN--REILDNLKSDKEL  178

Query  180  FTVKSFP-WIQITR  192
            FTV  FP  ++ TR
Sbjct  179  FTVPDFPDRVEFTR  192
```

Taxonomy

Reports

- **Lineage**

Organism	Blast Name	Score	Number of Hits	Description
Pentapetalae	eudicots		10	
.rosids	eudicots		9	
..Arabidopsis thaliana	eudicots	201	4	Arabidopsis thaliana hits
..Pueraria montana var. lobata	eudicots	141	3	Pueraria montana var. lobata hits
..Glycine max	eudicots	137	1	Glycine max hits
..Rosa hybrid cultivar	eudicots	129	1	Rosa hybrid cultivar hits
.Antirrhinum majus	eudicots	127	1	Antirrhinum majus hits

- **Organism**

Description	Score	E value	Accession
Arabidopsis thaliana (thale cress) [eudicots]			
RecName: Full=UDP-glycosyltransferase 90A1 [Arabidopsis thaliana]	201	5e-59	Q9ZVX4
RecName: Full=UDP-glycosyltransferase 90A2 [Arabidopsis thaliana]	181	1e-51	Q9SY84
RecName: Full=UDP-glycosyltransferase 73C1; AltName: Full=Cytokinin-O-glucosyltransferase 1; AltName: Full=Zeatin O-glucosyltransferase 1; Short=AtZOG1 [Arabidopsis thaliana]	124	2e-30	Q9ZQ99
RecName: Full=UDP-glycosyltransferase 73C5; AltName: Full=Cytokinin-O-glucosyltransferase 3; AltName: Full=Deoxynivalenol-glucosyl-transferase 1; AltName: Full=Zeatin O-glucosyltransferase 3; Short=AtZOG3 [Arabidopsis thaliana]	124	4e-30	Q9ZQ94
Pueraria montana var. lobata (kudzu vine) [eudicots]			
RecName: Full=UDP-glycosyltransferase 1; Short=PlUGT1; AltName: Full=Glycosyltransferase UGT88E12; AltName: Full=UDP-glucose:isoflavone 7-O-glucosyltransferase KGT1 [Pueraria montana var. lobata]	141	1e-36	A0A067YB04
RecName: Full=UDP-glycosyltransferase 2; Short=PlUGT2; AltName: Full=UDP-glucose:isoflavone 4' 7-O-glucosyltransferase [Pueraria montana var. lobata]	135	2e-34	A0A172J2D0
RecName: Full=UDP-glycosyltransferase 13; Short=PlUGT13; AltName: Full=Glycosyltransferase UGT88H1; AltName: Full=UDP-glucose:isoflavone 7-O-glucosyltransferase KGT13 [Pueraria montana var. lobata]	131	4e-33	A0A067YBQ3
Glycine max (soybean) [eudicots]			
RecName: Full=Isoflavone 7-O-glucosyltransferase 1; AltName: Full=UDP-glucose:isoflavone 7-O-glucosyltransferase; Flags: Precursor [Glycine max]	137	4e-35	A6BM07
