# Supplementary material for: Genome-wide identification and functional characterization of natural antisense transcripts in Salvia miltiorrhiza
Source: Sci Rep. 2021 Feb 26;11:4769. doi: 10.1038/s41598-021-83520-6 (PMC7910453; doi:10.1038/s41598-021-83520-6)
Supplement: Supplementary file 6 — Supplementary Information. [file 41598_2021_83520_MOESM6_ESM.pdf]

COVID-19 is an emerging, rapidly evolving situation.
Get the latest public health information from CDC: https://www.coronavirus.gov .
Get the latest research from NIH: https://www.nih.gov/coronavirus.
Find NCBI SARS-CoV-2 literature, sequence, and clinical content: https://www.ncbi.nlm.nih.gov/sars-cov-2/.

BLAST ® » **blastp suite** » **results for RID-MYJVXRND014**

**Job Title**	ST0023 ...
**RID**	MYJVXRND014 Search expires on 09-03 15:36 pm
**Program**	BLASTP
**Database**	swissprot
**Query ID**	lcl|Query_77003
**Description**	None ...
**Molecule type**	amino acid
**Query Length**	498

Descriptions

Description	Max Score	Total Score	Query Cover	E value	Per. Ident	Accession
RecName: Full=Beta-glucosidase 40; Short=AtBGLU40; Flags: Precursor [Arabidopsis thaliana]	702	702	96%	0.0	66.18%	Q9FZE0.1
RecName: Full=Beta-glucosidase 6; Short=Os3bglu6; Flags: Precursor [Oryza sativa Japonica Group]	700	700	96%	0.0	64.58%	Q8L7J2.1
RecName: Full=Beta-glucosidase 34; Short=Os10bglu34; Flags: Precursor [Oryza sativa Japonica Group]	687	687	99%	0.0	62.12%	Q339X2.1
RecName: Full=Beta-glucosidase 25; Short=Os6bglu25; Flags: Precursor [Oryza sativa Japonica Group]	607	607	95%	0.0	59.12%	Q0DA21.2
RecName: Full=Putative beta-glucosidase 41; Short=AtBGLU41; Flags: Precursor [Arabidopsis thaliana]	600	600	95%	0.0	58.16%	Q9FIU7.2
RecName: Full=Beta-glucosidase 11; Short=Os4bglu11; Flags: Precursor [Oryza sativa Japonica Group]	544	544	96%	0.0	54.64%	Q7XKV5.2
RecName: Full=Beta-glucosidase 12; Flags: Precursor [Oryza sativa Indica Group]	540	540	97%	0.0	55.74%	B8AVF0.1
RecName: Full=Beta-glucosidase 12; Short=Os4bglu12; Flags: Precursor [Oryza sativa Japonica Group]	538	538	97%	0.0	55.74%	Q7XKV4.2
RecName: Full=Beta-glucosidase 13; Short=Os4bglu13; Flags: Precursor [Oryza sativa Japonica Group]	536	536	97%	0.0	56.10%	Q7XKV2.2
RecName: Full=Coniferin beta-glucosidase; Flags: Precursor [Pinus contorta]	523	523	95%	0.0	53.25%	Q9ZT64.1

Graphic Summary

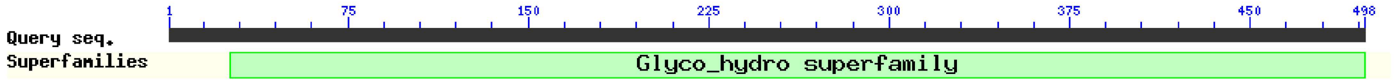

Distribution of the top 10 Blast Hits on 10 subject sequences

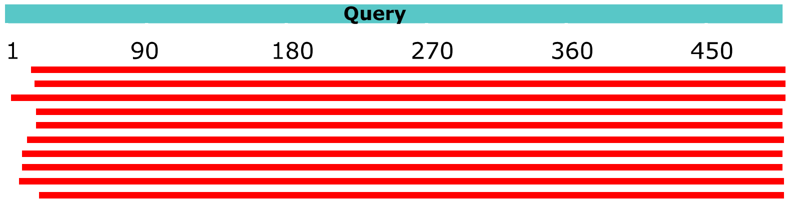

Alignments

Alignment view Pairwise ▼ ☐ CDS feature Restore defaults

RecName: Full=Beta-glucosidase 40; Short=AtBGLU40; Flags: Precursor [Arabidopsis thaliana]
Sequence ID: **Q9FZE0.1** Length: 510 Number of Matches: 1
Range 1: 27 to 508

Score	Expect	Method	Identities	Positives	Gaps	Frame
702 bits(1813)	0.0()	Compositional matrix adjust.	319/482(66%)	393/482(81%)	1/482(0%)	

```
Query  18   CFGQNISRASFPKGFVFGTASSAYQYEGAVSEDGRGPTIWDKFSHEFGKVIDFSNADVAD  77
            C    +ISR SFPKGFVFGTASSA+Q+EGAV   +GRGPTIWD FSH FGK+ DFSNADVA
```

```
Sbjct  27    CICADISRGSFPKGFVFGTASSAFQHEGAVKAEGRGPTIWDTFSHTFGKITDFSNADVAV  86

Query  78    DQYHLYDDDIHTMKGIGLDAYRFSIAWSRIYPKGSGEINQAGIEHYHNVIDALLAQGIQP  137
             DQYH Y++D+  MK +G+DAYRFSI+W+RI+P G G IN+AGI+HY+ +I+ALLA+GI+P
Sbjct  87    DQYHRYEEDVQLMKNMGMDAYRFSISWTRIFPNGVGHINEAGIDHYNKLINALLAKGIEP  146

Query  138   YVTLFHWDLPQKLEDKYNGFLHPQIIKDFTAYAETCFKEFGDKVKHWITLNEPHTFAVQG  197
             YVTL+HWDLPQ L D+Y G+L+PQII DF AYAE CF+ FGD+VKHWIT NEPHTFA+QG
Sbjct  147   YVTLYHWDLPQALHDRYLGWLNPQIINDFAAYAEVCFQRFGDRVKHWITFNEPHTFAIQG  206

Query  198   YDVGLQAPGHCSILLGAFCRIGNSATEPYIVGHNMLLAHASAVDVYKKKYQPKQKGSIGI  257
             YDVGLQAPG C+IL    CR GNS+TEPYIVGHN++L HA+  D+Y+KKY+ KQ GS+GI
Sbjct  207   YDVGLQAPGRCTILFKLTCREGNSSTEPYIVGHNVILTHATVSDIYRKKYKAKQGGSLGI  266

Query  258   SLDSFWYEPASDSPEDVQATQRAIDFNLGWFLEPLIKGDYPKSMRSRVRERLPRFSVEQS  317
             + D  W+EP S+  ED++A QRA DF LGWFL+PL+ GDYP SMRSRV  RLP F+  QS
Sbjct  267   AFDVMWFEPESNKTEDIEAAQRAQDFQLGWFLDPLMFGDYPSSMRSRVGSRLPVFTGSQS  326

Query  318   VMLRGSHDFIGINHYTTWYAQRDTTNIIGVLLNDSLADSGAFTFPIALGKPVYERANSVW  377
              +++GS DF+GINHYTT+YA+ + TN+IG LL+D+++DSG  T P      + +RA+S+W
Sbjct  327   SLVKGSLDFVGINHYTTYYARNNATNLIGTLLHDAVSDSGTVTLPFKGLSTIGDRASSIW  386

Query  378   LYIVPHGIRSLMNYIRKEYGNPTVIITENGMDDGNNPLTPIKEALKDVKRIKYHHDYLTN  437
             LYIVP G+RSLMNYI+  YGNP V ITENGMDD N+ L   K+ALKD KRIKYHHDYL++
Sbjct  387   LYIVPRGMRSLMNYIKHRYGNPPVFITENGMDDPNSILISRKDALKDAKRIKYHHDYLSS  446

Query  438   LNEAIK-DGCNVKGYFVWSMLDNWEWGAGYTSRFGLYYVDYKNKNQRYPKDSATWFKNFL  496
             L  +IK DGCNVKGYFVWS+LDNWEW AGY+SRFGLY+VDY++  +RYPKDS  WF +FL
Sbjct  447   LQASIKEDGCNVKGYFVWSLLDNWEWAAGYSSRFGLYFVDYRDNLKRYPKDSVHWFTSFL  506

Query  497   KS  498
              S
Sbjct  507   NS  508
```

RecName: Full=Beta-glucosidase 6; Short=Os3bglu6; Flags: Precursor [Oryza sativa Japonica Group]

Sequence ID: **Q8L7J2.1** Length: 521 Number of Matches: 1

Range 1: 42 to 521

Score	Expect	Method	Identities	Positives	Gaps	Frame
700 bits(1806)	0.0()	Compositional matrix adjust.	310/480(65%)	391/480(81%)	1/480(0%)	

```
Query  20    GQNISRASFPKGFVFGTASSAYQYEGAVSEDGRGPTIWDKFSHEFGKVIDFSNADVADDQ  79
             G  ++R SFP+GFVFGTAS+AYQYEGAV EDGRG TIWD F+H FGK+ DFSNADVA DQ
Sbjct  42    GGGLTRGSFPEGFVFGTASAAYQYEGAVKEDGRGQTIWDTFAHTFGKITDFSNADVAVDQ  101

Query  80    YHLYDDDIHTMKGIGLDAYRFSIAWSRIYPKGSGEINQAGIEHYHNVIDALLAQGIQPYV  139
             YH +++DI  M  +G+DAYRFSIAWSRIYP G G++NQAGI+HY+ +IDALLA+GIQPYV
Sbjct  102   YHRFEEDIQLMADMGMDAYRFSIAWSRIYPNGVGQVNQAGIDHYNKLIDALLAKGIQPYV  161

Query  140   TLFHWDLPQKLEDKYNGFLHPQIIKDFTAYAETCFKEFGDKVKHWITLNEPHTFAVQGYD  199
             TL+HWDLPQ LEDKY G+L  QI+ DF AYAETCF+EFGD+VKHWITLNEPHT A+QGYD
Sbjct  162   TLYHWDLPQALEDKYKGWLDRQIVDDFAAYAETCFREFGDRVKHWITLNEPHTVAIQGYD  221

Query  200   VGLQAPGHCSILLGAFCRIGNSATEPYIVGHNMLLAHASAVDVYKKKYQPKQKGSIGISL  259
              GLQAPG CS+LL  +C+ GNS TEPY+V H+ +LAHA+A  +Y+ KY+  Q G +GI+
Sbjct  222   AGLQAPGRCSVLLHLYCKAGNSGTEPYVVAHHFILAHAAASIYRTKYKATQNGQLGIAF  281

Query  260   DSFWYEPASDSPEDVQATQRAIDFNLGWFLEPLIKGDYPKSMRSRVRERLPRFSVEQSVM  319
             D  W+EP S++  D++A +RA +F LGWF +P   GDYP +MR+RV ERLPRF+ +++ +
Sbjct  282   DVMWFEPMSNTTIDIEAAKRAQEFQLGWFADPFFFGDYPATMRARVGERLPRFTADEAAV  341

Query  320   LRGSHDFIGINHYTTWYAQRDTTNIIGVLLNDSLADSGAFTFPIALGKPVYERANSVWLY  379
             ++G+ DF+GINHYTT+Y + + TNIIG LLN++LAD+G  + P  GKP+ +RANS+WLY
Sbjct  342   VKGALDFVGINHYTTYYTRHNNTNIIGTLLNNTLADTGTVSLPFKNGKPIGDRANSIWLY  401

Query  380   IVPHGIRSLMNYIRKEYGNPTVIITENGMDDGNNPLTPIKEALKDVKRIKYHHDYLTNLN  439
             IVP G+RSLMNY+++ Y +P V ITENGMDD NNP   IK+ALKD KRIKYH+DYLTNL
Sbjct  402   IVPRGMRSLMNYVKERYNSPPVYITENGMDDSNNPFISIKDALKDSKRIKYHNDYLTNLA  461

Query  440   EAIK-DGCNVKGYFVWSMLDNWEWGAGYTSRFGLYYVDYKNKNQRYPKDSATWFKNFLKS  498
              +IK DGC+V+GYF WS+LDNWEW AGY+SRFGLY+VDYK+  +RYPK+S  WFK  LK+
Sbjct  462   ASIKEDGCDVRGYFAWSLLDNWEWAAGYSSRFGLYFVDYKDNLKRYPKNSVQWFKALLKT  521
```

RecName: Full=Beta-glucosidase 34; Short=Os10bglu34; Flags: Precursor [Oryza sativa Japonica Group]

Sequence ID: **Q339X2.1** Length: 510 Number of Matches: 1

Range 1: 11 to 508

Score	Expect	Method	Identities	Positives	Gaps	Frame
687 bits(1773)	0.0()	Compositional matrix adjust.	310/499(62%)	390/499(78%)	6/499(1%)	

```
Query  5     IFMIFVAAVGVPPCFGQN----ISRASFPKGFVFGTASSAYQYEGAVSEDGRGPTIWDKF  60
             + ++ V       C  QN    ++R SFP GFVFGTASSAYQYEGAV EDGRGPTIWDKF
Sbjct  11    VVILLVLMAMSQGCDAQNTTGGLTRKSFPNGFVFGTASSAYQYEGAVKEDGRGPTIWDKF  70

Query  61    SHEFGKVIDFSNADVADDQYHLYDDDIHTMKGIGLDAYRFSIAWSRIYPKGSGEINQAGI  120
             +H FGK+IDFSNADVA DQYH +++DI  M  +G+DAYRFSI+WSRI+P G+GE+NQAGI
Sbjct  71    AHTFGKIIDFSNADVAVDQYHRFEEDIQLMADMGMDAYRFSISWSRIFPNGTGEVNQAGI  130

Query  121   EHYHNVIDALLAQGIQPYVTLFHWDLPQKLEDKYNGFLHPQIIKDFTAYAETCFKEFGDK  180
             +HY+ +I+ALLA+GI+PYVTL+HWDLPQ LEDKY G+L  QII D+  YAETCF+ FGD+
Sbjct  131   DHYNKLINALLAKGIEPYVTLYHWDLPQALEDKYTGWLDRQIINDYAVYAETCFQAFGDR  190

Query  181   VKHWITLNEPHTFAVQGYDVGLQAPGHCSILLGAFCRIGNSATEPYIVGHNMLLAHASAV  240
             VKHWIT NEPHT AVQ YD G+ APG CS+LL  +C+ GNS TEPYIV HNM+L+HA+
Sbjct  191   VKHWITFNEPHTVAVQAYDSGMHAPGRCSVLLHLYCKKGNSGTEPYIVAHNMILSHATVS  250

Query  241   DVYKKKYQPKQKGSIGISLDSFWYEPASDSPEDVQATQRAIDFNLGWFLEPLIKGDYPKS  300
             D+Y+KKY+  Q G +GIS D  WYEP S+S  D++A +RA +F LGWF +P   GDYP +
Sbjct  251   DIYRKKYKASQNGELGISFDVIWYEPMSNSTADIEAAKRAQEFQLGWFADPFFFGDYPAT  310
```

```
Query  301  MRSRVRERLPRFSVEQSVMLRGSHDFIGINHYTTWYAQRDTTNIIGVLLNDSLADSGAFT  360
            MRSRV  RLP+F+ +++ ++ GS DF+GINHYTT+Y + D + +I  LLN++LAD+   +
Sbjct  311  MRSRVGSRLPKFTEKEAALVNGSLDFMGINHYTTFYTKDDQSTVIEKLLNNTLADTATIS  370

Query  361  FPIALGKPVYERANSVWLYIVPHGIRSLMNYIRKEYGNPTVIITENGMDDGNNPLTPIKE  420
             P   G+P+ +RANS+WLYIVP  +R LMNY++  Y  PTV ITENGMDDGN+P   +K
Sbjct  371  VPFRNGQPIGDRANSIWLYIVPRSMRILMNYVKDRYNKPTVYITENGMDDGNSPFISLKN  430

Query  421  ALKDVKRIKYHHDYLTNLNEAIK-DGCNVKGYFVWSMLDNWEWGAGYTSRFGLYYVDYKN  479
            ALKD KR KYH+DYLTNL ++I+ DGC+V+GYF WS+LDNWEW AGYTSRFGLYYVDYKN
Sbjct  431  ALKDDKRTKYHNDYLTNLADSIREDGCDVRGYFAWSLLDNWEWAAGYTSRFGLYYVDYKN  490

Query  480  KNQRYPKDSATWFKNFLKS  498
            + +RYPK+S  WFKN L S
Sbjct  491  R-KRYPKNSVQWFKNLLAS  508
```

RecName: Full=Beta-glucosidase 25; Short=Os6bglu25; Flags: Precursor [Oryza sativa Japonica Group]

Sequence ID: **Q0DA21.2** Length: 501 Number of Matches: 1

Range 1: 18 to 494

Score	Expect	Method	Identities	Positives	Gaps	Frame
607 bits(1564)	0.0()	Compositional matrix adjust.	282/477(59%)	362/477(75%)	1/477(0%)	

```
Query  21   QNISRASFPKGFVFGTASSAYQYEGAVSEDGRGPTIWDKFSHEFGKVIDFSNADVADDQY  80
            + ISRA FP GF+FGTASSAYQYEGAV+E  RGPTIWD  +   G+VIDFSNADVA D Y
Sbjct  18   EAISRADFPPGFIFGTASSAYQYEGAVNEGQRGPTIWDTLTKRPGRVIDFSNADVAVDHY  77

Query  81   HLYDDDIHTMKGIGLDAYRFSIAWSRIYPKGSGEINQAGIEHYHNVIDALLAQGIQPYVT  140
            H Y +D+  M  IG+DAYRFSI+WSRI+P G+GE N+ G+ +Y+++IDALL +GI+PYVT
Sbjct  78   HRYKEDVELMNDIGMDAYRFSISWSRIFPNGTGEPNEEGLSYYNSLIDALLDKGIEPYVT  137

Query  141  LFHWDLPQKLEDKYNGFLHPQIIKDFTAYAETCFKEFGDKVKHWITLNEPHTFAVQGYDV  200
            LFHWDLPQ LED+Y G+L+ +II+DF  YA TCFKEFGD+VKHWIT NEP+ FA+ GYD+
Sbjct  138  LFHWDLPQALEDRYGGWLNSEIIEDFVQYAFTCFKEFGDRVKHWITFNEPYNFAIDGYDL  197

Query  201  GLQAPGHCSILLGAFCRIGNSATEPYIVGHNMLLAHASAVDVYKKKYQPKQKGSIGISLD  260
            G+QAPG CSIL   FCR G S+TEPYIV HN+LLAHA A   Y++ ++ +Q G IGI+L+
Sbjct  198  GIQAPGRCSILSHVFCREGKSSTEPYIVAHNILLAHAGAFRAYEQHFKNEQGGLIGIALN  257

Query  261  SFWYEPASDSPEDVQATQRAIDFNLGWFLEPLIKGDYPKSMRSRVRERLPRFSVEQSVML  320
            S WYEP S++ ED +A  RA+DF LGWFL+PL+ G YP SM+    +RLP+FS   S ++
Sbjct  258  SRWYEPFSNADEDTEAAARAMDFELGWFLDPLMFGHYPPSMQKLAGDRLPQFSTHASKLV  317

Query  321  RGSHDFIGINHYTTWYAQRDTTNIIGVLLNDSLADSGAFTFPIALGKPVYERANSVWLYI  380
             GS DF+GINHYTT YA+ D   I  ++++D+  DS         GK + E A S WL+I
Sbjct  318  SGSLDFVGINHYTTLYARNDRLRIRKLVMDDASTDSAVIPTAYRHGKKIGETAASSWLHI  377

Query  381  VPHGIRSLMNYIRKEYGNPTVIITENGMDDGNNPLTPIKEALKDVKRIKYHHDYLTNLNE  440
            VP G+  LM +++++YGNP V+ITENGMDD N+P + +++ L+D KRI+YH+DY++NL +
Sbjct  378  VPWGMFKLMKHVKEKYGNPPVVITENGMDDANHPFSRLEDVLQDDKRIQYHNDYMSNLLD  437

Query  441  AI-KDGCNVKGYFVWSMLDNWEWGAGYTSRFGLYYVDYKNKNQRYPKDSATWFKNFL  496
            AI K+GCNV GYFVWS+LDNWEW +GYT RFGLYY+DYKN   R PK S  WF   L
Sbjct  438  AIRKEGCNVHGYFVWSLLDNWEWNSGYTVRFGLYYIDYKNNLTRIPKASVQWFSQVL  494
```

RecName: Full=Putative beta-glucosidase 41; Short=AtBGLU41; Flags: Precursor [Arabidopsis thaliana]

Sequence ID: **Q9FIU7.2** Length: 535 Number of Matches: 1

Range 1: 28 to 505

Score	Expect	Method	Identities	Positives	Gaps	Frame
600 bits(1547)	0.0()	Compositional matrix adjust.	278/478(58%)	362/478(75%)	2/478(0%)	

```
Query  21   QNISRASFPKGFVFGTASSAYQYEGAVSEDGRGPTIWDKFSHEF-GKVIDFSNADVADDQ  79
            ++ISRA+FP GFVFGTASSAYQ+EGAV E  +G +IWD F+ E  GK++DFSNAD   DQ
Sbjct  28   ESISRANFPDGFVFGTASSAYQFEGAVKEGNKGESIWDTFTKEKPGKILDFSNADTTVDQ  87

Query  80   YHLYDDDIHTMKGIGLDAYRFSIAWSRIYPKGSGEINQAGIEHYHNVIDALLAQGIQPYV  139
            YH + +DI  MK + +DAYRFSI+WSRI+P G+GE+N  G+++Y+++IDALLA+GI+PYV
Sbjct  88   YHRFHNDIDLMKDLRMDAYRFSISWSRIFPNGTGEVNPDGVKYYNSLIDALLAKGIKPYV  147

Query  140  TLFHWDLPQKLEDKYNGFLHPQIIKDFTAYAETCFKEFGDKVKHWITLNEPHTFAVQGYD  199
            TL+HWDLPQ LED+Y G+L  +++ DF  YA TCFK FGD+VK+WIT NEPH  ++QGYD
Sbjct  148  TLYHWDLPQALEDRYEGWLSREVVDDFEHYAFTCFKAFGDRVKYWITFNEPHGVSIQGYD  207

Query  200  VGLQAPGHCSILLGAFCRIGNSATEPYIVGHNMLLAHASAVDVYKKKYQPKQKGSIGISL  259
             G+QAPG CS+L   FC+ G S+ EPYIV HN+LL+HA+A   Y++ ++ KQ+G IGISL
Sbjct  208  TGIQAPGRCSLLGHWFCKKGKSSVEPYIVAHNILLSHAAAYHTYQRNFKEKQRGQIGISL  267

Query  260  DSFWYEPASDSPEDVQATQRAIDFNLGWFLEPLIKGDYPKSMRSRVRERLPRFSVEQSVM  319
            D+ WYEP SD  ED  A +RA+DF LGWF++PLI GDYP SM+S V ERLP+ + E
Sbjct  268  DAKWYEPMSDCDEDKDAARRAMDFGLGWFMDPLINGDYPASMKSLVEERLPKITPEMYKT  327

Query  320  LRGSHDFIGINHYTTWYAQRDTTNIIGVLLNDSLADSGAFTFPIALGKPVYERANSVWLY  379
            ++G+ D++GINHYTT YA+ D T I  ++L D+ +DS   T     G  + ERA S WL+
Sbjct  328  IKGAFDYVGINHYTTLYARNDRTRIRKLILQDASSDSAVITSSFRGGVAIGERAGSSWLH  387

Query  380  IVPHGIRSLMNYIRKEYGNPTVIITENGMDDGNNPLTPIKEALKDVKRIKYHHDYLTNLN  439
            IVP GIR L  Y++  YGNP V ITENGMD+ N+P   +++ALKD KRI +H DYL+NL+
Sbjct  388  IVPWGIRKLAVYVKDIYGNPPVFITENGMDEKNSPFIDMEKALKDDKRIGFHRDYLSNLS  447

Query  440  EAIK-DGCNVKGYFVWSMLDNWEWGAGYTSRFGLYYVDYKNKNQRYPKDSATWFKNFL  496
             AI+ D C+V+GYFVWS+LDNWEW +GYT RFG+YYVDYKN   R PK SA WF+  L
Sbjct  448  AAIRNDECDVRGYFVWSLLDNWEWNSGYTVRFGIYYVDYKNNLTRIPKASARWFQTIL  505
```
