# Supplementary material for: Genome-wide identification and functional characterization of natural antisense transcripts in Salvia miltiorrhiza
Source: Sci Rep. 2021 Feb 26;11:4769. doi: 10.1038/s41598-021-83520-6 (PMC7910453; doi:10.1038/s41598-021-83520-6)
Supplement: Supplementary file 7 — Supplementary Information. [file 41598_2021_83520_MOESM7_ESM.docx]

**Supplementary Tables and Figures**

# Title

**Genome-wide identification and functional characterization of natural antisense transcripts in** ***Salvia miltiorrhiza***

Mei Jiang^1^, Haimei Chen^1^, Jingting Liu^1^, Qing Du^1, 2^, Shanfa Lu^1#^, Chang Liu^1#^

^1^Key Laboratory of Bioactive Substances and Resource Utilization of Chinese Herbal Medicine from Ministry of Education, Engineering Research Center of Chinese Medicine Resources from Ministry of Education, Institute of Medicinal Plant Development, Chinese Academy of Medical Sciences, Peking Union Medical College, Beijing 100193, P. R. China.

^2^College of Pharmacy, Qinghai Nationalities University, Key Laboratory of Plant Resources of Qinghai-Tibet Plateau in Chemical Research, Xining, Qinghai 810007, China

^#^ Corresponding Authors: Shanfa Lu, +86-10-57833366 (tel), +86-10-57833366 (fax), [sflu@implad.ac.cn](mailto:sflu@implad.ac.cn) (email); Chang Liu, +86-10-57833111 (tel), +86-10-57833366 (fax), [cliu6688@yahoo.com](mailto:cliu6688@yahoo.com) (email).

Email Address

MJ: [mjiang0502@163.com](mailto:mjiang0502@163.com)

HMC: [hmchen@implad.ac.cn](mailto:hmchen@implad.ac.cn)

JTL: [liujingtingy@163.com](mailto:liujingtingy@163.com)

QD: 2017001@qhmu.edu.cn

SL: sflu@implad.ac.cn

CL: [cliu6688@yahoo.com](mailto:cliu6688@yahoo.com)

**Table S1 Summary of the RNA-seq data. p00: the three plants samples were pooled by tissue type, p01: plant 01, p02: plant 02, p03: plant 03. f: flower, l: leaf, r: root.**

| **Sample** | **Read** | **Base** |
| --- | --- | --- |
| p00_f | 25.3M | 5.1G |
| p00_l | 53.8M | 10.8G |
| p00_r | 26.7M | 5.3G |
| p01_f | 44.5M | 6.6G |
| p02_f | 43.4M | 6.5G |
| p03_f | 42.9M | 6.4G |
| p01_l | 42.5M | 6.3G |
| p02_l | 44.2M | 6.6G |
| p03_l | 41.6M | 6.2G |
| p01_r | 44.2M | 6.6G |
| p02_r | 41.9M | 6.2G |
| p03_r | 40.9M | 6.1G |

**Table S2 is provided in the excel sheet Sfile 1.**

**Table S3 Significantly enriched GO terms for the STs having *cis*-NATs in all three tissues analyzed.**

| **ID** | **Category** | **Term** | **Description** | **q value** |
| --- | --- | --- | --- | --- |
| 1 | GO Biological Processes | GO:0052542 | defense response by callose deposition | 0.020 |
| 2 | GO Biological Processes | GO:0051049 | regulation of transport | 0.021 |
| 3 | GO Biological Processes | GO:0031668 | cellular response to extracellular stimulus | 0.032 |
| 4 | GO Biological Processes | GO:0016050 | vesicle organization | 0.033 |
| 5 | GO Molecular Functions | GO:0043531 | ADP binding | 0.049 |

**Table S4 KEGG pathway for the STs having *cis*-NATs in all three tissues analyzed.**

| **KEGG pathway** | **ST ID** |
| --- | --- |
| Anthocyanin biosynthesis | ST0002 |
| beta-Alanine metabolism | ST0117 |
| Biosynthesis of antibiotics | ST0076 |
| Biosynthesis of secondary metabolites | ST0137, ST0023, ST0135, ST0076, ST0141, ST0002 |
| Carbon metabolism | ST0117 |
| Cutin, suberine and wax biosynthesis | ST0137 |
| Cyanoamino acid metabolism | ST0135 |
| Lipoic acid metabolism | ST0081 |
| Metabolic pathways | ST0081, ST0023, ST0117, ST0135, ST0087, ST0076, ST0141, ST0002 |
| Phenylpropanoid biosynthesis | ST0023, ST0135 |
| Plant-pathogen interaction | ST0145, ST0093, ST0138 |
| Propanoate metabolism | ST0117 |
| Protein processing in endoplasmic reticulum | ST0130, ST0114, ST0008 |
| Purine metabolism | ST0087, ST0076 |
| Pyrimidine metabolism | ST0087 |
| Riboflavin metabolism | ST0141 |
| RNA polymerase I | ST0087 |
| Starch and sucrose metabolism | ST0135 |
| Valine, leucine and isoleucine degradation | ST0117 |

**Table S5 The information of the 25 SAT pairs validated by ssRNA-seq experiments.**

| **Pair No.** | **NAT ID** | **ST ID** | **r** | **Condition** | **Go term or Kegg pathway** |
| --- | --- | --- | --- | --- | --- |
| 1 | NAT0001 | ST0001 | -0.35 | C2 | tanshinone biosynthesis |
| 2 | NAT0002 | ST0002 | 0.35 | C2 | Anthocyanin biosynthesis |
| 3 | NAT0003 | ST0003 | 0.09 | C2 | proteolysis, metabolic process |
| 4 | NAT0004 | ST0004 | 0.57 | C2 | metabolic process, flavonoid glucuronidation |
| 5 | NAT0005 | ST0005 | 0.95 | C1,C2 | transcription, DNA-templated |
| 6 | NAT0006 | ST0006 | 0.61 | C2 | regulation of transcription |
| 7 | NAT0007 | ST0007 | 0.33 | C2 | MAPK cascade, transcription |
| 8 | NAT0008 | ST0008 | 0.49 | C2 | regulation of COPII vesicle coating |
| 9 | NAT0009 | ST0009 | 0.30 | C2 | proteolysis, chloroplast organization, photosynthesis |
| 10 | NAT0010 | ST0010 | 1.00 | C1,C2 | carbohydrate metabolic process |
| 11 | NAT0011 | ST0011 | 0.93 | C1,C2 | phosphorylation |
| 12 | NAT0012 | ST0012 | 0.01 | C2 | syncytium formation, plant-type cell wall organization |
| 13 | NAT0013 | ST0013 | 0.94 | C1,C2 | defense response to oomycetes |
| 14 | NAT0014 | ST0014 | -0.39 | C2 | translational frameshifting |
| 15 | NAT0015 | ST0015 | 0.98 | C1,C2 | Defense mechanisms |
| 16 | NAT0016 | ST0016 | 0.77 | C2 | response to stress |
| 17 | NAT0017 | ST0017 | 0.06 | C2 | microtubule |
| 18 | NAT0018 | ST0018 | 0.95 | C1,C2 | Posttranslational modification |
| 19 | NAT0019 | ST0019 | 0.03 | C2 | cell cycle |
| 20 | NAT0020 | ST0020 | 0.32 | C2 | peptidyl-tyrosine dephosphorylation |
| 21 | NAT0021 | ST0021 | 1.00 | C1,C2 | protein phosphorylation |
| 22 | NAT0022 | ST0022 | -0.42 | C2 | membrane |
| 23 | NAT0023 | ST0023 | 0.60 | C2 | Phenylpropanoid biosynthesis |
| 24 | NAT0024 | ST0024 | 0.92 | C1 | NA |
| 25 | NAT0025 | ST0025 | 0.92 | C1 | NA |

r: the pearson correlation coefficient of the expression levels between NATs and the cognate STs. C1: condition 1, the expression profiles of NATs and parental genes were positively correlated with r values ≥ 0.9. C2: condition2, the parental genes of these NATs were mapped to the GO terms or the KEGG pathways.

**Table S6 is provided in the excel sheet Sfile 1.**

**Table S7 is provided in the excel sheet Sfile 1.**

**Table S8 is provided in the excel sheet Sfile 1.**


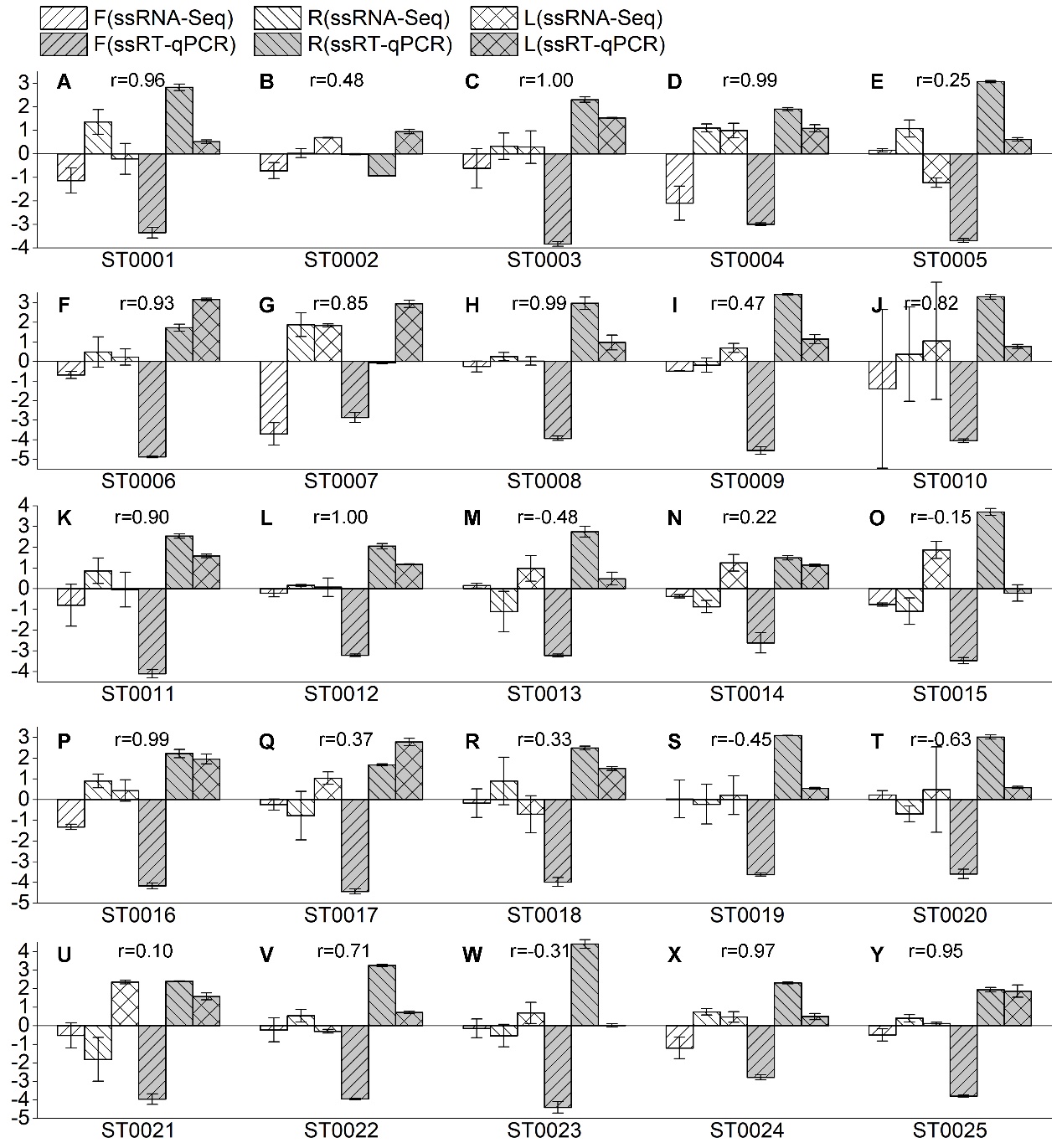


**Figure S1 Validation of the expression level of 25 STs in flower (F), root (R) and leaf (L) tissues of *S. miltiorrhiza*.**

X axis shows the analysis of expression levels in the three tissues using two methods, including ssRNA-seq and ssRT-qPCR. Y axis shows the relative expression levels normalized to the mean expression levels in the three tissues analyzed. Error bars represent the standard error. The values of r represent the correlation coefficient of expression levels between ssRNA-seq and ssRT-qPCR.
